# Clinical Services Provided by Pharmacists in Primary Health Care in Latin America: A Scoping Review

**DOI:** 10.1111/jep.70628

**Published:** 2026-11-04

**Authors:** Yasmin Silva Barrozo, Beatriz Freires Nascimento, Vanessa Souza Mendonça, Guacira Corrêa de Matos, Camila Campos Valerio, Livia Ribeiro Gomes, Fernanda Torres, Elisangela Costa Lima, Cesar Antunes Teixeira, Luis Phillipe Nagem Lopes, Alice Ramos‐Silva

**Affiliations:** ^1^ Faculty of Pharmacy Federal University of Rio de Janeiro Rio de Janeiro Brazil; ^2^ Library, Clementino Fraga Filho University Hospital and Thoracic Diseases Institute (HUCFF/IDT) Rio de Janeiro Brazil

**Keywords:** clinical services, Latin America, pharmacists, pharmacy services, primary health care, scoping review

## Abstract

**Objective:**

To map and characterise evidence on pharmacist‐provided clinical services in primary health care in Latin America, including service types, practice settings, study designs, team integration, populations, outcomes, and equity‐related dimensions.

**Methods:**

This scoping review followed JBI methodological guidance and PRISMA‐ScR. Bibliographic databases and grey literature sources were searched without language or publication date restrictions according to a prospectively registered protocol. Two reviewers independently screened records and charted data. Multiple classifications per study were permitted when applicable. Findings were synthesised descriptively and narratively.

**Results:**

We included 134 studies from six countries, 107 of which were conducted in Brazil. Among 140 study‐design classifications, randomised controlled trials (*n* = 26), cross‐sectional studies (*n* = 23), mixed‐methods studies (*n* = 21), and qualitative studies (*n* = 20) predominated. Among 137 study‐setting classifications, primary health care units (*n* = 80) and community pharmacies linked to primary care (*n* = 29) were most frequent. The 168 study‐service entries most commonly represented pharmacotherapeutic follow‐up (*n* = 54), health education (*n* = 35), medication review (*n* = 20), and clinically oriented dispensing (*n* = 19); seven could not be clearly classified. Service, process, or management indicators accounted for 87 of 182 study‐outcome classifications. Among 136 team‐integration entries, referral‐based (*n* = 54) and isolated (*n* = 35) models predominated. Only 26 studies reported an equity‐related variable.

**Conclusions:**

Most of the mapped studies were conducted in Brazilian primary health care settings, with comparatively few studies from other Latin American countries. Future research should examine service integration, equity, economic outcomes, and sustainability across diverse health‐system contexts in the region.

## 1 Introduction

Primary health care is a foundational component of health systems, organising first‐contact access, continuity of care, care coordination, and the rational use of resources [1]. Its strengthening is particularly important in Latin America, where chronic conditions, multimorbidity, medication‐related problems, and social inequalities coexist with fragmented care, limited coordination, and persistent workforce and infrastructure constraints [2, 3, 4, 5].

Pharmacists can contribute to patient care through clinical services designed to optimise pharmacotherapy, identify and prevent medication‐related problems, support medication adherence, and improve patient‐relevant outcomes [6]. Systematic reviews indicate that these services may improve prescribing quality and selected clinical outcomes, although the magnitude and consistency of their effects vary across intervention models, populations, and healthcare settings [7, 8]. In hospital settings, pharmaceutical care interventions have been associated with improvements in selected clinical parameters and shorter lengths of stay, although no statistically significant reduction in medical costs was demonstrated [7]. In primary care, pharmacist integration into general practice probably reduces potentially inappropriate prescribing and the number of medications used by patients with polypharmacy; however, evidence regarding health‐related quality of life, other patient‐important outcomes, and cost‐effectiveness remains uncertain [8]. Collectively, these findings support the potential value of pharmacist‐provided clinical services while underscoring the need to examine how they are implemented, integrated into healthcare teams, and evaluated across health‐system contexts. In primary health care, these services may include pharmacotherapeutic follow‐up, medication review, health education, medication reconciliation, therapeutic drug monitoring, management of minor ailments, and other direct‐care activities involving patients, families, or communities [9, 10].

Despite this growing evidence base, nationally standardised and reimbursed programmes remain uncommon. Medication review, medication reconciliation, and seamless care are often implemented through local or institution‐specific initiatives, potentially constraining their sustainability, scalability, and equitable access [9, 10, 11, 12]. Although implementation extends beyond primary care, in Slovenia, for example, medication reconciliation and seamless care have been progressively implemented and reimbursed through standardised procedures and shared medication records [13]. Case reports from a mental health hospital further illustrate how clinical pharmacists may identify medication omissions and other discrepancies during transitions of care [14]. More broadly, implementation is shaped by professional regulation, financing and reimbursement arrangements, workforce capacity, information infrastructure, and interprofessional integration [12, 13, 14, 15, 16, 17]. Consequently, similarly designated services may differ substantially across countries and practice settings in their scope, intensity, organisational integration, and sustainability. Mapping these variations is therefore necessary to identify which service models have been implemented, under what conditions, and where implementation and evidence gaps persist.

Given the breadth and heterogeneity of the available literature, a scoping review was considered more appropriate than a systematic review of effectiveness. The review was not designed to compare a predefined clinical pharmacy intervention with usual care or to estimate a pooled effect for a specific outcome. Instead, the available evidence encompasses diverse service models, practice settings, populations, implementation arrangements, study designs, and outcome measures, requiring an approach capable of mapping its extent, characteristics, and distribution [18, 19]. Accordingly, this scoping review aimed to map and characterise pharmacist‐provided clinical services in primary health care across Latin America, including their service typologies, practice settings, models of interprofessional integration, target populations, reported outcomes, implementation characteristics, and evidence gaps. This mapping may also inform future systematic reviews focused on sufficiently defined interventions, populations, and outcomes.

## 2 Methods

### 2.1 Study Design

We conducted a scoping review to map and characterise the extent, nature, and distribution of the evidence on clinical services provided by pharmacists in primary health care in Latin America. The review followed the JBI methodological guidance for scoping reviews [19] and was reported in accordance with the Preferred Reporting Items for Systematic Reviews and Meta‐Analyses extension for Scoping Reviews (PRISMA‐ScR) [20].

### 2.2 Protocol and Review Question

The protocol was developed a priori, prospectively registered in the Open Science Framework (OSF; registration ID: b2ec8), and subsequently published [21]. It prespecified the rationale, objectives, eligibility criteria, search strategy, study selection procedures, data charting process, and synthesis methods.

The review question and eligibility criteria were structured according to the Population–Concept–Context (PCC) framework. The Population comprised individuals, families, or communities receiving or intended to receive pharmacist‐provided care, without restrictions regarding age, sex, or clinical condition. Evidence sources could include patients, caregivers, pharmacists, other healthcare professionals, managers, or service‐level data, provided that they addressed an eligible clinical service intended for individuals, families, or communities. The Concept encompassed pharmacist‐provided clinical services involving a direct‐care component and intended to optimise medication use, prevent or resolve medication‐related problems, promote health, or improve patient‐relevant outcomes. The Context comprised primary health care settings in Latin American countries.

Accordingly, the review sought to answer the following question: What evidence is available regarding the extent, nature, and characteristics of clinical services provided by pharmacists in primary health care across Latin America?

Primary health care was operationally defined as first‐contact, outpatient, community‐based care delivered close to people's everyday environment and formally linked to a health system. This definition was based on international conceptualisations of primary health care as care organised around people's needs, delivered as close as feasible to everyday settings, and functioning as a first point of contact with the health system [22, 23].

Eligible practice settings included primary health care units, general or family practice services, Family Health Strategy services, ambulatory primary care settings, community pharmacies explicitly linked to primary care, mixed settings containing an identifiable primary care component, and other primary care settings. Primary health care units were defined as fixed outpatient facilities providing first‐contact care, health promotion, disease prevention, chronic disease follow‐up, primary clinical care, and referral to other levels of care when needed [23]. Family Health Strategy services were defined as team‐based Brazilian primary care services organised around a defined territory and population, with longitudinal follow‐up of individuals and families [24]. Ambulatory primary care settings were defined as outpatient, non‐hospital services providing primary care for common acute or chronic health needs without inpatient admission [25].

Community pharmacies linked to primary care were defined as pharmacy settings in which pharmacist‐provided clinical services were connected to primary care through referral, collaborative care arrangements, institutional programmes, shared documentation, or another explicit linkage with health‐system services. Community pharmacies functioning solely as retail medicine outlets were not considered primary care settings unless a clinical service linked to primary care was clearly described. General or family practice services were included and classified as primary health care units, ambulatory primary care settings, or other primary care settings according to the organisation described in each study.

Eligibility and setting classification were assessed separately. Studies were excluded when the available information was insufficient to establish that the service was delivered in, or explicitly linked to, primary health care. When the primary health care context was clear but the specific setting category could not be determined, the study was included and classified as ‘other primary care setting’.

### 2.3 Eligibility Criteria

Sources were eligible if they: (i) reported empirical data; (ii) were conducted in at least one Latin American country; (iii) described the provision, implementation, organisation, or evaluation of at least one pharmacist‐provided clinical service involving a direct‐care component; (iv) were conducted in primary health care or in a setting explicitly linked to primary care; and (v) provided sufficient information for eligibility assessment and data charting.

We included primary studies regardless of methodological design, including randomised and non‐randomised studies, observational studies, qualitative studies, mixed‐methods studies, pilot studies, programme evaluations, empirical case or experience reports, theses, and dissertations. Studies conducted in more than one healthcare setting were eligible when the primary care component could be clearly identified or when data relevant to primary care could be extracted separately. Comparative studies were eligible, but a comparator was not required because the review aimed to map service models, practice settings, implementation characteristics, interprofessional integration, and reported outcomes rather than to estimate comparative effectiveness. Requiring usual care or non‐clinical pharmacy practice as a comparator would have excluded relevant descriptive, qualitative, and implementation evidence.

Because terminology in pharmacy practice research is heterogeneous, studies were considered eligible when a clinical component of pharmacist practice was clearly described. Eligible services included, but were not limited to, pharmacotherapeutic follow‐up, medication review, health education, medication reconciliation, therapeutic drug monitoring, health screening, management of minor ailments, health condition management, and clinically oriented dispensing.

Pharmacists and clinical pharmacists were not treated as separate professional categories. Eligibility was determined by the clinical nature of the service described rather than by professional title, postgraduate training, certification, or practice setting. Professionals were considered pharmacists when identified as such by the original source. The term ‘clinical pharmacist’ was retained only when explicitly reported by the study authors and was not independently assigned by the reviewers.

The review was not restricted to community pharmacy settings. Services delivered in general or family practice and other first‐contact primary care services were eligible. Nursing homes and long‐term care facilities were eligible only when the pharmacist‐provided service was explicitly integrated into a primary health care pathway.

We excluded publications focused exclusively on medicine procurement, stock management, administrative pharmaceutical services, or dispensing without a clinical component. We also excluded studies conducted exclusively in hospital inpatient, tertiary care, emergency, nursing home, or long‐term care settings when the service was not explicitly integrated into primary health care. Reviews, editorials, letters, commentaries, opinion papers, and other non‐empirical narrative publications were excluded. No restrictions were applied regarding language, publication date, or publication status, provided that sufficient empirical information was available for eligibility assessment and data charting.

### 2.4 Information Sources

The search strategy was developed by a trained health sciences librarian and reviewed by the authors for conceptual adequacy and alignment with the review question. Electronic searches were conducted in Biblioteca Virtual em Saúde, including LILACS and related regional indexed sources, SciELO, PubMed/MEDLINE, Embase, Scopus, and CINAHL.

Grey literature searches were conducted in ProQuest Dissertations & Theses Citation Index, the CAPES Theses and Dissertations Catalogue, the Brazilian Digital Library of Theses and Dissertations, Open Access Theses and Dissertations, EBSCO Open Dissertations, and OAIster. We also screened the reference lists of included studies and relevant reviews and performed citation tracking when appropriate.

Theses and dissertations were deliberately included because the review aimed to comprehensively map the regional evidence, including locally conducted service evaluations and implementation studies that may not have been published in indexed journals. Their inclusion also reduced the risk of omitting evidence because of publication status. These sources were required to provide full‐text empirical information, meet the same eligibility criteria as journal articles, and contain sufficient methodological and contextual detail for data charting. Source type was recorded to distinguish theses and dissertations from peer‐reviewed journal publications.

The original searches were conducted on 10 September 2025 and were fully updated on 7 April 2026 using the same conceptual structure and information sources.

### 2.5 Search Strategy

The search strategy was structured around three conceptual blocks: primary health care, pharmacy services or pharmacists, and Latin America or country‐specific terms. Controlled vocabulary and free‐text terms were combined to maximise sensitivity. Controlled vocabulary included database‐specific subject headings, such as MeSH in PubMed and Emtree in Embase. Free‐text terms were included in English, Portuguese, and Spanish to account for multilingual publication and variation in the terminology and indexing of pharmacy practice research in Latin America.

The primary care block included terms such as ‘Primary Health Care’, ‘Primary Care’, ‘Community Health Services’, ‘Ambulatory Care’, ‘Outpatient Care’, ‘Family Practice’, and ‘General Practice’, together with Portuguese and Spanish equivalents where applicable. The pharmacy block included terms such as ‘Pharmaceutical Services’, ‘Pharmacy Services’, ‘Community Pharmacy Services’, ‘pharmaceutical care’, ‘clinical pharmacy’, ‘pharmacist’, and ‘pharmacist interventions’ also expanded with Portuguese and Spanish equivalents. Although the exact phrases ‘general practice clinical pharmacy’ and ‘primary care clinical pharmacy’ were not used as standalone expressions in every database, their constituent concepts were captured by combining primary care or general practice terms with pharmacist and clinical pharmacy terms and adapting the syntax to each database. The geographic block included regional descriptors such as ‘Latin America’, ‘South America’, and ‘Central America’, as well as the names of individual Latin American countries. Full reproducible search strategies for all databases and grey literature sources are provided in Supporting Information S1.

Grey literature was defined as research output not controlled by traditional commercial journal publishing channels, including theses, dissertations, repository records, and other academic documents retrievable through specialised databases and institutional platforms. Because indexing is less consistent in these sources, broader search expressions were used when necessary to maximise retrieval.

### 2.6 Selection of Sources of Evidence

All records retrieved from bibliographic databases, grey literature sources, and supplementary searches were imported into Rayyan for screening. Potential duplicates were identified using the platform's duplicate‐detection function and manually verified by comparing title, authors, publication year, source, and other bibliographic characteristics before removal.

Study selection was conducted in two sequential stages: title and abstract screening, followed by full‐text assessment. Both stages were performed independently by two reviewers. Before formal screening, the reviewers applied the eligibility criteria to a pilot sample of records to improve consistency in study selection.

During title and abstract screening, disagreements were resolved through discussion and consensus or, when uncertainty remained, by retaining the record for full‐text assessment. The full texts of potentially eligible records were subsequently retrieved and assessed against the eligibility criteria. Disagreements during full‐text assessment were resolved through discussion and consensus, with adjudication by a third reviewer when required. Reasons for exclusion at the full‐text stage were recorded and are presented in Supporting Information S2.

When multiple reports described the same underlying study, the reports were linked and treated as a single study for synthesis. Information was integrated across reports to obtain the most complete study‐level record while retaining the bibliographic details of all associated reports.

### 2.7 Data Charting Process

Data were charted independently by two reviewers using a standardised form developed a priori and piloted before formal extraction. Following independent charting, the two datasets were compared, and discrepancies were resolved through discussion and consensus, with adjudication by a third reviewer when required.

During the early stages of data charting, the form was refined to improve clarity and consistency. When modifications were introduced, previously charted records were revisited to ensure uniform coding. When essential information was unclear or unavailable, the full text and Supporting materials were rechecked. Information that remained unavailable after verification was coded as not reported.

### 2.8 Data Items and Operational Definitions

For each included source, we extracted bibliographic information, including author, publication year, country, language, and source type; methodological characteristics, including study design, sample size, reported study‐conduct or data‐collection period, intervention duration, timing of outcome assessment, and post‐intervention follow‐up when these could be explicitly distinguished; practice setting; target population and age group; and the original terminology used by the authors to describe the clinical service.

We also extracted service components; methods, tools, or frameworks used in service delivery; mode of delivery when reported; model of integration with the healthcare team; barriers and facilitators to implementation, delivery, or evaluation; outcome domains; and equity‐related variables. Categories were non‐mutually exclusive; therefore, an individual study could contribute to more than one category. Participation in multidisciplinary ward rounds was not prespecified or extracted as a separate variable because the review focused on primary health care and excluded inpatient‐only studies, where ward rounds are most commonly undertaken. Interprofessional integration was assessed through activities applicable to primary care, including referral and communication pathways, case discussions, shared decision‐making, joint care, and shared clinical records.

Practice setting was categorised as a primary health care unit, community pharmacy linked to primary care, Family Health Strategy service, ambulatory primary care setting, mixed setting, or other primary care setting.

Because terminology in pharmacy practice research is inconsistent across studies and settings, we retained the original service terminology reported by each source and applied a standardised classification to improve comparability. Service categories were defined using an operational typology based on the Brazilian Federal Pharmacy Council framework for pharmacist services delivered directly to patients, families, and communities [26]. The categories included health screening, health education, clinically oriented dispensing, management of minor ailments, therapeutic drug monitoring, medication reconciliation, medication review, health condition management, and pharmacotherapeutic follow‐up. When a source reported a sufficiently described clinical service that did not correspond to any of the prespecified categories, the service was coded as ‘other’. When the description was insufficient to determine the nature of the service, it was coded as ‘not reported or insufficiently described for classification’.

The Brazilian framework was selected because it provides explicit service‐level categories aligned with the unit of analysis of this review, while remaining consistent with the broader roles and functions described in the FIP/WHO Good Pharmacy Practice guidance [27]. Its selection reflected the analytical granularity required to harmonise heterogeneous service terminology and did not imply superiority over international frameworks. Given the regional scope of the review, the framework was used solely as an analytical tool for harmonisation and comparison, not as a normative classification for Latin America.

For this review, pharmacotherapeutic follow‐up was operationally defined as a longitudinal, patient‐centred service involving multiple pharmacist–patient encounters; assessment of the patient's health conditions and overall pharmacotherapy; identification of medication‐related problems; development and implementation of a care plan; documented interventions; and subsequent monitoring or reassessment of therapeutic outcomes. Medication review alone was insufficient for classification as pharmacotherapeutic follow‐up. A stand‐alone medication review was classified as medication review unless it was embedded within a clearly described longitudinal care process involving planned follow‐up and outcome reassessment [26].

Two reviewers independently examined the description of each reported service and mapped it to one or more categories according to explicit criteria: the stated objective of the service, its core activities, and the care process described in the source. The original terminology used by the study authors was retained in the extraction form, whereas the harmonised classification was applied during synthesis. When a source reported more than one eligible service, all applicable categories were recorded. Disagreements were resolved through discussion and consensus, with adjudication by a third reviewer when necessary.

Outcome domains were classified using an adapted Economic, Clinical, and Humanistic Outcomes (ECHO) framework, originally proposed for pharmacoeconomic and outcomes research in pharmacy. The framework comprised clinical, humanistic, and economic outcomes, with an additional domain for service, process, or management indicators to capture implementation and operational measures frequently used in evaluations of pharmacist‐provided primary care services [28, 29].

Clinical outcomes included measures of health status, disease control, symptoms, laboratory parameters, medication‐related problems, and adverse drug events. Humanistic outcomes included patient‐reported or patient‐perceived effects of care, such as satisfaction, experience, and health‐related quality of life. Economic outcomes included costs, healthcare resource use, and cost‐effectiveness measures. Service, process, or management indicators included measures related to implementation, workflow, pharmacist activities, interprofessional integration, and operational performance.

Equity‐related variables were charted using the PROGRESS‐Plus framework as an organising structure for dimensions associated with social disadvantage and differential access to care [30]. We recorded whether studies reported place of residence; race, ethnicity, culture, or language; occupation; sex or gender; religion; education; socioeconomic status; social capital; and additional relevant dimensions, such as age and disability.

Because scoping reviews aim to map the extent and nature of reporting, we recorded both the presence of these variables and, when applicable, whether they were incorporated analytically through stratification, subgroup description, adjusted analysis, or interpretation of findings. No imputation of missing information was performed.

Reported barriers and facilitators were charted when explicitly described by the study authors. These data were extracted at the study level and subsequently grouped thematically according to their primary focus, including methodological and evaluation issues, organisational and structural factors, patient‐related factors, interprofessional or institutional factors, workforce and training issues, and contextual or external influences. A single study could contribute to more than one barrier or facilitator category when appropriate.

### 2.9 Critical Appraisal

No formal critical appraisal or assessment of certainty of the evidence was undertaken because the purpose of the scoping review was to map and characterise the available evidence rather than to estimate intervention effectiveness, determine pooled effects, or formulate clinical recommendations. Theses and dissertations therefore contributed to the descriptive mapping of the evidence but were not interpreted as providing the same evidential certainty as peer‐reviewed comparative effectiveness studies.

### 2.10 Synthesis of Results

Charted data were synthesised using descriptive statistics and narrative synthesis. Absolute frequencies and percentages were calculated for country, practice setting, study design, service category, model of team integration, age group, outcome domain, equity‐related variables, and reported barriers and facilitators.

When more than one classification was explicitly applicable, a single study could contribute to multiple practice settings, study‐design categories, service categories, team‐integration models, study‐population categories, outcome domains, equity‐related dimensions, barriers, or facilitators. Consequently, category totals could exceed the total number of included studies. Denominators were specified in each table and figure according to the unit of analysis and comprised either individual studies or study–category contributions, including study–setting, study–design, study–service, study–integration, study–population, study–outcome, and study–equity entries. Percentages were calculated using the corresponding denominator reported for each analysis. Missing or insufficiently described information was retained and reported as ‘not reported’.

Reported study‐conduct or data‐collection periods were standardised to months when sources provided an explicit duration or sufficiently precise calendar dates. Durations reported in years were multiplied by 12, and durations reported in weeks were divided by 4.345. Month‐based calendar intervals were calculated inclusively. Year‐only ranges were converted as (final year − initial year + 1) × 12 and were regarded as coarse estimates. When linked reports described the same study, a duration was retained only when all reported estimates could be converted and agreed; partially reported or conflicting estimates were coded as not reported. One explicitly reported non‐contiguous data‐collection period was calculated by summing its component intervals. Records with missing, internally inconsistent, or otherwise insufficiently reported periods were not converted.

Because the reported study‐conduct or data‐collection period could encompass recruitment, intervention delivery, outcome assessment, or other data‐collection activities, it was analysed separately from intervention duration, timing of outcome assessment, and post‐intervention follow‐up. A sensitivity analysis excluded periods reported only as calendar years and the non‐contiguous period. For descriptive purposes, outcome assessment at 12 months or longer was considered longer‐term; this threshold was not used to infer sustained effectiveness.

The temporal trend in the annual number of publications was assessed using the Mann–Kendall test. For studies represented by multiple linked reports, the earliest publication year was used to avoid double counting. Years with no publications were retained in the annual series. Statistical analyses and figures were produced using R, version 4.6.0, with two‐sided statistical significance set at *p* < 0.05.

Narrative synthesis summarised the dominant patterns in the literature and related them to the review objectives. The synthesis focused on the geographical distribution of the evidence; the distribution of studies by design and setting; the most frequently reported service categories; patterns of interprofessional integration; clinical, humanistic, economic, and service‐related outcome reporting; intervention duration, timing of outcome assessment, and follow‐up when available; the extent to which equity dimensions were incorporated; and the main barriers and facilitators reported for the implementation and delivery of pharmacist‐provided clinical services.

Results were presented through a PRISMA flow diagram, descriptive tables, and summary figures.

## 3 Results

### 3.1 Study Selection

The updated search identified 2201 records, comprising 1601 records from bibliographic databases and 600 from grey literature sources. After the removal of 818 duplicates, 1383 records underwent title and abstract screening, of which 676 were excluded. The full texts of 707 potentially eligible reports were sought; seven could not be retrieved, and 700 were assessed against the eligibility criteria. Of these, 566 reports were excluded: 247 were conducted outside primary health care, 175 did not involve pharmacists, 93 did not describe an eligible pharmacist‐provided clinical service, 40 were not primary studies, seven were conducted outside Latin America, three provided insufficient information for data charting, and one had no recorded reason for exclusion. Overall, 134 studies were included in the review. Of these, 18 (13.4%) were identified through thesis and dissertation repositories (Figure 1; Supporting Information S2 and S3).

**FIGURE 1:**
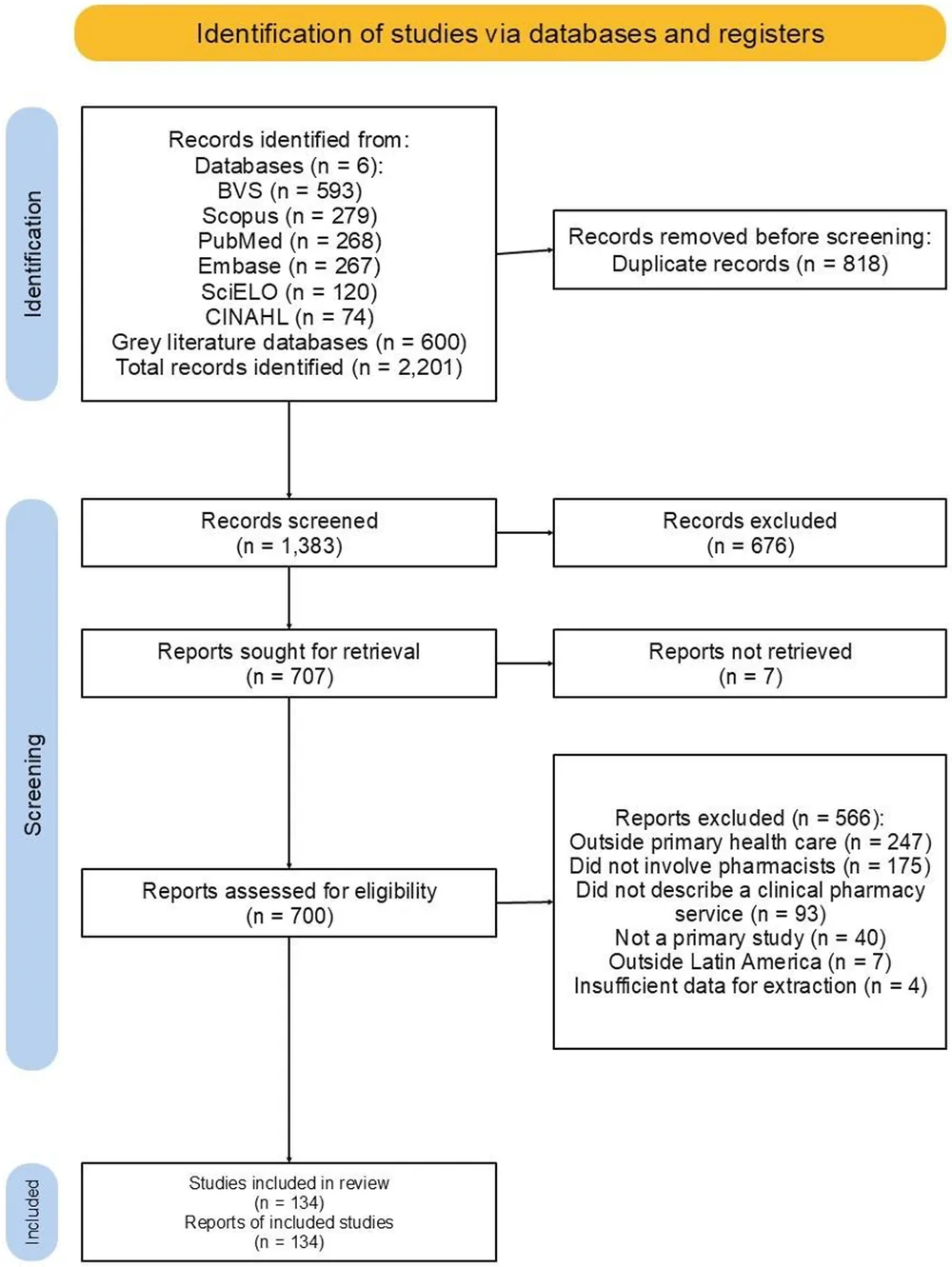
PRISMA 2020 flow diagram of the study selection process for the scoping review of clinical pharmacy services in primary health care in Latin America.

### 3.2 Bibliometric and Geographic Characteristics

Publication years ranged from 1974 to 2025. For studies represented by multiple linked reports, the earliest publication year was used as the anchor year. The annual distribution was uneven, with the highest numbers of studies published in 2022 (*n* = 15) and 2023 (*n* = 13). The years 2013, 2017, 2020, and 2021 each contributed eight studies. The annual number of included studies showed an increasing temporal trend over the study period (Mann–Kendall τ = 0.70; *p* < 0.001; Figure 2A).

**FIGURE 2:**
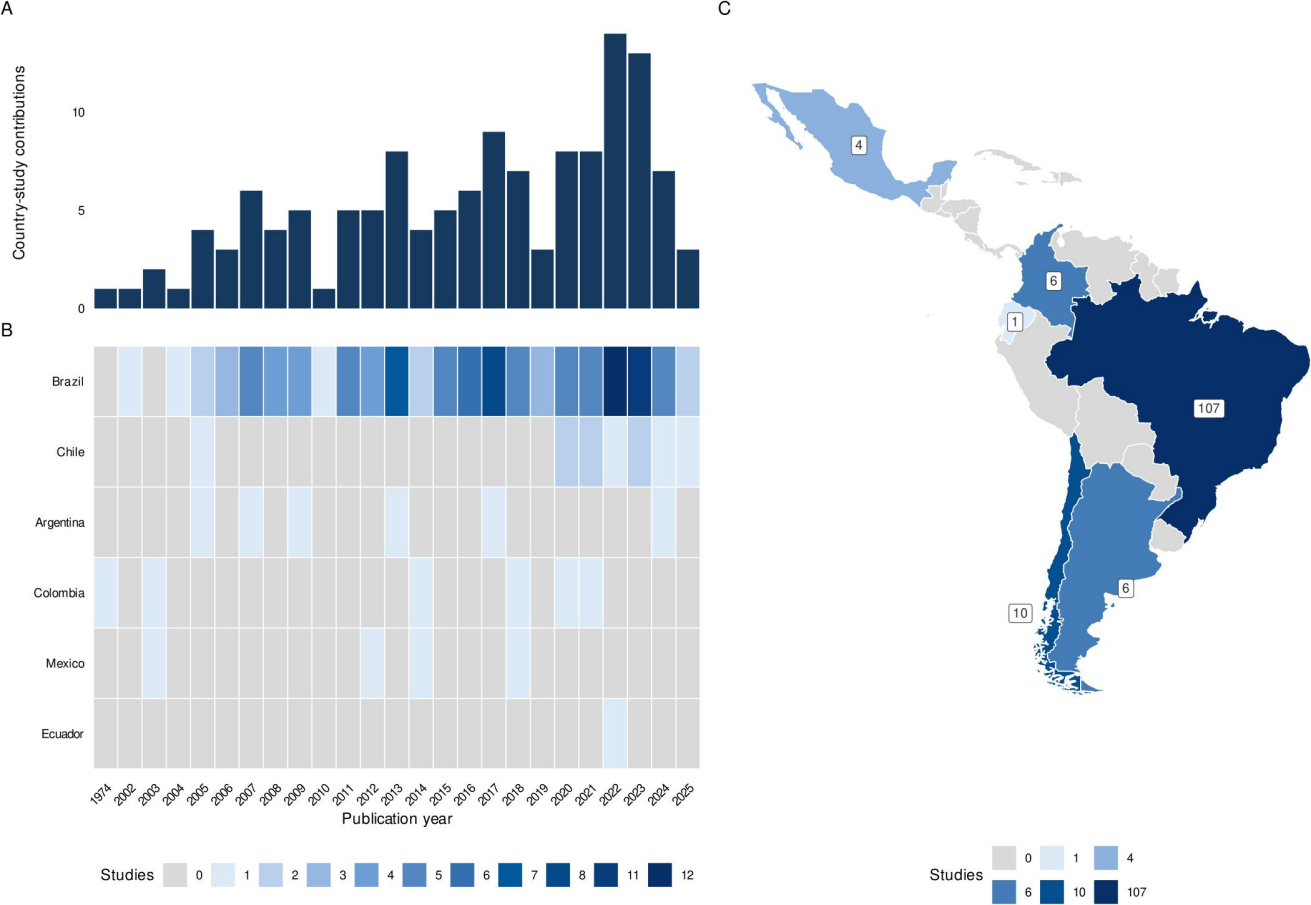
Temporal and geographic distribution of the 134 included studies. Panel A presents the annual number of studies according to the earliest publication year among linked reports, including years with no publications. Panel B presents the distribution of studies by country and publication year, and Panel C presents the total number of studies by country.

The geographic distribution was markedly uneven. Most studies were conducted in Brazil (*n* = 107; 79.9%), followed by Chile (*n* = 10; 7.5%), Argentina (*n* = 6; 4.5%), Colombia (*n* = 6; 4.5%), Mexico (*n* = 4; 3.0%), and Ecuador (*n* = 1; 0.7%) (Figure 2B,C).

### 3.3 Study Designs, Settings, and Populations

The 134 studies generated 140 design classifications because some studies incorporated more than one methodological design. Randomised controlled trials were the most frequent design (*n* = 26; 18.6%), followed by cross‐sectional studies (*n* = 23; 16.4%), mixed‐methods studies (*n* = 21; 15.0%), and qualitative studies (*n* = 20; 14.3%). Non‐randomised or quasi‐experimental studies accounted for 13 classifications (9.3%), case or experience reports for 11 (7.9%), before‐and‐after studies for 10 (7.1%), prospective cohort studies for nine (6.4%), retrospective cohort studies for four (2.9%), methodological studies for two (1.4%), and case series for one (0.7%).

Across 137 practice‐setting classifications, primary health care units were the most frequent setting (*n* = 80; 58.4%), followed by community pharmacies explicitly linked to primary care (*n* = 29; 21.2%), other primary care settings (*n* = 13; 9.5%), Family Health Strategy services (*n* = 7; 5.1%), ambulatory primary care settings (*n* = 6; 4.4%), and mixed settings containing an identifiable primary care component (*n* = 2; 1.5%). No included study was conducted in a nursing home or long‐term care facility.

The studies generated 142 target‐population classifications. Services directed toward the general population were most frequent (*n* = 65; 45.8%), followed by services for people with hypertension (*n* = 24; 16.9%), diabetes (*n* = 21; 14.8%), or polypharmacy (*n* = 10; 7.0%). The target population was not reported in 12 classifications (8.5%). Less frequently represented populations included people with cardiovascular diseases (*n* = 3; 2.1%), mental disorders (*n* = 2; 1.4%), pregnant women (*n* = 2; 1.4%), autoimmune diseases (n = 1; 0.7%), asthma (*n* = 1; 0.7%), and tobacco use (*n* = 1; 0.7%).

Regarding age groups, 38 studies (28.4%) focused on adults, 29 (21.6%) on older adults, and 20 (14.9%) included mixed age groups. One study (0.7%) focused on a paediatric population. Age group was not reported in 46 studies (34.3%) (Supporting Information S3).

### 3.4 Characteristics of Pharmacist‐Provided Clinical Services

The 134 studies generated 168 study–service entries because individual studies could report more than one service. Pharmacotherapeutic follow‐up was the most frequently identified service (*n* = 54; 32.1%), followed by health education (*n* = 35; 20.8%), medication review (*n* = 20; 11.9%), and clinically oriented dispensing (*n* = 19; 11.3%). A further 19 entries (11.3%) described sufficiently detailed clinical services that did not correspond to any category in the prespecified typology and were therefore classified as ‘other’.

Therapeutic drug monitoring was identified in seven entries (4.2%), medication reconciliation in three (1.8%), health screening in two (1.2%), and management of minor ailments in two (1.2%). Seven entries (4.2%) were insufficiently described to support classification into a specific service category.

### 3.5 Integration With the Health Team

The studies generated 136 team‐integration entries. Referral‐based models, characterised by written referrals or occasional telephone contact with other healthcare professionals, were most frequent (*n* = 54; 39.7%). Isolated practice, in which the pharmacist provided the service without documented interaction with the broader healthcare team, accounted for 35 entries (25.7%).

Collaborative models involving case discussion, bidirectional communication, or shared clinical decision‐making were identified in 20 entries (14.7%). Integrated or transdisciplinary models involving joint care, regular team participation, or real‐time access to shared clinical records accounted for 16 entries (11.8%). The model of integration was not reported in 11 entries (8.1%).

### 3.6 Outcome Domains

The studies generated 182 study–outcome classifications because individual studies could report outcomes from more than one domain. Service, process, or management indicators predominated (*n* = 87; 47.8%), followed by humanistic outcomes (*n* = 43; 23.6%) and clinical outcomes (*n* = 41; 22.5%). Economic outcomes were reported in six classifications (3.3%), whereas five classifications (2.7%) lacked a clearly identifiable outcome domain.

Figure 3 cross‐classifies outcome domains by study design. Because individual studies could contribute to multiple design and outcome categories, the figure contains 189 study–design–outcome contributions.

**FIGURE 3:**
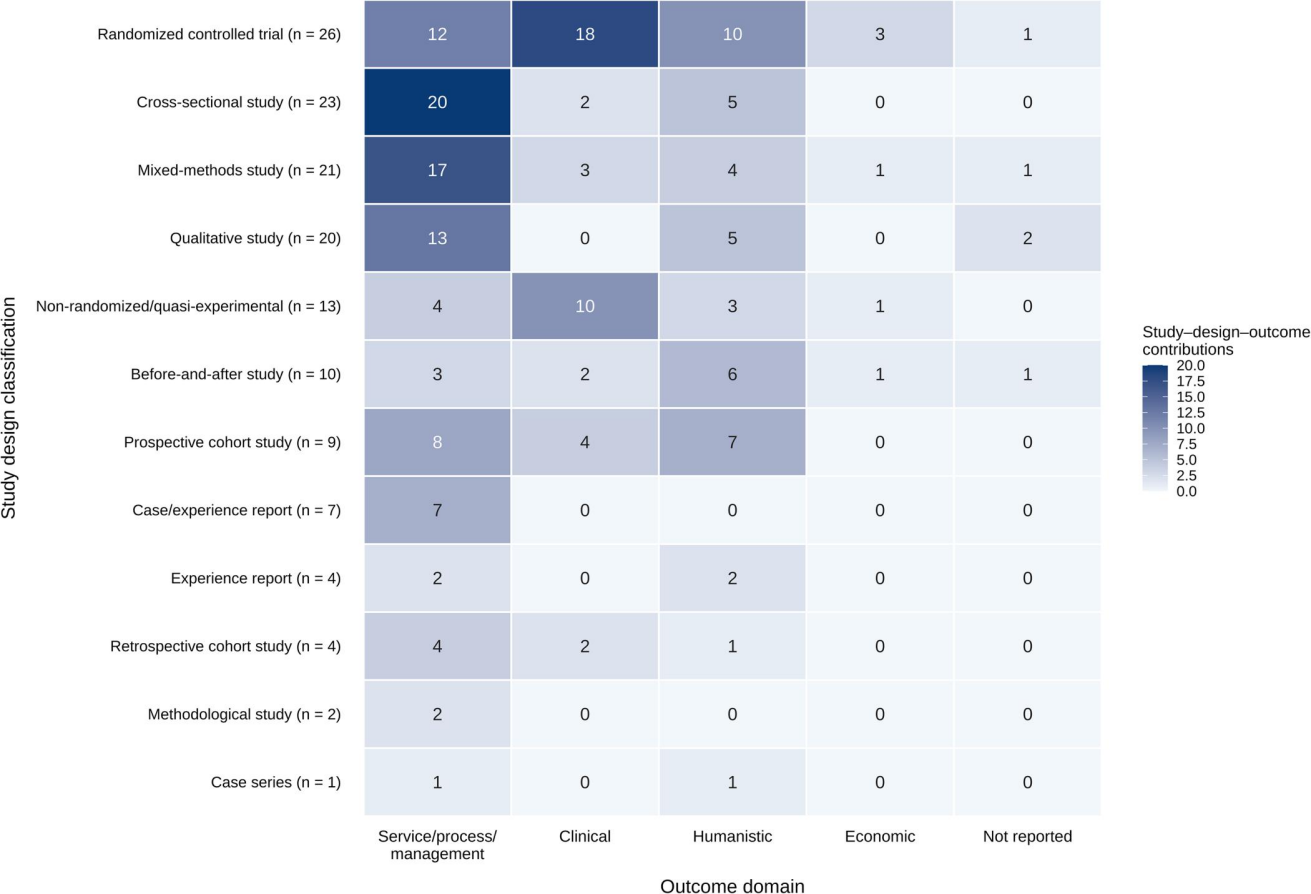
Distribution of outcome types across study designs in included studies. Distribution of outcome domains across study designs. The unit of analysis is the study–design–outcome contribution; therefore, individual studies may contribute to more than one cell.

### 3.7 Study‐Conduct Periods and Outcome‐Assessment Timing

Study‐conduct or data‐collection periods could be standardised to months for 100 of the 134 studies (74.6%). Among these studies, the median estimated period was 12 months (interquartile range, 6.0–21.3 months; range, 1–58 months). Fifty‐eight studies (58.0%) had study‐conduct periods of 12 months or less, whereas 42 (42.0%) had periods exceeding 12 months and 19 (19.0%) had periods exceeding 24 months.

In a sensitivity analysis excluding periods reported only as calendar years and records containing non‐contiguous study periods, the median duration was 11 months (interquartile range, 6–19 months; range, 1–58 months; *n* = 92).

Explicit timing of outcome assessment was identifiable in the charted outcome descriptions of only six studies (4.5%). One study assessed outcomes at 60 days, one at 6 months, three at 12 months, and one at 36 months. Using 12 months or longer as a descriptive threshold, four studies (3.0%) reported longer‐term outcome assessment.

These assessments generally occurred during active delivery of the pharmacist‐provided service. No charted study clearly distinguished a post‐intervention follow‐up period conducted after the service had ended. Consequently, the persistence of reported outcomes beyond active service delivery could not be consistently assessed.

### 3.8 Equity‐Related Variables

Only 26 of the 134 studies (19.4%) reported at least one equity‐related variable. These studies generated 37 equity classifications: education (*n* = 12), disability (*n* = 9), age (*n* = 8), socioeconomic status (*n* = 4), place of residence (*n* = 3), and sex or gender (*n* = 1). Most studies neither reported equity‐related variables nor incorporated them analytically through stratification, subgroup description, adjusted analysis, or interpretation of findings.

### 3.9 Barriers to Implementation

Among the 134 included studies, 69 (51.5%) explicitly reported at least one barrier to the implementation, delivery, or evaluation of pharmacist‐provided clinical services. Because barriers were classified using non‐mutually exclusive categories, individual studies could contribute to more than one category.

Methodological and evaluation limitations were the most frequently identified barriers (*n* = 35; 50.7% of the 69 studies reporting barriers), followed by organisational and structural constraints (*n* = 20; 29.0%), patient‐related barriers (*n* = 18; 26.1%), and interprofessional or institutional barriers (*n* = 17; 24.6%). Workforce and training barriers were identified in 10 studies (14.5%), whereas contextual or external barriers were reported in six (8.7%).

Methodological and evaluation barriers included small samples, short observation periods, missing data, absence of control groups or randomisation, indirect outcome assessment, and limited generalisability. Organisational and structural barriers included insufficient time, high workload, inadequate infrastructure, shortages of materials, and documentation problems. Patient‐related barriers primarily involved low medication adherence, limited educational attainment or health literacy, socioeconomic constraints, and missed follow‐up visits. Interprofessional and institutional barriers included limited integration with healthcare teams, inadequate communication with prescribers, restricted pharmacist autonomy, and insufficient institutional support. Contextual or external barriers included pandemic‐related disruptions and geographic constraints. Detailed study‐level classifications are provided in Supporting Information S4.

### 3.10 Facilitators to Implementation

Forty‐one of the 134 included studies (30.6%) explicitly reported at least one facilitator of the implementation, delivery, or evaluation of pharmacist‐provided clinical services. Because facilitator categories were non‐mutually exclusive, individual studies could contribute to more than one category; consequently, category totals exceed the 41 studies reporting facilitators.

Organisational and structural facilitators were the most frequently reported (*n* = 14; 34.1% of the 41 studies), followed by workforce and training facilitators (*n* = 13; 31.7%). Patient‐related or community facilitators and interprofessional or institutional facilitators were each identified in 10 studies (24.4%). Methodological and evaluation facilitators were reported in nine studies (22.0%), whereas policy, financial, or contextual facilitators were identified in five (12.2%).

Organisational and structural facilitators included dedicated counselling areas, simple or low‐cost technologies, appointment scheduling, opportunities for home follow‐up, and integration into existing services. Workforce and training facilitators included prior professional training, structured educational support, communication skills, and technical support from academic partners. Patient‐related and community facilitators included acceptance of the service, social support networks, community engagement, and adapted educational strategies, including pictograms.

Interprofessional and institutional facilitators included collaboration with healthcare teams, recognition of the pharmacist's clinical role, and improved communication with prescribers. Methodological and evaluation facilitators included the use of simulated patients, access to prescription and medical‐record data, longer observation periods, and systematic assessment tools. Policy, financial, and contextual facilitators included external funding and supportive policy or implementation environments. Detailed study‐level classifications are provided in Supporting Information S5.

## 4 Discussion

This scoping review mapped 134 studies of pharmacist‐provided clinical services in primary health care in Latin America and identified a geographically uneven and heterogeneous evidence base. Brazil contributed 107 studies (79.9%), and primary health care units were the most frequently represented setting. Among 168 study–service entries, pharmacotherapeutic follow‐up, health education, medication review, and clinically oriented dispensing predominated. Service, process, or management indicators accounted for almost half of the 182 outcome classifications, whereas economic outcomes, equity‐related variables, and collaborative or integrated models of care were less frequently documented. These findings indicate that the published literature has expanded but remains uneven in its geographic distribution, characterisation of services, organisational integration, and evaluative scope.

Brazil's predominance should be interpreted as evidence of a substantially larger published evidence base, not necessarily broader broader national coverage or greater maturity of pharmacist‐provided clinical services. Several contextual developments may have contributed to both service implementation and its documentation. Qualifar‐SUS established a national programme for strengthening pharmaceutical services within the Brazilian Unified Health System through investments in service qualification, infrastructure, education, information, and care‐related activities [31]. Professional and legislative developments subsequently broadened the formal possibilities for pharmacists' clinical roles and strengthened recognition of pharmacies as healthcare delivery settings, although implementation gaps remain [32, 33]. More recent analyses have also documented federal financing and organisational initiatives intended to strengthen pharmaceutical care within the SUS [34]. Nevertheless, programme‐based financing for infrastructure and service qualification does not imply uniform reimbursement for individual clinical services. Implementation remains decentralised and may vary substantially across municipalities and healthcare settings. Brazil's population size, postgraduate and research capacity, production of grey literature, and visibility in regional databases may also have influenced publication volume. Because these factors were not comparatively evaluated across countries, they should be regarded as plausible contextual explanations rather than causal findings. The observed distribution therefore reflects an imbalance in documented evidence and does not establish that services are absent or less developed elsewhere in Latin America.

Evidence from Europe provides a contextual policy comparison. In Austria, recent evidence concerning pharmacist‐led prescribing has identified limitations related to legal frameworks, organisational readiness, access to clinical information, and professional preparation [35]. In neighbouring Slovenia, medication review by clinical pharmacist consultants has been integrated into general practice and nationally reimbursed [36]. A nationally reimbursed programme of pharmacist‐led medication reconciliation and seamless care has also been implemented across hospitals and community pharmacies [13], while case reports from a mental health hospital illustrate the identification and resolution of medication omissions and other discrepancies during transitions of care [14]. More recently, a prospective non‐randomised national pilot of collaborative pharmacist prescribing in primary care reported improvements in health‐related quality of life, adherence to treatment guidelines, and predefined clinical outcomes [37]. Complementary qualitative research described generally positive experiences among patients, general practitioners, and pharmacist prescribers, while identifying legislation, reimbursement, and structured education as requirements for wider implementation [38]. Because these studies were conducted outside Latin America and included settings beyond primary health care, they are presented as contextual examples rather than as evidence directly comparable with the studies included in this review. Collectively, they illustrate that financial resources alone do not ensure implementation. Supportive legislation, reimbursement, workforce preparation, information continuity, and coordinated implementation are also important.

Medication reconciliation accounted for only three of the 168 study–service entries (1.8%) in the present review, indicating that it is not yet widely documented among pharmacist‐provided clinical services in Latin American primary health care. Its limited representation may reflect the system‐level requirements of the service, including access to accurate medication information, clearly assigned professional responsibilities, standardised workflows, and communication across hospitals, primary care, and community pharmacies. The eligibility criteria may also have influenced this finding because programmes conducted exclusively in inpatient settings were excluded. The review cannot determine whether this limited evidence reflects restricted service availability, incomplete institutionalisation of transition‐of‐care pathways, insufficient research, or underreporting. It nevertheless identifies medication reconciliation and cross‐setting continuity as areas in which additional implementation and evaluation research is warranted.

The predominance of pharmacotherapeutic follow‐up, health education, medication review, and clinically oriented dispensing indicates that the published evidence was more frequently focused on longitudinal medication management, patient education, and chronic care support [10, 16]. Pharmacotherapeutic follow‐up was operationally distinguished from stand‐alone medication review by requiring a longitudinal care process involving repeated pharmacist–patient encounters, assessment of the patient's health conditions and overall pharmacotherapy, identification of medication‐related problems, implementation of a care plan, documented interventions, and subsequent monitoring or reassessment. The lower frequencies of medication reconciliation, health screening, management of minor ailments, and therapeutic drug monitoring should not be interpreted as evidence that these services are necessarily unavailable in practice. Instead, they indicate that these activities were less frequently represented in the retrieved literature. The seven entries that were insufficiently described for classification further demonstrate the need for clearer and more consistent reporting of service objectives, components, intensity, mode of delivery, and follow‐up procedures.

Interprofessional integration was another important area of variation. Referral‐based models accounted for 54 of the 136 integration entries, followed by isolated practice (35), collaborative models (20), and integrated or transdisciplinary models (16). Integration model was not reported in 11 entries. Thus, the documented evidence more frequently described services based on referrals, occasional communication, or pharmacist practice without reported interaction with the broader healthcare team than models involving shared decisions or joint care. Interprofessional integration may facilitate the translation of pharmacists' assessments into clinical decisions through case discussion, recommendation acceptance, shared documentation, feedback pathways, and longitudinal monitoring [17]. In a retrospective hospital‐based study in which clinical pharmacists were members of interdisciplinary teams, pharmacist‐led medication review was followed by reductions in the number of medications, potentially inappropriate medications, and clinically relevant drug–drug interactions, together with improved adherence to treatment guidelines [39]. However, the uncontrolled retrospective design and hospital setting preclude causal attribution and limit extrapolation to primary health care. Participation in multidisciplinary ward rounds was not separately charted and could not be quantified because the review excluded inpatient‐only studies. The observed integration patterns should therefore be interpreted as characteristics of how services were organised and reported, rather than direct measures of the quality or effectiveness of interprofessional collaboration.

Outcome reporting was similarly uneven. Service, process, or management indicators accounted for 87 of the 182 study–outcome classifications, compared with 43 humanistic, 41 clinical, and six economic classifications; five classifications lacked a clearly identifiable outcome domain. The predominance of operational indicators is consistent with a literature substantially concerned with service establishment, activity measurement, workflow, and implementation. However, it limits the capacity of the available evidence to inform decisions regarding patient benefit, comparative value, economic sustainability, and scale‐up [28]. Systematic reviews conducted in hospital and primary care settings have reported improvements in selected clinical and prescribing outcomes, while indicating uncertainty regarding several patient‐important outcomes, medical costs, and cost‐effectiveness [7, 8]. The present review was not designed to determine whether the Latin American services produced comparable effects. The heterogeneity of interventions, populations, study designs, outcome definitions, and assessment periods also precludes causal or comparative‐effectiveness conclusions.

The reported study‐conduct or data‐collection period could be standardised for 100 studies and had a median duration of 12 months. This estimate must not be interpreted as intervention duration or longitudinal follow‐up because the reported period could include recruitment, intervention delivery, outcome assessment, or other data‐collection activities. Explicit outcome‐assessment timing was identifiable in only six studies, and only four reported assessments at 12 months or longer. Moreover, no charted study clearly distinguished a post‐intervention follow‐up period conducted after the pharmacist‐provided service had ended. The available literature therefore provides insufficient evidence to determine whether clinical, humanistic, economic, or service‐related outcomes persist after active service delivery. Longer study‐conduct periods alone should not be interpreted as evidence of sustained effectiveness.

Equity‐related reporting was limited. Only 26 studies (19.4%) reported at least one equity‐related variable, and these variables were infrequently incorporated into stratified analyses, adjusted models, subgroup descriptions, or interpretation of findings. This limitation is particularly relevant because primary health care in Latin America operates within contexts marked by social, territorial, and economic inequalities [3]. Without systematic reporting and analysis of equity‐related dimensions, the literature cannot adequately determine who is reached by pharmacist‐provided services, whether access or outcomes differ across population groups, or whether service models respond to unequal conditions of vulnerability and healthcare access. More consistent use of equity‐oriented frameworks in study design, analysis, and reporting would strengthen the policy relevance of future evaluations [30].

The findings on barriers and facilitators further demonstrate the context‐dependent nature of implementation. Sixty‐nine studies (51.5%) reported at least one barrier, most frequently methodological or evaluation limitations, organisational or structural constraints, patient‐related difficulties, and interprofessional or institutional barriers. Forty‐one studies (30.6%) reported at least one facilitator, particularly organisational arrangements, workforce preparation, interprofessional collaboration, patient or community engagement, and structured methodological tools. Because these categories were non‐mutually exclusive, individual studies could contribute to more than one category. Implementation therefore appears to depend not only on pharmacists' knowledge or motivation but also on staffing, infrastructure, workflow, access to clinical information, documentation systems, communication with other professionals, patient engagement, institutional recognition, and supportive policy and financing arrangements. This multidimensional pattern is consistent with implementation literature showing that organisational, professional, and contextual determinants interact in the incorporation of healthcare practices [12].

This review has several strengths. It was based on a prospectively registered protocol, followed methodological guidance for scoping reviews, used independent duplicate screening and data charting, and searched both indexed and grey literature sources. Including theses and dissertations broadened the mapping of locally generated service evaluations and implementation research that might otherwise have been missed; 18 of the 134 included studies (13.4%) were identified through thesis and dissertation repositories. Nevertheless, these sources may not have undergone journal peer review and may differ in methodological rigour, reporting completeness, and editorial oversight. All evidence sources were assessed using the same eligibility and data‐charting procedures, but no formal critical appraisal or assessment of certainty was undertaken. Accordingly, evidence density should not be interpreted as evidence strength or confirmation of service effectiveness. Additional limitations include heterogeneity in service terminology, incomplete reporting of intervention components and integration mechanisms, and the analytical harmonisation required to classify diverse services and outcomes. The review also did not undertake a formal comparative analysis of national policies, regulations, workforce structures, or reimbursement systems. Finally, the scoping‐review design and inclusion of descriptive, qualitative, and non‐comparative evidence were appropriate for mapping the field but do not support pooled‐effect estimates, causal attribution, or comparative‐effectiveness conclusions [18, 19].

The findings support several priorities for research and policy. Future studies should provide operationally explicit service definitions, distinguish medication review from longitudinal pharmacotherapeutic follow‐up, describe the intensity and duration of interventions, and separately report outcome‐assessment timing and post‐intervention follow‐up. Greater use of patient‐relevant, economic, implementation, and equity‐related outcomes would improve the usefulness of the evidence for health‐system decision‐making. Evaluations should also describe referral pathways, access to shared records, communication with prescribers, decision‐making responsibilities, and other components of interprofessional integration. Adherence to reporting standards may improve transparency and comparability across settings [15], while international guidance can assist in situating locally defined services within broader pharmacy‐practice functions [27]. At the policy level, sustainable incorporation of pharmacist‐provided clinical services is likely to require coordinated regulation, financing, workforce preparation, information infrastructure, monitoring, and interprofessional governance. More focused systematic reviews may become appropriate when sufficiently comparable interventions, populations, and outcomes are available. Until then, the present map indicates an expanding but geographically and methodologically uneven evidence base, with important gaps in economic evaluation, equity analysis, cross‐setting continuity, long‐term outcome assessment, and integrated models of care.

## 5 Conclusions

This review mapped a diverse range of pharmacist‐provided clinical services in primary health care across six Latin American countries, delivered mainly in primary health care units and community pharmacies linked to primary care. Most studies examined services delivered in Brazil, with substantial variation in terminology, service configuration, team integration, and outcome reporting; referral‐based and organisationally isolated models were frequently reported. Limited economic and equity assessments indicate the need for standardised, context‐sensitive evaluations to guide the equitable integration and sustainability of these services across the region.

## Author Contributions

Yasmin Silva Barrozo and Beatriz Freires Nascimento contributed equally to investigation, data curation, formal analysis, visualisation, and preparation of the original draft. Vanessa Souza Mendonça developed and executed the search strategy. GCM, Camila Campos Valerio, Livia Ribeiro Gomes, Fernanda Torres, Elisangela Costa Lima, Cesar Antunes Teixeira, and Luis Phillipe Nagem Lopes contributed to investigation, data curation, and critical revision. Alice Ramos‐Silva contributed to conceptualisation, methodology, supervision, validation, and review and editing. All authors approved the final manuscript.

## Funding

The authors have nothing to report.

## Ethics Statement

Not applicable. This review synthesised published and publicly available evidence and did not involve direct participation by humans or animals. The protocol was prospectively registered in the Open Science Framework (registration ID b2ec8) and subsequently published. The review is reported in accordance with PRISMA‐ScR; the completed checklist is supplied with the submission.

## Consent

The authors have nothing to report.

## Conflicts of Interest

The authors declare no conflicts of interest.

## Supporting information


Supporting File 1

Supporting File 2

Supporting File 3

Supporting File 4

Supporting File 5

## Data Availability

All charted data used in the synthesis are provided in Supporting Material.

## References

[1] A. Shirjang , L. Doshmangir , M. Bazyar , and V. S. Gordeev , “Primary Health Care Reforms: Ascoping Review,” Primary Health Care Research & Development 26 (2025): e74, 10.1017/S1463423625000271. PMC12455359 40820273

[2] J. P. Telles , F. Tuon , A. Cunha , and J. L. L. Rocha , “The Burden of Limited Resources in Latin America on Healthcare Quality and Management,” Lancet Regional Health. Americas 43 (2025): 101014, 10.1016/j.lana.2025.101014. PMC11875181 40034840

[3] J. Roberti , H. H. Leslie , S. V. Doubova , et al., “Inequalities in Health System Coverage and Quality: A Cross‐Sectional Survey of Four Latin American Countries,” Lancet Global Health 12, no. 1 (2024): e145–e155, 10.1016/S2214-109X(23)00488-6. PMC10716623 38096887

[4] L. Abramo , S. Cecchini , and H. Ullmann , “Enfrentar las desigualdades en salud en América Latina: el rol de la protección social,” Ciência & Saúde Coletiva 25, no. 5 (2020): 1587–1598, 10.1590/1413-81232020255.32802019. 32402029

[5] E. Zavaleta‐Monestel and S. Arguedas‐Chacón , “Reconceptualizing Access: Advancing Pharmaceutical Equity for Health System Resilience in Central and South America,” Lancet Regional Health – Americas 53 (2025): 101302, 10.1016/j.lana.2025.101302. PMC12662073 41321728

[6] R. Newham , N. Weir , A. Ferguson , and M. Bennie , “Identifying the Important Outcomes to Measure for Pharmacy‐Led Clinical Services Within Primary Care: A Nominal Group Technique Approach,” Research in Social and Administrative Pharmacy 19, no. 3 (2023): 468–476, 10.1016/j.sapharm.2022.11.003. 36404258

[7] G. Lin , R. Huang , J. Zhang , G. Li , L. Chen , and X. Xi , “Clinical and Economic Outcomes of Hospital Pharmaceutical Care: A Systematic Review and Meta‐Analysis,” BMC Health Services Research 20, no. 1 (2020): 487, 10.1186/s12913-020-05346-8. PMC7268541 32487066

[8] A. Croke , K. Cardwell , B. Clyne , F. Moriarty , L. McCullagh , and S. M. Smith , “The Effectiveness and Cost of Integrating Pharmacists Within General Practice to Optimize Prescribing and Health Outcomes in Primary Care Patients With Polypharmacy: A Systematic Review,” BMC Primary Care 24, no. 1 (2023): 41, 10.1186/s12875-022-01952-z. PMC9901090 36747132

[9] F. Fernandez‐Llimos and T. M. Salgado , “Standardization of Pharmacy Practice Terminology and the Medical Subject Headings (MeSH),” Research in Social and Administrative Pharmacy 17, no. 4 (2021): 819–820, 10.1016/j.sapharm.2020.07.005. 32763087

[10] J. P. V. Paolinelli , T. de Alencar , K. S. S. Rocha , M. L. Pereira , and G. A. dos Santos Júnior , “Implementation of Clinical Pharmacy Services in Primary Health Care: A Scoping Review,” Journal of Evaluation in Clinical Practice 31, no. 6 (2025): e70285, 10.1111/jep.70285. PMC12462563 40996324

[11] C. Vargas López and D. Chavez Gallegos , “Pharmacy Practice in Latin America: A Review of Published Literature, 2017–2021,” Drugs in Context 11 (2022): 1–7, 2022‐3‐4, 10.7573/dic.2022-3-4. PMC9281972 35912000

[12] P. Dunbar , L. M. Keyes , and J. P. Browne , “Determinants of Regulatory Compliance in Health and Social Care Services: A Systematic Review Using the Consolidated Framework for Implementation Research,” PLoS One 18, no. 4 (2023): e0278007, 10.1371/journal.pone.0278007. PMC10101495 37053186

[13] M. Stuhec , “Medication Reconciliation and Seamless Care Led by Clinical Pharmacists in Slovenia: A National Reimbursed Program Ensuring Safe and Effective Transition of Care,” International Journal of Clinical Pharmacy 47, no. 2 (2025): 239–246, 10.1007/s11096-024-01840-9. 39644375

[14] M. Stuhec and B. Batinic , “Clinical Pharmacist Interventions in the Transition of Care in a Mental Health Hospital: Case Reports Focused on the Medication Reconciliation Process,” Frontiers in Psychiatry 14 (2023): 1263464, 10.3389/fpsyt.2023.1263464. PMC10777203 38205081

[15] F. Fernandez‐Llimos , S. Desselle , D. Stewart , et al., “Improving the Quality of Publications in and Advancing the Paradigms of Clinical and Social Pharmacy Practice Research: The Granada Statements,” Research in Social and Administrative Pharmacy 19, no. 5 (2023): 830–835, 10.1016/j.sapharm.2023.01.007. 36804321

[16] L. Bader , Z. Kusynová , and C. Duggan , “FIP Perspectives: Realising Global Patient Safety Goals Requires an Integrated Approach With Pharmacy at the Core,” Research in Social and Administrative Pharmacy 15, no. 7 (2019): 815–817, 10.1016/j.sapharm.2019.02.012. 30846369

[17] A. Urionagüena , C. Piquer‐Martinez , M. Á. Gastelurrutia , et al., “Community Pharmacy and Primary Health Care‐Types of Integration and Their Applicability: A Narrative Review,” Research in Social and Administrative Pharmacy 19, no. 3 (2023): 414–431, 10.1016/j.sapharm.2022.10.007. 36336618

[18] Z. Munn , M. D. J. Peters , C. Stern , C. Tufanaru , A. McArthur , and E. Aromataris , “Systematic Review or Scoping Review? Guidance for Authors When Choosing Between a Systematic or Scoping Review Approach,” BMC Medical Research Methodology 18, no. 1 (2018): 143, 10.1186/s12874-018-0611-x. PMC6245623 30453902

[19] M. D. J. Peters , C. Marnie , A. C. Tricco , et al., “Updated Methodological Guidance for the Conduct of Scoping Reviews,” JBI Evidence Synthesis 18, no. 10 (2020): 2119–2126, 10.11124/JBIES-20-00167. 33038124

[20] A. C. Tricco , E. Lillie , W. Zarin , et al., “PRISMA Extension for Scoping Reviews (PRISMA‐ScR): Checklist and Explanation,” Annals of Internal Medicine 169, no. 7 (2018): 467–473, 10.7326/M18-0850. 30178033

[21] B. F. Nascimento , Y. S. Barrozo , V. S. Mendonça , L. P. Lopes , and A. Ramos‐Silva , “Pharmaceutical Clinical Services in Primary Health Care in Latin America: Scoping Review Protocol,” Journal of Hospital Pharmacy and Health Services 17, no. 2 (2026): 1–5, 10.30968/jhphs.2026.172.1459.

[22] World Health Organization . United Nations Children's Fund. Declaration of Astana. World Health Organization; 2018, accessed August 13, 2026, https://www.who.int/publications/i/item/WHO-HIS-SDS-2018.61.

[23] F. White , “Primary Health Care and Public Health: Foundations of Universal Health Systems,” Medical Principles and Practice 24, no. 2 (2015): 103–116, 10.1159/000370197. PMC5588212 25591411

[24] M. L. Bastos , D. Menzies , T. Hone , K. Dehghani , and A. Trajman , “The Impact of the Brazilian Family Health on Selected Primary Care Sensitive Conditions: A Systematic Review,” PLoS One 12, no. 8 (2017): e0182336, 10.1371/journal.pone.0182336. PMC5546674 28786997

[25] N. Hatam , M. Askarian , E. Taherifard , et al., “Exploring Patient‐Centered Care Delivery in Outpatient Settings for Older Adults: A Scoping Review and Recommendations for Implementation in Countries With Low and Middle Income,” BMC Geriatrics 25 (2025): 940, 10.1186/s12877-025-06643-9. PMC12639653 41272495

[26] Conselho Federal de Farmácia . Serviços farmacêuticos diretamente destinados ao paciente, à família e à comunidade: contextualização e arcabouço conceitual. Conselho Federal de Farmácia; 2016.

[27] World Health Organization . International Pharmaceutical Federation. Joint FIP/WHO Guidelines on Good Pharmacy Practice: Standards for Quality of Pharmacy Services. In: WHO Expert Committee on Specifications for Pharmaceutical Preparations: Forty‐ Fifth Report. WHO Technical Report Series No. 961. World Health Organization; 2011:310‐323.

[28] N. M. Weir , K. Preston , R. Newham , and M. Bennie , “Development of a Primary Care Pharmacy Outcomes Framework: An Umbrella Literature Review,” Research in Social and Administrative Pharmacy 18, no. 5 (2022): 2757–2777, 10.1016/j.sapharm.2021.07.010. 34353755

[29] C. M. Kozma , C. E. Reeder , and R. M. Schulz , “Economic, Clinical, and Humanistic Outcomes: A Planning Model for Pharmacoeconomic Research,” Clinical Therapeutics 15, no. 6 (1993): 1121–1132; discussion 1120. 8111809

[30] E. L. Karran , A. G. Cashin , T. Barker , et al., “Using PROGRESS‐Plus to Identify Current Approaches to the Collection and Reporting of Equity‐Relevant Data: A Scoping Review,” Journal of Clinical Epidemiology 163 (2023): 70–78, 10.1016/j.jclinepi.2023.09.017. 37802205

[31] Brasil. Ministério da Saúde . Portaria n° 1.214, de 13 de junho de 2012. Institui o Programa Nacional de Qualificação da Assistência Farmacêutica no âmbito do Sistema Único de Saúde (QUALIFAR‐SUS), accessed August 15, 2026, https://www.gov.br/saude/pt-br/acesso-a-informacao/gestao-do-sus/articulacao-interfederativa/cit/pautas-de-reunioes-e-resumos/2012/abril/2-b-apresentacao-qualifar_sus_cit.pdf.

[32] Resolução n° 585, de 29 de agosto de 2013. Regulamenta as atribuições clínicas do farmacêutico e dá outras providências Conselho Federal de Farmácia, 2026, https://www.cff.org.br/userfiles/file/resolucoes/585.pdf.

[33] Brasil. Lei n . 13.021, de 8 de agosto de 2014. Dispõe sobre o exercício e a fiscalização das atividades farmacêuticas, accessed August 15, 2026, https://www.planalto.gov.br/ccivil_03/_ato2011-2014/2014/lei/l13021.htm.

[34] A. L. M. Simone , T. F. Saldanha , and D. O. Melo , “Cuidado farmacêutico no Sistema Único de Saúde: perfil e financiamento federal dos serviços,” Jornal de Assistência Farmacêutica e Farmacoeconomia 10, no. 3 (2025): e00249, 10.22563/2525-7323.2025.v10.e00249.

[35] O. Rose , C. Egel , J. Pachmayr , and S. Clemens , “Pharmacist‐Led Prescribing in Austria: A Mixed‐Methods Study on Clinical Readiness and Legal Frameworks,” Pharmacy (Basel, Switzerland) 13, no. 5 (2025): 130, 10.3390/pharmacy13050130. PMC12452385 40981250

[36] M. Stuhec , “Clinical Pharmacist Consultant in Primary Care Settings in Slovenia Focused on Elderly Patients on Polypharmacy: Successful National Program From Development to Reimbursement,” International Journal of Clinical Pharmacy 43, no. 6 (2021): 1722–1727, 10.1007/s11096-021-01306-2. 34228266

[37] M. Stuhec , A. Kovacic , M. Korpar , et al., “Clinical Pharmacist Prescriber in Primary Care in Slovenia: Prospective Non‐Randomised Interventional Study Focused on Clinical Outcomes and Quality of Life,” Frontiers in Pharmacology 16 (2025): 1690480, 10.3389/fphar.2025.1690480. PMC12477041 41031168

[38] M. Stuhec , A. Kovacic , M. Korpar , et al., “Pharmacist Prescriber Implementation in the Experiences of General Practitioners, Pharmacist Prescribers and Patients: Qualitative Study Based on Pilot Trial in Slovenia,” Frontiers in Pharmacology 16 (2025): 1712595, 10.3389/fphar.2025.1712595. PMC12646872 41311844

[39] M. Stuhec , N. Svetanic , T. Vovk , A. Gorjan Gazdag , Z. Cuk , and B. Batinic , “Clinical Pharmacist Interventions in Medication Review for Medication Optimization in Older Hospitalized Adults With Mental Disorders and Somatic Comorbidities: Evidence From Retrospective Study,” Frontiers in Pharmacology 16 (2025): 1667584, 10.3389/fphar.2025.1667584. PMC12433881 40959457

