# Supplementary material for: Clinical Services Provided by Pharmacists in Primary Health Care in Latin America: A Scoping Review
Source: J Eval Clin Pract. 2026 Nov 4;32(8):e70628. doi: 10.1111/jep.70628 (PMC13734460; doi:10.1111/jep.70628)
Supplement: Supplementary file 1 [file JEP-32-0-s001.docx]

**Supplementary Material 4**

Detailed classification of barriers to implementation reported in the included studies

This supplement summarizes the barriers explicitly reported in the included studies and groups them into thematic categories for presentation.

**Included studies reporting barriers: 69 of 134 (51.5%).**

**Table S4.1. Summary of barrier themes**

| **Barrier theme** | **Definition** | **Studies, n** | **Studies, %** |
| --- | --- | --- | --- |
| Methodological and evaluation limitations | Small samples, short follow-up, missing data, indirect measures, limited generalizability, and other design constraints. | 35 | 50.7 |
| Organizational and structural constraints | Time, workload, infrastructure, materials, documentation systems, installation costs, and service-organization constraints. | 20 | 29.0 |
| Patient-related barriers | Low adherence, limited health literacy or schooling, socioeconomic constraints, missed follow-up, and difficulty accessing care. | 18 | 26.1 |
| Interprofessional and institutional barriers | Weak team integration, limited autonomy, low institutional support, poor prescriber communication, and lack of incentives. | 17 | 24.6 |
| Workforce and training barriers | Shortage of pharmacists or staff, limited experience, insufficient training, and low diffusion of service-related knowledge. | 10 | 14.5 |
| Contextual and external barriers | Pandemic disruption, geographical distance, cultural or intersectoral factors, and broader health-system context. | 6 | 8.7 |

*Note: Counts exceed 69 because one study could report barriers in more than one thematic category.*

**Table S4.2. Study-level details of reported barriers**

*Studies are listed alphabetically by first author/year as recorded in the extraction file.*

| **Study ID** | **Author, year** | **Country** | **Thematic category or categories** | **Reported barrier(s)** |
| --- | --- | --- | --- | --- |
| 284 | Ahumada-Canale,2021 | Chile | Organizational and structural constraints; Workforce and training barriers; Methodological and evaluation limitations | Aumentos de custo inicial para treinemento de equipe e instalação do serviço \|\| hospitalizações parcialmente contabilizadas;uso de um catálogo introduziu incertezas |
| OIASTER166 | Ahumada-Canalea.A, 2021 | Chile | Organizational and structural constraints; Interprofessional and institutional barriers | barreira econômica, farmacêutico não tem autonomia de prescrição independente, necessitando avaliação do médico |
| OIASTER241 | Andrade. R, 2005 | Brasil | Patient-related barriers | baixa escolaridade e dificuldade decompreensão, falta de conhecimento da propria doença |
| 35 | Antunes,2021 | Brasil | Methodological and evaluation limitations | A técnica de entrevista, devido ao tempo envolvido e à profundidade da pesquisa, limita o número de indivíduos a serem pesquisados |
| 24 | Armando,2005 | Argentina | Workforce and training barriers | Baixa quantidade de farmacêuticos |
| OATD03 | BARROS, 2018 | Brasil | Organizational and structural constraints; Interprofessional and institutional barriers | Falta de recursos, infraestrutura precária e baixo reconhecimento profissional. |
| CAPES18 | Bandeira, 2023 | Brasil | Organizational and structural constraints; Interprofessional and institutional barriers | As principais dificuldades estão relacionadas à falta de tempo, estrutura e incentivos. |
| 140 | Barros,2022 | Brasil | Methodological and evaluation limitations | Não utilização de instrumento validado para coleta de dados; |
| 22 | Bonadiman,2018 | Brasil | Workforce and training barriers | Baixo número de farmacêuticos atuantes em farmácias básicas, sendo baixa a verificação do nível de conhecimento; |
| 41 | Canguçu, 2019 | Brasil | Methodological and evaluation limitations | Não foi possível diferenciar qual intervenção foi mais eficaz em termos de desfechos, apresentando, portanto, efeito equivalente entre as duas intervenções. \|\| não foi possível diferenciar qual intervenção foi mais eficaz em termos de desfechos, apresentando, portanto, efeito equivalente entre as duas intervenções. |
| 225 | Cani,2015 | Brasil | Methodological and evaluation limitations | tamanho reduzido da população; curto período de acompanhamento |
| 216 | Cazarim,2016 | Brasil | Methodological and evaluation limitations | falta de dados de muitos pacientes; ausência de grupo controle |
| 6 | Ceballo,2021 | Colombia | Methodological and evaluation limitations | Visitas não foram gravadas para minimicar vieses associados às informações coletadas, visto que eram pacientes simulado |
| 175 | Ceballos,2020 | Colombia | Methodological and evaluation limitations | paciente simulado; visitas não gravadas; |
| 278 | Cerqueira-Santos,2022 | Brasil | Methodological and evaluation limitations | resultado generalizado a região; |
| 62 | Correr,2011 | Brasil | Methodological and evaluation limitations | Uso de farmácias controles e intervenção distintas; utilizaçãoi de potno de corte de HbA1c de 8% |
| OIASTER133 | Couto.B, 2006 | Brasil | Patient-related barriers | baixa escolaridade e o baixo poder aquisitivo são fatores que dificultam o entendimento das orientações e o cumprimento da terapia |
| 152 | Dias,2022 | Brasil | Workforce and training barriers | curto tempo de experiência dos farmacêuticos no atendimento clinico |
| OIASTER108 | Divaldo P, 2005 | Brasil | Interprofessional and institutional barriers | Falta de integração com a equipe médica |
| OIASTER80 | Divaldo P, 2007 | Brasil | Organizational and structural constraints; Patient-related barriers | resistencia a mudança de comportamento (pelos idosos), falta de espaço fisico adequado, baixa escolaridade dos pacientes |
| 11 | Dosea,2017 | Brasil | Organizational and structural constraints; Interprofessional and institutional barriers; Contextual and external barriers | Distância entre as cidades vizinhas, difucultando continuidade do atendimento de alguns pacientes; Relacionamento com médicos; Estrutura Física da Farmácia Aumento da demanda de serviço |
| 27 | Durigon et al., 2023 | Brasil | Interprofessional and institutional barriers; Workforce and training barriers | URM ainda é uma pauta pouco abordada nas reuniões de equipe e em ações de EPS. Pouca difusão do conhecimento sobre as políticas de URM entre os profissionais. |
| 113 | Esandi,2024 | Argentina | Organizational and structural constraints; Patient-related barriers; Workforce and training barriers; Contextual and external barriers | Período de pandemia do COVID, gerando baixo desempenho do programa; barreiras relacionadas à tecnologia, adequação do paciente, caracteristicas da equipe de implementação e fatores organizacionais |
| 224 | Firmino, 2015 | Brasil | Interprofessional and institutional barriers; Patient-related barriers | Relutância da população local em compreender os benefícios de um serviço orientado sobre farmacoterapia; falta de adesão pelos outros profissionais; alguns pacientes não compareceram no LACT para coletar sangue |
| 150 | Gome,2022 | Brasil | Methodological and evaluation limitations | avaliação de adesão por natureza observacional; |
| 249 | Gualda,2009 | Argentina | Interprofessional and institutional barriers; Patient-related barriers | Baixa adesão a prescrição com nome genérico dos medicamentos. Necessidade de adaptar as orientações ao nível socio-cultural dos pacientes. \|\| Necessidade de adapatar as orientações com relação ao nível socio-cultural dos pacientes. \|\| Por terem sidos feitas viagens de cmapo, os médicos que iam nessas viagens eram diferentes. O que dificultou a unificação dos critérios de prescrição |
| 263 | Hass,2006 | Brasil | Methodological and evaluation limitations | população pequena;curto período de acompanhamento; |
| 260 | Júnior,2007 | Brasil | Workforce and training barriers; Methodological and evaluation limitations; Contextual and external barriers | Experiência e formação do farmacêutico pesquisador em Assistência Farmacêutica; pequeno número de paciente; diferença no sistema de saúde; diferentes abordagens para formação dos profissionais. |
| 133 | Laborne-e-Valle,2022 | Brasil | Organizational and structural constraints; Methodological and evaluation limitations | incidência de cessação de tabagismo pode ter sido superestimada, visto que a absitinência não foi determinada por marcadores bioquímicos; reccurso laboratorial limitado para testagem de cessação tabágica; não foi avaliada a cessação após 12 meses; |
| 119 | Lara-Júnior, 2022 | Brasil | Organizational and structural constraints | Documentação deficiente no sistema de gestão de serviços farmacêuticos |
| 114 | Lira-Meriguete,2023 | Brasil | Methodological and evaluation limitations | curto período de acompanhamento; |
| 8 | Martins et al., 2025 | Brasil | Organizational and structural constraints; Patient-related barriers | descontentamento dos pacientes pelo não recebimento das tiras reagentes por meio do serviço público, |
| 241 | Martins,2013 | Brasil | Patient-related barriers | Dificuldade dos pacientes em marcar consultas com médicos que houve encaminhamento an intervenção, impactando resultado; |
| OIASTER59,60,61 | Martínez-Mardones F, 2023 | Chile | Organizational and structural constraints; Contextual and external barriers | falta de tempo, pandemia de COVID19 |
| 132 | Mauricio,2016 | Brasil | Methodological and evaluation limitations | Baixo tamanho da amostra |
| 39 | Melo et al., 2016 | Brasil | Interprofessional and institutional barriers; Patient-related barriers | Falta de comunicação entre profissionais de saúde, principalmente quando o paciente é atendido nos setores público e privado. Pacientes com regimes terapêuticos complexos dificultando o uso racional de medicamentos. \|\| prescrições de multiplos profissionais levando à duplicidade terapêutica. Dificuldade de contato com os profissionais médicos. |
| 120 | Melo, 2017 | Brasil | Interprofessional and institutional barriers | integração do farmacêutico com a equipe miltidisciplinar; |
| 100 | Mendes,2020 | Brasil | Organizational and structural constraints | pesquisador ficar em campo por tempo indeterminado. |
| 61 | Mino-León, 2012 | México | Methodological and evaluation limitations | Curta duração da intervenção famacoterapêutica \|\| Estudo transversal (não estabelece causa), Amostra escolhida por conveniência, sub-representação de populações mais vulneráveis |
| 73 | Mino-León, 2014 | México | Methodological and evaluation limitations | Falta de randomização devido condições reais das clínicas; estudo com 3 tipos de pacientes (hipertensão, diabetico e ambos); tempo curto dos follow-up |
| 54 | Miranda et al., 2022 | Equador | Interprofessional and institutional barriers; Patient-related barriers | Falta de aceitação das intervenções realizados a nível do prescritor, falta de comunicação com o prescritor. Dificulta compreensão dos pacientes sobre o uso correto de medicamentos. |
| 192 | Molino,2017 | Brasil | Organizational and structural constraints; Methodological and evaluation limitations | Alguns prontuários não puderam ser avaliados devido agenda do médico; falta de informação para determinar % de PRM; |
| OIASTER152 | Neto P, 2011 | Brasil | Interprofessional and institutional barriers; Patient-related barriers; Contextual and external barriers | dificuldade de comunicação intersetorial, fatores culturais e conhecimento dos pacientes |
| 60 | Neto,2012 | Brasil | Methodological and evaluation limitations | Fontes disponíveis para analisar interações medicamentosas são muitas e com diferentes classificações de risco/importância que não são consesuais, o que pode ter gerado o risco de algumas interações terem sido subestimados. baixa quantidade de UBd envolvida |
| 108 | Neves,2024 | Brasil | Methodological and evaluation limitations; Contextual and external barriers | Resultados não podem ser representativos para todos os municípios de Minas Gerais ou do Brasil, devido difereneças sociais, econômicas e culturais |
| 245 | Obreli-Neto,2011 | Brasil | Methodological and evaluation limitations | ausência de método padrão-ouro para medir adesão; desenho do estudo não permitir análise individual de cada componente da assistência farmacêutica; estudo realizado em apenas uma UBS, levando a generalização |
| 20 | Obreli-Neto,2013 | Brasil | Methodological and evaluation limitations | Efeito Hawthorne Apenas participantes de dua sminirregiões, sendo os resultados generalizados |
| 48 | Oenning et al., 2011 | Brasil | Organizational and structural constraints; Patient-related barriers | A maioria dos entrevistados não possuia bom nível de informação para a administração de medica- mentos. Necessidade de aprimorar os meios de fornecer a informação ao paciente, a organização do tempo dedicado à orientação pelo prescritor e atuação do farmacêutico na dispensação. |
| 50 | Oliveira,2017 | Brasil | Patient-related barriers; Workforce and training barriers | Reduzida adesão dos farmacêuticos treinados com o POP para a continuiadade do projeto ( coleta de informações da dispensação). Baixo nível de conhecimento dos usuários contribuem para a automedicação com antibióticos. \|\| Tempo maior para execução do projeto; pequeno número de farmacêuticos envolvidos |
| 173 | Packeiser,2020 | Brasil | Patient-related barriers; Methodological and evaluation limitations | dificuldade dos participantes em se integrar à situação simulada e em enxerrgar a atividade como uma situação real do dia a dia; falta de adesão, pois muitos alunos realizatam apenas a atividade obrigatória do curso; vies de observador, pois as gravações foram observadas por um único pesquisador |
| 45 | Parody,2018 | Colombia | Patient-related barriers; Methodological and evaluation limitations | Falta de acompanhamento dos pacientes e impossibilidade de avaliar 100% dos pacientes com artrite; vies de resposta pelos pacientes em relação a adesão; |
| BDTD38 | Pereira,2019 | Brasil | Interprofessional and institutional barriers | Submissão do projetos de pesquisa ao CEP; UBS que não demonstraram interesse por medo/desconfiança |
| 15 | Ramos et al., 2023 | Brasil | Organizational and structural constraints; Interprofessional and institutional barriers; Patient-related barriers; Workforce and training barriers | número insuficiente de recursos humanos; falta de estímulo institucional à prescrição; e fatores individuais. |
| 83 | Rodrigues,2023 | Brasil | Methodological and evaluation limitations | curto tempo de intervenção; possíveis frequência de omissaõ; |
| OATD44 | Saldivia, 2020 | Chile | Patient-related barriers; Methodological and evaluation limitations | Impossibilidade de extrapolar os resultados para população com diabetes tipo II, que é baixa adesão à terapia medicamentosa |
| OATD21 | Santos, 2018 | Brasil | Organizational and structural constraints | Inserção de um modelo de serviço muito novo na atenção primarioa do sistema público |
| 84 | Santos,2023 | Brasil | Interprofessional and institutional barriers; Workforce and training barriers | Pouco farmacêutico e pacientes; falta de incentivo pelo municipio; |
| OATD22 | Silva, 2024 | Brasil | Methodological and evaluation limitations | Tempo de acompanhamento curto, medidas em casa feitas pelos próprios pacientes e estudo em um único centro |
| 154 | Silva,2015 | Brasil | Interprofessional and institutional barriers | Conscientizar a equipe de hanseníase a introduzir o farmacêutico no cuidado à pacientes que estavam inicinado o tratamento; |
| 190 | Silva,2017 | Brasil | Methodological and evaluation limitations | tempo de campo e número de participantes; pouco número de farmacêuticos entrevistados |
| CAPES05 | Solbiati, 2024 | Brasil | Organizational and structural constraints | Sobrecarga de atividades administrativas |
| 12 | Souza et al., 2025 | Brasil | Organizational and structural constraints; Methodological and evaluation limitations | Necessidade de validar os meteriais produzidos, Falta de instrumentos como impressoras e equipamento de gravação. |
| 37 | Souza,2020 | Brasil | Methodological and evaluation limitations | Falta de dados de algumas variáveis independentes, como etnia/cor, altura, nível de escolaridade, além de peso, índice de massa corporal e circunferência da cintura |
| 44 | Sánchez-Guerra, 2018 | México | Methodological and evaluation limitations | Questionário de adesão suscetível ao erro por conta dos pacientes; não realização de testes sobre o estado de órgão como coração e rins; \|\| Questionário de adesão suscetível ao erro por conta dos pacientes; não realização de testes sobre o estado de órgão como coração e rins; amostra pequena de pacientes; |
| 16 | Traverso,2007 | Argentina | Methodological and evaluation limitations | Farmacêuticos podem ter selecionado pacientes; Questionário é geograficamento limitado por ser espanhol |
| OATD43 | Trindade, 2015 | Brasil | Organizational and structural constraints; Interprofessional and institutional barriers; Patient-related barriers | Baixa escolaridade, não adesão (esquecimento/recusa), falha na comunicação com médicos e falta de medicamentos |
| 21 | Turner, 2003 | México | Methodological and evaluation limitations | Uso de 3 casos para avaliar a competência da prática farmacêutica Estudo ter ocorrido apenas na Cidade do México |
| 14 | Vinholes et al., 2009 | Brasil | Organizational and structural constraints | espaço físico inadequado, limitação didática dos diapositivos(slides) |
| 155 | silva,2021 | Brasil | Methodological and evaluation limitations | baixo número de entrevistados e avaliação indireta dos serviços farmacêuticos |
