# Supplementary material for: Clinical Services Provided by Pharmacists in Primary Health Care in Latin America: A Scoping Review
Source: J Eval Clin Pract. 2026 Nov 4;32(8):e70628. doi: 10.1111/jep.70628 (PMC13734460; doi:10.1111/jep.70628)
Supplement: Supplementary file 2 [file JEP-32-0-s002.docx]

**Supplementary Material 5
Study-level classification of reported facilitators to implementation, delivery, or evaluation of pharmacist-provided clinical services in primary health care in Latin America**

This supplementary material summarizes facilitators explicitly reported in the data extraction sheet. A single study could contribute to more than one thematic category. Percentages in the summary table use as denominator the studies with at least one reported facilitator (n = 41).

**Table S5.1. Summary of thematic categories of reported facilitators**

| **Thematic category** | **Studies, n** | **Percentage (%)** |
| --- | --- | --- |
| Organizational and structural facilitators | 14 | 34.1 |
| Workforce and training facilitators | 13 | 31.7 |
| Patient-related and community facilitators | 10 | 24.4 |
| Interprofessional and institutional facilitators | 10 | 24.4 |
| Methodological and evaluation facilitators | 9 | 22.0 |
| Policy, financial, and contextual facilitators | 5 | 12.2 |

**Table S5.2. Study-level facilitators and thematic classification**

| **Study** | **Reported facilitator(s)** | **Thematic category(ies)** |
| --- | --- | --- |
| Ahumada-Canalea et al., 2021 | Potential incorporation of pharmacotherapeutic follow-up into the public health system. | Organizational and structural facilitators; Policy, financial, and contextual facilitators |
| Ahumada-Canalea A., 2021 | Integration into existing programmes and intensive clinical training. | Organizational and structural facilitators; Workforce and training facilitators |
| Amando et al., 2025 | Training increased pharmacists’ knowledge, confidence, and engagement, and improved patient counselling. | Workforce and training facilitators |
| Andrade R., 2005 | Use of standardized instruments and individualized health education. | Methodological and evaluation facilitators; Patient-related and community facilitators |
| Brito et al., 2009 | Possibility of home follow-up for patients. | Organizational and structural facilitators; Patient-related and community facilitators |
| Canguçu, 2019 | Simple, low-cost technology that could be implemented in any health service. | Organizational and structural facilitators |
| Cerqueira-Santos, 2023 | Partnership between the university pharmacy social research department and the community pharmacy network under study. | Interprofessional and institutional facilitators |
| Couto B., 2006 | Community bond and presence of collective intervention activities. | Patient-related and community facilitators |
| Divaldo P., 2005 | University pharmacy structure and partnership with university extension projects. | Organizational and structural facilitators; Interprofessional and institutional facilitators |
| Divaldo P., 2007 | Verbal and non-verbal communication skills. | Workforce and training facilitators |
| Dosea, 2017 | No pressure to meet medicine sales targets and availability of a private counselling room. | Organizational and structural facilitators |
| Durigon et al., 2023 | Recognition by other professionals of the pharmacist’s role in care and of medication-related educational needs. | Interprofessional and institutional facilitators |
| Esandi, 2024 | Funding from the Argentine Ministry of Health. | Policy, financial, and contextual facilitators |
| Implementation of pharmaceutical care in a primary health care unit [author/year unavailable in extraction sheet] | Management by district pharmacies linked to the Belo Horizonte municipal network. | Organizational and structural facilitators; Interprofessional and institutional facilitators |
| Júnior, 2007 | Collaboration between the health team and the pharmacist. | Interprofessional and institutional facilitators |
| Laborne-e-Valle, 2022 | Real-world service data generated through a hybrid design supported by WHO encouragement. | Methodological and evaluation facilitators; Policy, financial, and contextual facilitators |
| Lira-Meriguete, 2023 | Funding from a state research foundation. | Policy, financial, and contextual facilitators |
| Marcelo et al., 2009 | Development of a pharmacotherapeutic guide improved communication between medical and pharmacy teams; pharmacy students supported production and distribution of educational materials. | Interprofessional and institutional facilitators; Workforce and training facilitators |
| Martins et al., 2025 | Appointment scheduling for consultations. | Organizational and structural facilitators |
| Martínez-Mardones F., 2023 | Structured primary care team and good interprofessional relationships. | Interprofessional and institutional facilitators |
| Mauricio, 2016 | Pioneering application of Beers criteria. | Methodological and evaluation facilitators |
| Melo et al., 2016 | Pharmacists were prepared to tailor counselling to patients with literacy, visual, or auditory limitations; patient acceptance of medication reconciliation. | Workforce and training facilitators; Patient-related and community facilitators |
| Miranda et al., 2022 | Good acceptance of non-pharmacological counselling and the presence of support networks that favoured adherence. | Patient-related and community facilitators |
| Neto P., 2011 | Use of pharmacotherapeutic history software, clinical dispensing protocols, and educational materials. | Methodological and evaluation facilitators; Organizational and structural facilitators; Workforce and training facilitators |
| Neto, 2012 | Access to prescription data, patient interviews, and medical records, together with long study duration. | Methodological and evaluation facilitators |
| Neto, 2013 | Access to prescription data, patient interviews, and medical records, together with long study duration. | Methodological and evaluation facilitators |
| Neves, 2024 | Financial support from FAPEMIG. | Policy, financial, and contextual facilitators |
| Obreli-Neto, 2013 | Use of the simulated patient method. | Methodological and evaluation facilitators |
| Oenning et al., 2011 | Dispensing performed primarily by the pharmacist enabled comparison of patient knowledge according to exposure to pharmacist-led dispensing. | Methodological and evaluation facilitators |
| Oliveira, 2017 | Clinical pharmacy team received technical support from UFMG. | Interprofessional and institutional facilitators; Workforce and training facilitators |
| Paulós, 2005 | Availability of a counselling area in the pharmacy. | Organizational and structural facilitators |
| Paulós, 2007 | Availability of a counselling area in the pharmacy. | Organizational and structural facilitators |
| Ramos et al., 2023 | Infrastructure, easy access to the pharmacist, and good acceptance of prescribing recommendations. | Organizational and structural facilitators; Interprofessional and institutional facilitators |
| Saldivia, 2020 | Communication between the team and patients. | Interprofessional and institutional facilitators; Patient-related and community facilitators |
| Schneidera et al., 2022 | Previous pharmacist training, geographic distribution of community pharmacies, and use of a validated FINDRISC form. | Workforce and training facilitators; Methodological and evaluation facilitators; Organizational and structural facilitators |
| Solbiati, 2024 | Structured educational support through a guide. | Workforce and training facilitators |
| Souza et al., 2025 | Prior team training and availability of materials from the municipal pharmaceutical services coordination. | Workforce and training facilitators; Organizational and structural facilitators |
| Trindade, 2015 | Adapted educational materials using pictograms. | Patient-related and community facilitators; Workforce and training facilitators |
| Trindade, 2016 | Adapted educational materials using pictograms. | Patient-related and community facilitators; Workforce and training facilitators |
| Trindade, 2017 | Adapted educational materials using pictograms. | Patient-related and community facilitators; Workforce and training facilitators |
| Vinholes et al., 2009 | Good reception of health education activities delivered by the pharmaceutical care service. | Patient-related and community facilitators |
