# Supplementary material for: Clinical Services Provided by Pharmacists in Primary Health Care in Latin America: A Scoping Review
Source: J Eval Clin Pract. 2026 Nov 4;32(8):e70628. doi: 10.1111/jep.70628 (PMC13734460; doi:10.1111/jep.70628)
Supplement: Supplementary file 3 [file JEP-32-0-s003.docx]

**Supplementary Material 2. Full-text reports excluded and reasons**

| **No.** | **Article title** | **Authors** | **Year** | **Reason for exclusion** |
| --- | --- | --- | --- | --- |
| 1 | A ATENÇÃO FARMACÊUTICA NAS FARMÁCIAS DE COMUNIDADE COMO FERRAMENTA DE ACESSO A ATENÇÃO PRIMARIA DE SAÚDE NO BRASIL | BARSAGLINI. G |  | Not a primary study design |
| 2 | Política de Assistência Farmacêutica: A questão da atenção farmacêutica no SUS | Araújo. P | 2016 | Not a primary study design |
| 3 | ACESSO A MEDICAMENTOS NA ATENÇÃO BÁSICA:RELAÇÕES COM A INSTITUCIONALIZAÇÃO DA ASSISTÊNCIA FARMACÊUTICA NO BRASIL | Barros. R | 2016 | No pharmacists involved |
| 4 | ANÁLISE DA IMPLANTAÇÃO DO PROGRAMA NACIONAL DE QUALIFICAÇÃO DA ASSISTÊNCIA FARMACÊUTICA PARA A ESTRUTURAÇÃO DOS SERVIÇOS FARMACÊUTICOS NA ATENÇÃO BÁSICA. | Rodrigues. P | 2016 | No pharmacists involved |
| 5 | AUTOMEDICAÇÃO DE TRABALHADORES NA ATENÇÃO PRIMÁRIA À SAÚDE | Andrade. S | 2018 | No pharmacists involved |
| 6 | “Monitoramento do desempenho dos serviços farmacêuticos na Atenção Primária à Saúde: buscando a qualificação da gestão” | Pereira.N | 2013 | Did not describe a clinical pharmacy service |
| 7 | PROFISSÃO FARMACÊUTICA NA ATENÇÃO BÁSICA EM SAÚDE: TERRITÓRIOS POSSÍVEIS | Loss. G; Loss.G | 2019 | No pharmacists involved |
| 8 | A PERCEPÇÃO DOS FARMACÊUTICOS COMO PROFISSIONAIS DA ATENÇÃO PRIMÁRIA DE SAÚDE NO SUS DE FORTALEZA – CE | Oliveira.A | 2008 | Did not describe a clinical pharmacy service |
| 9 | PROBLEMAS ÉTICOS VIVENCIADOS POR FARMACÊUTICOS NA ATENÇÃO BÁSICA À SAÚDE EM UM MUNICÍPIO DO SUL DO BRASIL | Molina.L | 2016 | Did not describe a clinical pharmacy service |
| 10 | ENSAIOS SOBRE ASSISTÊNCIA FARMACÊUTICA E PARTICIPAÇÃO DA COMUNIDADE NA SAÚDE NA ATENÇÃO BÁSICA | Pinto.R | 2021 | No pharmacists involved |
| 11 | ASSISTÊNCIA FARMACEÛTICA NA ATENÇÃO PRIMÁRIA DE SAÚDE NO MUNICÍPIO DE CAZENGA ANGOLA |  |  | Not conducted in a Latin American country |
| 12 | Avaliação da capacidade de gestão da Assistência Farmacêutica na Atenção Primária à Saúde nos municípios brasileiros | Faraco.E | 2021 | Did not describe a clinical pharmacy service |
| 13 | ASSISTÊNCIA FARMACÊUTICA NO CONTEXTO DA ATENÇÃO PRIMÁRIA À SAÚDE DE UM MUNICÍPIO BRASILEIRO | Guimarães.S | 2020 | Did not describe a clinical pharmacy service |
| 14 | O Acesso aos Medicamentos na Atenção Primária à Saúde em Belo Horizonte | Aquino.F | 2019 | No pharmacists involved |
| 15 | SERVIÇOS FARMACÊUTICOS CLÍNICOS DA ATENÇÃO PRIMÁRIA À SAÚDE DO DISTRITO FEDERAL | Barros.D | 2018 | Did not describe a clinical pharmacy service |
| 16 | ANÁLISE DA ATIVIDADE DE TRABALHO DO FARMACÊUTICO NA ATENÇÃO PRIMÁRIA NO DISTRITO FEDERAL, BRASIL | Barberato.L | 2017 | Did not describe a clinical pharmacy service |
| 17 | MELHORIA DA QUALIDADE DA ASSISTÊNCIA FARMACÊUTICA EM UM MUNICÍPIO DO OESTE POTIGUAR, BRASIL | Guergel.G | 2022 | Did not describe a clinical pharmacy service |
| 18 | O FARMACÊUTICO NA COMPOSIÇÃO DA FORÇA DE TRABALHO EM SAÚDE NA ATENÇÃO PRIMÁRIA DO SUS | Carvalho.M | 2016 | Not a primary study design |
| 19 | ACESSO E USO RACIONAL DE MEDICAMENTOS NA ATENÇÃO PRIMÁRIA À SAÚDE | Gerlack.L; Gerlak.L | 2016 | No pharmacists involved |
| 20 | GERENCIAMENTO DA TERAPIA MEDICAMENTOSA NA ATENÇÃO PRIMÁRIA DO MUNICÍPIO DE BELO HORIZONTE: uma autoetnografia da transformação profissional | Silva.D | 2016 | Did not describe a clinical pharmacy service |
| 21 | AVALIAÇÃO DA ASSISTÊNCIA FARMACÊUTICA NA ATENÇÃO BÁSICA DA SAÚDE NO BRASIL COM BASE NO PROGRAMA NACIONAL DE MELHORIA DO ACESSO E DA QUALIDADE DA ATENÇÃO BÁSICA (PMAQ-AB) | Junior.O | 2017 | Did not describe a clinical pharmacy service |
| 22 | “Assistência Farmacêutica na Atenção Básica e Programa Farmácia Popular do Brasil: uma análise crítica das políticas públicas de provisão de medicamentos no Brasil” | Mattos.L | 2015 | No pharmacists involved |
| 23 | Construção de um modelo de avaliação do processo de assistência farmacêutica na atenção primária – uma ênfase ao processo de cuidado | Soares.L | 2017 | Not a primary study design |
| 24 | PROPOSTA PARA INSERÇÃO DO FARMACÊUTICO NOS NÚCLEOS AMPLIADOS DE SAÚDE DA FAMÍLIA E ATENÇÃO BÁSICA - NASF-AB | Félix.C | 2023 | Not a primary study design |
| 25 | METODOLOGIAS ATIVAS DE ENSINO APLICADAS À CONSTRUÇÃO DE UM PLANO OPERATIVO PARA ASSISTÊNCIA FARMACÊUTICA AO IDOSO | Araujo.R | 2024 | Did not describe a clinical pharmacy service |
| 26 | FINANCIAMENTO DA ASSISTÊNCIA FARMACÊUTICA E DISPONIBILIDADE DE MEDICAMENTOS BÁSICOS NO MUNICÍPIO DE BELO HORIZONTE | Ahouagi.A | 2022 | Did not describe a clinical pharmacy service |
| 27 | CONSTRUÇÃO DE UM SERVIÇO DE GERENCIAMENTO DA TERAPIA MEDICAMENTOSA NA ATENÇÃO PRIMÁRIA À SAÚDE NO SUS: um projeto de extensão universitária | Silva.D | 2015 | Not a primary study design |
| 28 | DESENVOLVIMENTO DE PROTOCOLO DE INDICADORES PARA AVALIAÇÃO DA CAPACIDADE DE GESTÃO DA ASSISTÊNCIA FARMACÊUTICA NA ATENÇÃO PRIMÁRIA À SAÚDE | Faraco.E | 2016 | Did not describe a clinical pharmacy service |
| 29 | Identidade profissional do farmacêutico: desafios para atuação na atenção primária à saúde | Antunes.R | 2022 | Did not describe a clinical pharmacy service |
| 30 | Análise de programas e ações em assistência farmacêutica para a disponibilidade de medicamentos essenciais no programa saúde da família. | Pereira.L | 2005 | No pharmacists involved |
| 31 | BENZODIAZEPÍNICOS NA ATENÇÃO PRIMÁRIA EM SAÚDE: uma epidemia silenciosa | Sodré.D | 2024 | No pharmacists involved |
| 32 | ORGANIZAÇÃO DA ASSISTÊNCIA FARMACÊUTICA E ACESSO AOS MEDICAMENTOS NA ATENÇÃO PRIMÁRIA DO SISTEMA ÚNICO DE SAÚDE: AVALIAÇÃO DE UMA POLÍTICA PÚBLICA | Barbosa.M | 2018 | Did not describe a clinical pharmacy service |
| 33 | Serviços Farmacêuticos na Atenção Primária à Saúde: Estudo Etnográfico em Serviços de Saúde no município de São Paulo | Mendes.S | 2020 | Not a primary study design |
| 34 | ANÁLISE DA IMPLANTAÇÃO DE SERVIÇOS CLÍNICOS FARMACÊUTICOS NA ATENÇÃO BÁSICA EM UMA REGIÃO DO MUNICÍPIO DE SÃO PAULO | Santos.F | 2017 | Not a primary study design |
| 35 | Curso Serviços Farmacêuticos na Atenção Primária em Saúde no Município do Rio de Janeiro: um estudo da formação profissional | Dóczy.A | 2021 | Did not describe a clinical pharmacy service |
| 36 | Avaliação da rede de atenção em saúde e da farmacoterapia de pacientes internados por Condições Sensíveis a Atenção Primária pelo SUS. | Pereira.M | 2016 | Not conducted in a Primary Health Care setting |
| 37 | FATORES QUE PODEM INFLUENCIAR A DISPONIBILIDADE DOS MEDICAMENTOS ESSENCIAIS NA ATENÇÃO PRIMÁRIA À SAÚDE (APS) DO SISTEMA ÚNICO DE SAÚDE (SUS) | Rocha.W | 2019 | Did not describe a clinical pharmacy service |
| 38 | “Política de assistência farmacêutica no município de João Pessoa-PB: contexto, desafios e perspectivas” | Bruns.S | 2013 | Did not describe a clinical pharmacy service |
| 39 | FENOMENOLOGIA, SAÚDE MENTAL E ATENÇÃO BÁSICA NO BRASIL: Sobre a experiência da pessoa com transtorno psíquico no mundo da vida | Araújo.B | 2018 | No pharmacists involved |
| 40 | TÉCNICOS EM FARMÁCIA EM ATIVIDADES DE ASSISTÊNCIA FARMACÊUTICA NA ATENÇÃO BÁSICA À SAÚDE DO SISTEMA ÚNICO DE SAÚDE: formas de inserção e participação | Stephanelli.L | 2015 | Not conducted in a Primary Health Care setting |
| 41 | Serviços Clínicos Farmacêuticos em Sistemas Universais de Saúde: uma análise comparada entre Inglaterra e Brasil | Cunha.C | 2023 | Did not describe a clinical pharmacy service |
| 42 | ASPECTOS DA SAÚDE DA COMUNIDADE E DO CONTROLE SOCIAL COMO FERRAMENTAS DE OTIMIZAÇÃO DA ATENÇÃO PRIMÁRIA NO SISTEMA ÚNICO DE SAÚDE | Pinto.R | 2014 | No pharmacists involved |
| 43 | RELAÇÃO ENTRE ACESSO A MEDICAMENTOS NO SISTEMA ÚNICO DE SAÚDE E A GESTÃO DO CICLO DA ASSISTÊNCIA FARMACÊUTICA NO CONTEXTO DA PESQUISA NACIONAL SOBRE ACESSO, UTILIZAÇÃO E PROMOÇÃO DO USO RACIONAL DE MEDICAMENTOS | Nogrueira.L | 2023 | No pharmacists involved |
| 44 | POLÍTICA NACIONAL DE PLANTAS MEDICINAIS E FITOTERÁPICOS NO SUS E SUA IMPLEMENTAÇÃO POR MEIO DAS FARMÁCIAS VIVAS | GONDIM.J | 2021 | Not a primary study design |
| 45 | APLICAÇÃO DE RECURSOS FINANCEIROS PARA AQUISIÇÃO DE MEDICAMENTOS PARA ATENÇÃO BÁSICA NOS MUNICÍPIOS BRASILEIROS | Pontes.M | 2015 | No pharmacists involved |
| 46 | CARACTERÍSTICAS DA FORÇA DO TRABALHO DO FARMACÊUTICO NO CUIDADO EM SAÚDE PARA A TUBERCULOSE NOS MUNICÍPIOS DA SUPERINTENDÊNCIA REGIONAL DE SAÚDE DE DIAMANTINA, MINAS GERAIS, BRASIL | Carvalho.I | 2019 | Did not describe a clinical pharmacy service |
| 47 | DESENVOLVIMENTO E VALIDAÇÃO DE UM INSTRUMENTO PARA AVALIAÇÃO DE CONFORMIDADE EM SERVIÇOS DE GERENCIAMENTO DA TERAPIA MEDICAMENTOSA | Meireles.B |  | Not conducted in a Primary Health Care setting |
| 48 | ACESSO AO COMPONENTE ESPECIALIZADO DA ASSISTÊNCIA FARMACÊUTICA: análise centrada no paciente renal crônico e na força de trabalho farmacêutica para obtenção de melhores resultados em saúde | Souto.C | 2018 | Not conducted in a Primary Health Care setting |
| 49 | NÃO ADESÃO AO USO DE MEDICAMENTOS E INSATISFAÇÃO COM OS SERVIÇOS DE ASSISTÊNCIA FARMACÊUTICA POR USUÁRIOS DE MEDICAMENTOS PARA DOENÇAS CRÔNICAS NÃO TRANSMISSÍVEIS: EVIDÊNCIAS DA PESQUISA NACIONAL SOBRE ACESSO, UTILIZAÇÃO E PROMOÇÃO DO USO RACIONAL DE MEDICAMENTOS (PNAUM) | Menolli.P | 2023 | Not a primary study design |
| 50 | LETRAMENTO EM SAÚDE NO CUIDADO FARMACÊUTICO DE PESSOAS COM HIPOTIREOIDISMO | BESSANI.J | 2025 | Did not describe a clinical pharmacy service |
| 51 | AVALIAÇÃO DOS SERVIÇOS FARMACÊUTICOS NA ATENÇÃO PRIMÁRIA À SAÚDE NO CUIDADO AO PACIENTE COM TUBERCULOSE EM UNIDADES DE SAÚDE DO MUNICÍPIO DE SALVADOR-BA | Rodrigues.F | 2014 | Did not describe a clinical pharmacy service |
| 52 | DISPONIBILIDADE DE MEDICAMENTOS PRESCRITOS NA ATENÇÃO PRIMÁRIA | Bueno.M | 2020 | No pharmacists involved |
| 53 | SITUAÇÃO SANITÁRIA DOS MEDICAMENTOS NA ATENÇÃO BÁSICA NO SUS, NAS CAPITAIS DO BRASIL | Perreira.M | 2018 | Did not describe a clinical pharmacy service |
| 54 | CONCEPÇÕES DE MÉDICOS DE FAMÍLIA NO BRASIL E NA ITÁLIA SOBRE AUTONOMIA E SOLIDARIEDADE: IMPLICAÇÕES ÉTICAS PARA O CUIDADO NA ATENÇÃO PRIMÁRIA DE SAÚDE | Lima.R | 2008 | No pharmacists involved |
| 55 | Risco Ambiental Estimado dos Resíduos de Medicamentos Distribuídos pelas Unidades de Atenção Primária à Saúde no Município do Rio de Janeiro | Souza.C | 2022 | Did not describe a clinical pharmacy service |
| 56 | Educação a Distância na formação permanente de farmacêuticos: perspectivas de um processo de avaliação | Leite.M | 2011 | No pharmacists involved |
| 57 | ENSINO-APRENDIZAGEM EM SERVIÇO NA EDUCAÇÃO PARA ATENÇÃO FARMACÊUTICA | Mendonça.S | 2017 | Not a primary study design |
| 58 | Incidentes de segurança do paciente na Atenção Primária à Saúde (APS) de Manaus, AM, Brasil | Aguiar.T | 2020 | No pharmacists involved |
| 59 | AVALIAÇÃO DA ATENÇÃO PRIMÁRIA À SAÚDE NO DIAGNÓSTICO E TRATAMENTO DA HANSENÍASE EM MENORES DE 15 ANOS NO BRASIL | Santos.S | 2023 | No pharmacists involved |
| 60 | Assistência farmacêutica enquanto política pública no município de São Carlos-SP: um olhar a partir do campo CTS | Chenchi.L | 2021 | No pharmacists involved |
| 61 | TESTE DE CAMINHADA DE SEIS MINUTOS E ATENÇÃO FARMACÊUTICA NA AVALIAÇÃO E TERAPIA DE PORTADORES DE INSUFICIÊNCIA CARDÍACA POR DOENÇA DE CHAGAS CRÔNICA | CHAMBELA.M | 2017 | Not conducted in a Primary Health Care setting |
| 62 | Avaliação de conhecimento sobre diabetes em profissionais de assitência em farmacia que atuam em rede pública no município de campinas | Delbom.C | 2018 | Did not describe a clinical pharmacy service |
| 63 | ACESSO A MEDICAMENTOS PRESCRITOS NA ÚLTIMA CONSULTA MÉDICA EM POPULAÇÃO ASSISTIDA POR DIFERENTES MODELOS DE ATENÇÃO BÁSICA NAS REGIÕES SUL E NORDESTE DO BRASIL | Cechin.I | 2013 | No pharmacists involved |
| 64 | PERFIL DO CONSUMO DE FÁRMACOS ANTIDEPRESSIVOS NA ATENÇÃO BÁSICA À SAÚDE EM UM MUNICÍPIO DO OESTE CATARINENSE | PREVEDELLO.P | 2017 | Not a primary study design |
| 65 | O cuidado das Pessoas Vivendo com HIV/AIDS: O processo de descentralização para Atenção Primária à Saúde pela ótica dos profissionais da Estratégia Saúde da Família | Santos.L | 2024 | Did not describe a clinical pharmacy service |
| 66 | PERCEPÇÕES DE GRADUANDOS DO CURSO DE FARMÁCIA DA UFMG SOBRE A PRÁTICA E COMPETÊNCIAS PARA ATUAÇÃO NA FARMÁCIA COMUNITÁRIA | Coelho.M | 2017 | Not conducted in a Primary Health Care setting |
| 67 | “Gastos governamentais com medicamentos nos municípios brasileiros no período de 2009 a 2012” | Cunha.C | 2014 | Not a primary study design |
| 68 | CONTRIBUIÇÃO DOS LABORATÓRIOS FARMACÊUTICOS PÚBLICOS NA POLÍTICA DE AQUISIÇÃO DE MEDICAMENTOS DO MINISTÉRIO DA SAÚDE | Andrade.W | 2014 | Not conducted in a Primary Health Care setting |
| 69 | PRODUÇÃO DE UMA CARTILHA INFORMATIVA PARA PROMOÇÃO DE SAÚDE DE PESSOAS COM E EM RISCO DE DESENVOLVER DIABETES | Pontes.C | 2025 | No pharmacists involved |
| 70 | PROMOÇÃO DO USO RACIONAL DE MEDICAMENTOS: UM OLHAR PARA O COTIDIANO DOS PROFISSIONAIS DA ATENÇÃO BÁSICA | Durigun.V; Durigon.V | 2023 | No pharmacists involved |
| 71 | CARACTERIZAÇÃO DA ASSISTÊNCIA FARMACÊUTICA NO SISTEMA PRISIONAL DO RIO GRANDE DO SUL | Ferreira.M | 2023 | No pharmacists involved |
| 72 | Análise da utilização do Registro Nacional de Preços de Medicamentos como instrumento para aumento da oferta de medicamentos no Sistema Único de Saúde | CÁRDENAS.F | 2015 | Not conducted in a Primary Health Care setting |
| 73 | QUALIDADE E SEGURANÇA DE PRESCRIÇÕES DE MEDICAMENTOS NO ÂMBITO DA ATENÇÃO PRIMÁRIA À SAÚDE | Batista.A | 2022 | No pharmacists involved |
| 74 | POLÍTICAS PUBLICAS DE FITOTERÁPICOS E PLANTAS MEDICINAIS NA ATENÇÃO PRIMÁRIA À SAÚDE: UMA PROPOSTA DE AVALIAÇÃO | Gois.J | 2025 | Not a primary study design |
| 75 | SEGURANÇA DO PACIENTE RELACIONADA A ERROS DE MEDICAÇÃO NA ATENÇÃO PRIMÁRIA À SAÚDE | Santos.A | 2018 | No pharmacists involved |
| 76 | PERFIL DAS SOLICITAÇÕES ADMINISTRATIVAS E JUDICIAIS DE MEDICAMENTOS IMPETRADAS CONTRA A SECRETARIA DE SAÚDE DO ESTADO DO CEARÁ | Pessoa.N | 2007 | Not conducted in a Primary Health Care setting |
| 77 | CONSUMO E GASTOS COM PSICOTRÓPICOS NO SISTEMA ÚNICO DE SAÚDE NO ESTADO DE MINAS GERAIS: ANÁLISE DE 2011 A 2013 | Figeuiredo.A | 2015 | Did not describe a clinical pharmacy service |
| 78 | Fatores associados a não adessão ao tratamento farmacológio em idosos brasileiros: uma revisão sistemática | Silva.W | 2015 | Not a primary study design |
| 79 | PROCESSO DE TRABALHO DO NÚCLEO DE APOIO À SAÚDE DA FAMÍLIA E O DESENVOLVIMENTO DO APOIO MATRICIAL PARA A PRODUÇÃO DO CUIDADO | Oliveira.K | 2014 | Did not describe a clinical pharmacy service |
| 80 | A prática farmaceutica em drogarias do estado de minas gerais | Imbelloni.L | 2023 | Did not describe a clinical pharmacy service |
| 81 | Plantas Medicinais e Fitoterapia: Uma Visão de Profissionais Prescritores da Atenção Primária à Saúde de Juiz de Fora – MG, Brasil | Dibo.V | 2023 | Did not describe a clinical pharmacy service |
| 82 | Análise do consumo de antidepressivos em tratamentos crônicos por pacientes em um município do oeste de Santa Catarina | LAZAROTTO.D | 2019 | No pharmacists involved |
| 83 | O agir cotidiano dos gestores da Estratégia Saúde da Família: possibilidades e desafios | Gomes.F | 2016 | No pharmacists involved |
| 84 | GASTOS PRIVADOS COM MEDICAMENTOS ENTRE USUÁRIOS DA ATENÇÃO BÁSICA | Alves.J | 2022 | Did not describe a clinical pharmacy service |
| 85 | ATENÇÃO EM SAÚDE: a Construção Cotidiana da Integralidade no Município de Catas Altas - MG | Queiróz.E | 2008 | Not conducted in a Primary Health Care setting |
| 86 | AVALIAÇÃO DOS ATRIBUTOS ESSENCIAIS DA ATENÇÃO BÁSICA EM SAÚDE DA CRIANÇA: ESTUDO DE CASO NO DISTRITO DE SAÚDE NORTE DA CIDADE DE MANAUS/AM | SOUZA.S | 2016 | No pharmacists involved |
| 87 | Sistematização da assistência de enfermágemna rede de atençã básicade são leopoldo | Freitas.L | 2019 | No pharmacists involved |
| 88 | ACESSO AOS ANTIPSICÓTICOS ATÍPICOS PARA O TRATAMENTO DE ESQUIZOFRENIA NO SUS: impacto orçamentário e gestão de riscos como ferramentas para orientar a alocação nos componentes da assistência farmacêutica | Felício.L | 2024 | No pharmacists involved |
| 89 | ANÁLISE DO GASTO PÚBLICO COM MEDICAMENTOS DO COMPONENTE BÁSICO DA ASSISTÊNCIA FARMACÊUTICA DO MUNICÍPIO DO JABOATÃO DOS GUARARAPES: TENDÊNCIA 2008 A 2013 | Santos.J | 2014 | No pharmacists involved |
| 90 | Assistência farmacêutica aos portadores de diabetes mellitus: impacto do farmácia popular e do acesso via judicialização da saúde | Vanessa Boarati | 2020 | No pharmacists involved |
| 91 | A IMPLEMENTAÇÃO DA ASSISTÊNCIA FARMACÊUTICA BÁSICA NO NÍVEL MUNICIPAL DO ESTADO DO RIO DE JANEIRO NA PERSPECTIVA DE ATORES RELEVANTES | Silva.S | 2011 | No pharmacists involved |
| 92 | USO DE PSICOFÁRMACOS EM POPULAÇÃO ADULTA COBERTA PELA ESTRATÉGIA DE SAÚDE DA FAMÍLIA | GUIMARÃES.E | 2022 | No pharmacists involved |
| 93 | DESENVOLVIMENTO E VALIDAÇÃO DE ALGORITMO PARA REDUÇÃO DE CARGA ANTICOLINÉRGICA EM PESSOAS IDOSAS DA ATENÇÃO PRIMÁRIA EM SAÚDE | Garcia.T | 2024 | No pharmacists involved |
| 94 | ANÁLISE DA DISPENSAÇÃO DE MEDICAMENTOS PSICOTRÓPICOS EM UM MUNICÍPIO DA TRÍPLICE FRONTEIRA INTERNACIONAL NO PERÍODO PRÉ- PANDÊMICO (2018-2019) E PANDÊMICO (2020-2021) DA COVID-19: UMA CONTRIBUIÇÃO PARA CONSTRUÇÃO DE POLÍTICA DE SAÚDE MENTAL | Tomin.G | 2022 | Not conducted in a Primary Health Care setting |
| 95 | Fatores relacionados à adesão ao tratamento de pessoas adultas com Diabetes mellitus cadastrados no sistema HIPERDIA da atenção primária à saúde | Vicente.N | 2014 | No pharmacists involved |
| 96 | ACESSO A MEDICAMENTOS E FATORES ASSOCIADOS ENTRE USUÁRIOS DOS SERVIÇOS DE ATENÇÃO PRIMÁRIA À SAÚDE EM SALVADOR, BAHIA | Bradão.M | 2023 | No pharmacists involved |
| 97 | Pacientes Pós-Transplante Alogênico de Células Tronco Hematopoiéticas: Estudo da Contribuição da Atenção Farmacêutica nos Resultados Terapêuticos. | CORRÊA.P | 2014 | Not conducted in a Primary Health Care setting |
| 98 | Caracterização da população com Diabetes Mellitus tipo 2 e avaliação da atenção à saúde no Brasil | Rodrigues.J | 2022 | No pharmacists involved |
| 99 | ANÁLISE COMPARATIVA DAS ESTRATÉGIAS DE ABASTECIMENTO DE MEDICAMENTOS DO COMPONENTE BÁSICO, NO ESTADO DE MINAS GERAIS | Dibai.C | 2020 | No pharmacists involved |
| 100 | MEDICAMENTOS POTENCIALMENTE PERIGOSOS NA ATENÇÃO PRIMÁRIA À SAÚDE EM UM MUNICÍPIO DE MÉDIO PORTE DE MINAS GERAIS: DESAFIOS PARA A SEGURANÇA DO PACIENTE | Ferriera.W | 2023 | Did not describe a clinical pharmacy service |
| 101 | AVALIAÇÃO DA ATENÇÃO PRIMÁRIA À SAÚDE: IDENTIFICAÇÃO DOS ATRIBUTOS, INDICADORES DE QUALIDADE E ESTRATÉGIAS DE MELHORIA DOS SERVIÇOS | Ferreira.J | 2017 | No pharmacists involved |
| 102 | Busca por avaliação de próstata, disfunção erétil e demanda oculta de homens na Atenção Primária à Saúde | Modesto.A | 2016 | No pharmacists involved |
| 103 | APLICAÇÃO DE BIOINDICADORES E AVALIAÇÃO CLÍNICA EM TRABALHADORES RURAIS EXPOSTOS AOS PRAGUICIDAS ORGANOFOSFORADOS VISANDO SUBSIDIAR A IMPLANTAÇÃO DE UMA REDE DE ATENÇÃO PRIMÁRIA À SAÚDE | Silvério.A | 2016 | No pharmacists involved |
| 104 | LEVANTAMENTO ETNOFARMACOLÓGICO NOS BAIRROS DO ENTORNO DA RESERVA BIOLÓGICA MUNICIPAL POÇO D’ANTA COBERTOS POR UNIDADES DE ATENÇÃO PRIMÁRIA À SAÚDE | Borcard.G | 2013 | Not conducted in a Primary Health Care setting |
| 105 | RESULTADOS DE UM PROCESSO EDUCATIVO EM ALIMENTAÇÃO E NUTRIÇÃO VIVENCIADO POR UM GRUPO DE MULHERES COM DOENÇAS CRÔNICAS METABÓLICAS | Moura.E | 2013 | No pharmacists involved |
| 106 | ESTRATÉGIA SAÚDE DA FAMÍLIA: avaliação dos cuidados em saúde e nutrição aos adultos diabéticos e hipertensos, Matão/SP. | Guandalini.V | 2013 | No pharmacists involved |
| 107 | DISPONIBILIDADE DE MEDICAMENTOS, ESTRUTURA DE MATERIAIS E CUIDADO OFERTADO A PACIENTES COM HIPERTENSÃO E DIABETES NA ATENÇÃO PRIMÁRIA À SAÚDE: um estudo a partir do Programa Nacional de Melhoria do Acesso e Qualidade. | Dutra.B | 2019 | No pharmacists involved |
| 108 | OS CENÁRIOS DE PRÁTICA DA SAÚDE COLETIVA NA FORMAÇÃO DO PROFISSIONAL FARMACÊUTICO NA GRADUAÇÃO:INTERFACES COM O PENSAMENTO COMPLEXO | Borin.F | 2019 | Did not describe a clinical pharmacy service |
| 109 | ESTRATÉGIAS DE ACESSO À PROFILAXIA PRÉ-EXPOSIÇÃO AO HIV NA ATENÇÃO PRIMÁRIA | Bianco.M | 2023 | Not a primary study design |
| 110 | UTILIZAÇÃO DE PLANTAS MEDICINAIS E FITOTERAPIA NA ESTRATÉGIA SAÚDE DA FAMÍLIA NO MUNICÍPIO DE RECIFE: IMPACTO DE AÇÕES IMPLEMENTADAS SOBRE A PRESCRIÇÃO E RECOMENDAÇÃO | Araújo.D | 2014 | Did not describe a clinical pharmacy service |
| 111 | O Núcleo de Apoio à Saúde da Família/NASF e sua Interface com Atenção Básica: estudo a partir dos dados do Programa de Melhoria do Acesso e da Qualidade da Atenção Básica/ PMAQ | Brocardo.D | 2018 | No pharmacists involved |
| 112 | ACESSO E USO DE MEDICAMENTOS PARA HIPERTENSÃO ARTERIAL SISTÊMICA E DIABETES MELLITUS NO MUNICÍPIO DE SALVADOR – BAHIA: UM ESTUDO TRANSVERSAL | Cruz.J | 2023 | No pharmacists involved |
| 113 | MEDICAMENTOS POTENCIALMENTE INAPROPRIADOS PARA IDOSOS EM UMA UNIDADE BÁSICA DE SAÚDE DE PORTO ALEGRE | Garcia.T | 2019 | No pharmacists involved |
| 114 | ESTRATÉGIAS DE TELEMEDICINA EM APOIO À QUALIFICAÇÃO E A ORIENTAÇÃO DO CUIDADO DE PACIENTES COM DOENÇAS RESPIRATÓRIAS CRÔNICAS: RespiraNet | Molina-Bastos.C | 2022 | Not conducted in a Primary Health Care setting |
| 115 | USO DE MEDICAMENTOS POTENCIALMENTE INADEQUADOS ENTRE IDOSOS NO MUNICÍPIO DE VIÇOSA - MG: UM INQUÉRITO DE BASE POPULACIONAL | Martins.G | 2014 | No pharmacists involved |
| 116 | “Consumo de insulina humana no Brasil: uma análise multivariada” | Botega.A | 2013 | Not conducted in a Primary Health Care setting |
| 117 | Percepções dos PSse pacientes sobre o atendimento à FA (FA) na rede pública de Atenção Primária à Saúde à Saúde brasileira: um estudo de métodos mistos | Paschoal.E | 2024 | Did not describe a clinical pharmacy service |
| 118 | “Desempenho da Atenção Primária e uso racional de medicamentos por pacientes hipertensos e diabéticos no município do Rio de Janeiro” | Mendes.L | 2013 | Did not describe a clinical pharmacy service |
| 119 | VIGILÂNCIA EM SAÚDE NA ATENÇÃO PRIMÁRIA: do trabalho prescrito à gestão coletiva da vigilância em saúde em uma unidade básica de saúde do Distrito Federal | Vieira.D | 2025 | No pharmacists involved |
| 120 | Sistema de notificação de incidentes e gestão da segurança do paciente na atenção primária: descrição, análise e recomendações de melhoria da experiência de um centro de saúde escola | Motta.I | 2021 | No pharmacists involved |
| 121 | COORDENAÇÃO DAS REDES DE ATENÇÃO À SAÚDE NO MUNICÍPIO DE SANTA ROSA, ESTADO DO RIO GRANDE DO SUL, BRASIL | Dadalt.A | 2023 | No pharmacists involved |
| 122 | ANÁLISE DOS FATORES CRÍTICOS NA GESTÃO DE MEDICAMENTOS VISANDO À LOGÍSTICA REVERSA ENTRE INSTITUIÇÕES DE SAÚDE DO SETOR PÚBLICO | Campos.E | 2020 | No pharmacists involved |
| 123 | CONSUMO DE RISCO DE ÁLCOOL ENTRE IDOSOS NA ÁREA DE ABRANGÊNCIA DAS ESTRATÉGIAS SAÚDE DA FAMÍLIA | Vinhote.I | 2014 | No pharmacists involved |
| 124 | Capacitação de profissionais da saúde na detecção precoce do câncer de pele em idosos. | Mendes.A | 2020 | No pharmacists involved |
| 125 | Itinerário terapêutico para acesso a medicamentos de portadores de diabetes mellitus atendidos pelo TEIAS Escola Manguinhos | Rocha.N | 2016 | Not conducted in a Primary Health Care setting |
| 126 | CARACTERIZAÇÃO DOS SERVIÇOS CLÍNICOS PROVIDOS POR FARMACÊUTICOS EM FARMÁCIAS COMUNITÁRIAS NO BRASIL: REVISÃO DE ESCOPO E ESTUDO QUALITATIVO | Garibeli.A | 2016 | Not a primary study design |
| 127 | ANÁLISE DO FINANCIAMENTO PARA AQUISIÇÃO DE MEDICAMENTOS NA ATENÇÃO PRIMÁRIA: INIQUIDADES ENTRE OS MUNICÍPIOS BRASILEIROS | Silva.W | 2024 | No pharmacists involved |
| 128 | O QUE FAZ O FARMACÊUTICO NO NASF? CONSTRUÇÃO DO PROCESSO DE TRABALHO E PROMOÇÃO DA SAÚDE EM UM MUNICÍPIO DO SUL DO BRASIL | Nakamura.C | 2013 | Not a primary study design |
| 129 | GUIA DE PROMOÇÃO DA SAÚDE PARA PESSOA IDOSA | Silva.J | 2018 | Did not describe a clinical pharmacy service |
| 130 | ASPECTOS ESTRUTURAIS DE UNIDADES BÁSICAS DE SAÚDE PARA QUALIDADE DA ATENÇÃO ÀS PESSOAS COM DIABETES MELLITUS | Almeida.T | 2021 | No pharmacists involved |
| 131 | TALIDOMIDA NO SUS: ORGANIZAÇÃO DO CONTROLE DO USO E A EVOLUÇÃO DAS POLÍTICAS PÚBLICAS PARA A REGULAÇÃO | Jesus.S | 2019 | Not a primary study design |
| 132 | INSERÇÃO DE FITOTERÁPICOS INDUSTRIALIZADOS NA ATENÇÃO BÁSICA À SAÚDE NO MUNICÍPIO DE PINHAIS-PR | GRIBNER.C | 2016 | No pharmacists involved |
| 133 | COBERTURA VACINAL DAS CRIANÇAS MENORES DE 5 ANOS NO MUNICÍPIO DE SÃO GABRIEL DA CACHOEIRA,AM 2004- 2009 | Aguiar.A | 2011 | No pharmacists involved |
| 134 | O GASTO REAL COM MEDICAMENTOS PARA DIABETES E HIPERTENSÃO COMPARADO AO GASTO REGISTRADO NO SISTEMA DE INFORMAÇÃO DO HIPERDIA EM UMA PEQUENA CIDADE. | Guilherme.A | 2022 | No pharmacists involved |
| 135 | Utilização de medicamentos em adultos no município de Praia Grande, São Paulo: prevalência e fatores associados | Vieira.M | 2016 | No pharmacists involved |
| 136 | JUDICIALIZAÇÃO DO ACESSO AO TRATAMENTO DO DIABETES NO ESTADO DE MINAS GERAIS | Simões.F | 2015 | No pharmacists involved |
| 137 | Desenvolvimento de filme polimérico oral contendo extrato aquoso de Psidium guajava L. para tratamento da doença periodontal em cães |  |  | Not conducted in a Primary Health Care setting |
| 138 | FORMAÇÃO EM RESIDÊNCIA MULTIPROFISSIONAL E A ATUAÇÃO NA ATENÇÃO BÁSICA EM SAÚDE: UM ESTUDO EM SERVIÇOS PÚBLICOS MUNICIPAIS DA REGIÃO SUL DO BRASIL | Miolo.S | 2018 | No pharmacists involved |
| 139 | PERFIL FARMACOTERAPÊUTICO E FATORES ASSOCIADOS AO USO DE MEDICAMENTOS POTENCIALMENTE INAPROPRIADOS ENTRE IDOSOS ACOMPANHADOS NA ATENÇÃO SECUNDÁRIA | Santos.T | 2018 | Not conducted in a Primary Health Care setting |
| 140 | IMPACTOS DA TELEMEDICINA NO MODELO DE NEGÓCIO DA INDÚSTRIA FARMACÊUTICA SOB O PRISMA DE ENTIDADE REPRESENTATIVA DO SETOR: uma pesquisa participante | Oliveira.S | 2023 | Not conducted in a Primary Health Care setting |
| 141 | PERSPECTIVAS DOS CIRURGIÕES-DENTISTAS SOBRE A INSERÇÃO DA FITOTERAPIA NA ATENÇÃO BÁSICA DE SAÚDE | Júnior.J | 2005 | No pharmacists involved |
| 142 | Proposta para auxílio na estratégia de produção de medicamentos antineoplásicos com risco de desabastecimento no Brasil | Silva.D | 2019 | Not conducted in a Primary Health Care setting |
| 143 | ANÁLISE DAS PRESCRIÇÕES DOS MEDICAMENTOS SUJEITOS AO CONTROLE ESPECIAL NO MUNICÍPIO DE SÃO JOSÉ DOS PINHAIS SEGUNDO OS PROTOCOLOS CLÍNICOS E AS DIRETRIZES TERAPÊUTICAS EM SAÚDE MENTAL | Tristão.M | 2018 | No pharmacists involved |
| 144 | AVALIAÇÃO DA ATENÇÃO PRÉ-NATAL NA ATENÇÃO BÁSICA POR MACRORREGIÕES GEOGRÁFICAS E POR REGIÕES DE SAÚDE | GUIMARÃES.W | 2016 | No pharmacists involved |
| 145 | QUALIDADE EM SAÚDE NA PERSPECTIVA DO TRABALHADOR DA ATENÇÃO BÁSICA NA CIDADE DE MANAUS | Mendes.E | 2015 | No pharmacists involved |
| 146 | AVALIAÇÃO DAS DEMANDAS JUDICIAIS POR ACESSO A MEDICAMENTOS NO DISTRITO FEDERAL | Conti.M | 2013 | Not conducted in a Primary Health Care setting |
| 147 | RESIGNIFICANDO A NATUREZA: A P&D DE MEDICAMENTOS ANTIMALÁRICOS A PARTIR DA ARTEMISIA ANNUA -1960 A 2010. | Sá. I | 2013 | Not conducted in a Primary Health Care setting |
| 148 | ORGANIZAÇÃO DAS EQUIPES MULTIPROFISSIONAIS NA ATENÇÃO BÁSICA NO CEARÁ EM SUA DIMENSÃO POLÍTICA E GERENCIAL NOSSISTEMAS MUNICIPAIS DE SAÚDE | Macedo.M | 2023 | No pharmacists involved |
| 149 | EXAMES CITOPATOLÓGICOS DO COLO DO ÚTERO REALIZADOS NO SISTEMA ÚNICO DE SAÚDE, EM UM MUNICÍPIO DE TRÍPLICE FRONTEIRA NO PARANÁ | TURKIEWICZ.M | 2023 | No pharmacists involved |
| 150 | PRÁTICAS INTEGRATIVAS E COMPLEMENTARES GRUPAIS NOS SERVIÇOS DE SAÚDE DA ATENÇÃO BÁSICA: POSSIBILIDADES DE DIÁLOGO COM A EDUCAÇÃO POPULAR | Nascimento.M | 2016 | Did not describe a clinical pharmacy service |
| 151 | A FITOTERAPIA PRATICADA POR PROFISSIONAIS DE SAÚDE PARTICIPANTES DO CURSO “PLANTAS MEDICINAIS E FITOTERAPIA” REALIZADO PELA SECRETARIA MUNICIPAL DO VERDE E DO MEIO AMBIENTE DE SÃO PAULO (2014/2015) | HARAGUCHI.L | 2018 | Not conducted in a Primary Health Care setting |
| 152 | ADEQUAÇÃO MEDICAMENTOSA AOS CRITÉRIOS DE BEERS E CONDUÇÃO DO TRATAMENTO MEDICAMENTOSO POR IDOSOS ATENDIDOS EM UM AMBULATÓRIO DE GERIATRIA DO INTERIOR DE MINAS GERAIS | Buso.A | 2016 | Not conducted in a Primary Health Care setting |
| 153 | AVALIAÇÃO DE TECNOLOGIAS DE SAÚDE ENVOLVENDO DOENÇAS RARAS E TRATAMENTOS INOVADORES: DOENÇA DE FABRY E TERAPIA DE REPOSIÇÃO ENZIMÁTICA. | Souza.M | 2013 | Not conducted in a Primary Health Care setting |
| 154 | UTILIZAÇÃO, ACESSO, FONTES DE OBTENÇÃO E OFERTA DE PSICOTRÓPICOS NO BRASIL | Rodrigues.P | 2021 | Not conducted in a Primary Health Care setting |
| 155 | PERCEPÇÕES DOS ALUNOS DO CURSO DE AGROPECUÁRIA DO BRASIL SOBRE PLANTAS MEDICINAIS | Almeida.C | 2012 | Not conducted in a Primary Health Care setting |
| 156 | Opening Minds Scale for health care providers (OMS-HC): adaptação cultural para o Brasil | CARRARA.B | 2018 | Not conducted in a Primary Health Care setting |
| 157 | Desafios enfrentados pelos trabalhadores de saúde na utilização do tratamento diretamente observado em pacientes com tuberculose nos municípios do noroeste do Paraná. | VALLOTO.S | 2016 | Did not describe a clinical pharmacy service |
| 158 | PRÁTICAS DE CURA POPULAR USO DE PLANTAS MEDICINAIS E FITOTERAPIA NO PONTO DE CULTURA “OS TESOUROS DA TERRA” E NA REDE FITOVIDA NA REGIAO SERRANA – LUMIAR/ RIO DE JANEIRO (1970-2010) | Ribeiro.P | 2014 | Not conducted in a Primary Health Care setting |
| 159 | ESTUDO DE TENDÊNCIA DO USO DE PSICOFÁRMACOS EM UM MUNICÍPIO DE MINAS GERAIS SOB-RISCO GEOLÓGICO | Carneiro.G | 2021 | No pharmacists involved |
| 160 | GUIA DIGITAL PARA ATUAÇÃO DO PROFISSIONAL DE SAÚDE EM INSULINOTERAPIA: CONSTRUÇÃO E EVIDÊNCIAS DE VALIDADE À LUZ DO LETRAMENTO EM SAÚDE | Cruz.S | 2024 | Not a primary study design |
| 161 | Aspectos Psicossociais na Adesão a Tratamentos da Hipertensão Arterial e Diabetes Mellitus: Concepções e Ações de Agentes Comunitários de Saúde | Nascimento.K | 2017 | No pharmacists involved |
| 162 | PADRÃO DAS PRESCRIÇÕES ODONTOLÓGICAS DE ANTIBIÓTICOS DISPENSADOS NO SISTEMA ÚNICO DE SAÚDE DO ESTADO DE MINAS GERAIS | Santos.J | 2021 | Not conducted in a Primary Health Care setting |
| 163 | USO DE MEDICAMENTOS INIBIDORES DE TIROSINA QUINASE POR PORTADORES DE LEUCEMIA MIELOIDE CRÔNICA NA FUNDAÇÃO HOSPITALAR DE HEMATOLOGIA E HEMOTERAPIA DO AMAZONAS (HEMOAM) | Mendes.A | 2017 | Not conducted in a Primary Health Care setting |
| 164 | Plantas Medicinais: Regulamentações e Arranjos que Transformam Experiências em Mercados | Lemos.S | 2021 | Not conducted in a Primary Health Care setting |
| 165 | CONHECIMENTO E UTILIZAÇÃO DE PLANTAS MEDICINAIS POR PACIENTES COM QUADRO DE ANSIEDADE E DEPRESSÃO: UMA FERRAMENTA PARA EDUCAÇÃO POPULAR EM SAÚDE | Costa.D | 2023 | Not conducted in a Primary Health Care setting |
| 166 | ENVELHECIMENTO ATIVO ENTRE IDOSOS RESIDENTES NA MICRORREGIÃO DE SAÚDE DE UBERABA, MINAS GERAIS | Oliveira.N | 2020 | Not conducted in a Primary Health Care setting |
| 167 | O uso e acesso aos serviços de saúde entre idosos com indicativo de depressão residentes em um município mineiro | Silva.G | 2018 | No pharmacists involved |
| 168 | FATORES ASSOCIADOS À ADESÃO DAS ATIVIDADES DO AUTOCUIDADO DO DIABETES POR IDOSOS | Dyonisio.G | 2016 | Not conducted in a Primary Health Care setting |
| 169 | UTILIZAÇÃO E ACESSO AOS SERVIÇOS DE SAÚDE DE IDOSOS COM SÍNDROME DE FRAGILIDADE | Oliveira.J | 2017 | No pharmacists involved |
| 170 | MORBIDADE E MORTALIDADE RELACIONADAS A MEDICAMENTOS: REVISÃO SISTEMÁTICA E META-ANÁLISE DE ESTUDOS OBSERVACIONAIS | Souza.T | 2013 | Not a primary study design |
| 171 | Desenvolvimento e Avaliação de um Software para Gerenciamento de Dados de Pacientes Hospitalizados | Junio.F | 2023 | Not conducted in a Primary Health Care setting |
| 172 | SEGURANÇA ALIMENTAR E NUTRICIONAL DE PESCADORES E MARISQUEIRAS ARTESANAIS DO LITORAL DA BAHIA EM UM CONTEXTO DE CRISE AMBIENTAL | Santos.T | 2024 | Not conducted in a Primary Health Care setting |
| 173 | UTILIZAÇÃO DE ANSIOLÍTICOS E ANTIDEPRESSIVOS EM MUNICÍPIOS DO MACIÇO DE BATURITÉ-CE: CUSTO, ADESÃO E PERFIL FARMACOEPIDEMIOLÓGICO | Aguiar.C | 2015 | No pharmacists involved |
| 174 | ESTUDO DAS RELAÇÕES ENTRE RESILIÊNCIA, AUTOCUIDADO E CONTROLE METABÓLICO EM PACIENTES COM DIABETES MELLITUS | Araujo.A | 2018 | Not conducted in a Primary Health Care setting |
| 175 | ANÁLISE DA ADESÃO E INTERNAÇÕES DE MÉDIA E ALTA COMPLEXIDADE DE PACIENTES EM TRATAMENTO COM VARFARINA NO MUNICÍPIO DE PORTO ALEGRE – RS | Almeida.L | 2020 | Not conducted in a Primary Health Care setting |
| 176 | FARMACOTERAPIA E FATORES ASSOCIADOS À ADESÃO AO TRATAMENTO EM IDOSOS COM SÍNDROME METABÓLICA | Gomes.N | 2015 | Not conducted in a Primary Health Care setting |
| 177 | Influência do Sistema Produtivo nos Teores de Compostos Fenólicos das Plantas Medicinais: Guaco (Mikania spp.) e Pitanga (Eugenia uniflora) | Bastos.J | 2020 | Not conducted in a Primary Health Care setting |
| 178 | PERSPECTIVAS DAS EQUIPES DO SERVIÇO DE ASSISTÊNCIA DOMICILIAR (SAD) SOBRE ESPECIFICIDADES FISIOTERAPÊUTICAS ANTES E DURANTE A PANDEMIA | Nascimento.I | 2023 | No pharmacists involved |
| 179 | SUBSÍDIOS PARA IMPLANTAÇÃO DO PROGRAMA DE FITOTERAPIA NO MUNICÍPIO DE IMPERATRIZ, ESTADO DO MARANHÃO | Nunes.S | 2010 | Did not describe a clinical pharmacy service |
| 180 | ADOÇÃO DE NOVAS TECNOLOGIAS EM SERVIÇOS PÚBLICOS DE SAÚDE: UMA INVESTIGAÇÃO SOBRE A IMPLANTAÇÃO DO PRONTUÁRIO ELETRÔNICO DO PACIENTE | Cas.E | 2019 | No pharmacists involved |
| 181 | QUALIDADE DE VIDA E AUTOESTIMA DE PESSOAS COM FERIDAS CRÔNICAS | Peres.G | 2018 | Not conducted in a Primary Health Care setting |
| 182 | EFEITOS DA EDUCAÇÃO EM SAÚDE NA PREVENÇÃO DO PÉ DIABÉTICO:REVISÃO SISTEMÁTICA COM META-ANÁLISE | SCHULZ.R | 2021 | Not a primary study design |
| 183 | JOGO “INFECTANDO”: UMA ABORDAGEM LÚDICA E CONTEXTUALIZADA PARA O ENSINO DE DOENÇAS NEGLIGENCIADAS | Pires.F | 2019 | Not conducted in a Primary Health Care setting |
| 184 | CONSTRUÇÃO DE INDICADORES PARA ANÁLISE DE IMPLANTAÇÃO MUNICIPAL DO SISTEMA DE INFORMAÇÕES DO PROGRAMA NACIONAL DE IMUNIZAÇÕES (SIPNI) | Ferreira.P | 2017 | Not conducted in a Primary Health Care setting |
| 185 | FATORES PROGNÓSTICOS DE EVENTOS RECORRENTES LOCAIS, REGIONAIS, METASTÁTICOS E SOBREVIDA GLOBAL EM UMA COORTE POPULACIONAL DE PACIENTES COM CÂNCER CERVICAL | Cobre.A | 2020 | Not conducted in a Primary Health Care setting |
| 186 | SABERES E PRÁTICAS EDUCATIVAS EM ALIMENTAÇÃO E NUTRIÇÃO NO COTIDIANO DE TRABALHADORES DA ESTRATÉGIA SAÚDE DA FAMÍLIA | Pereira.R | 2011 | No pharmacists involved |
| 187 | Produção Financeira das Autorizações de Internação Hospitalar no Hospital Geral de Roraima. | Rocha.B | 2013 | Not conducted in a Primary Health Care setting |
| 188 | FATORES ASSOCIADOS E DURAÇÃO DA INTERNAÇÃO POR TUBERCULOSE EM MANAUS – AMAZONAS, 2010 | Ferreira.A | 2011 | Not conducted in a Primary Health Care setting |
| 189 | O PROGRAMA DE CONTROLE DA TUBERCULOSE DO MUNICÍPIO DE COARI - AMAZONAS: AVALIANDO A SUA IMPLANTAÇÃO E OPERACIONALIZAÇÃO | HEUFEMANN.N | 2010 | No pharmacists involved |
| 190 | ADAPTAÇÃO TRANSCULTURAL E VALIDAÇÃO DA ESCALA DE DEPRESSÃO GERIÁTRICA GDS-15 | Pereira.K | 2017 | Not conducted in a Primary Health Care setting |
| 191 | Desenvolvimento Local e Sustentável da Agricultura Orgânica no município de Queluz, SP, a fim de promover a Soberania e Segurança Alimentar e Nutricional no Programa Nacional de Alimentação Escolar | Rodrigues.D | 2020 | Not conducted in a Primary Health Care setting |
| 192 | DIÁLOGOS INTERSETORIAIS PARA A PROMOÇÃO DA INCLUSÃO EDUCACIONAL E O DESENVOLVIMENTO DAS CRIANÇAS COM A SÍNDROME CONGÊNITA DO ZIKA VÍRUS | Fonseca.J | 2021 | Not conducted in a Primary Health Care setting |
| 193 | Proteção ao Conhecimento Tradicional Associado a Plantas Medicinais: a regulação do acesso ao patrimônio genético e o sistema de patentes | Penteado.I | 2014 | Not conducted in a Primary Health Care setting |
| 194 | FATORES ASSOCIADOS À PERSISTÊNCIA DA DESNUTRIÇÃO EM CRIANÇAS ACOMPANHADAS PELO SISTEMA DE VIGILÂNCIA ALIMENTAR E NUTRICIONAL DE PIRACICABA-SP | Vioto.A | 2010 | Not conducted in a Primary Health Care setting |
| 195 | ANÁLISE DA SITUAÇÃO VACINAL DE IDOSOS EM INSTITUIÇÃO DE LONGA PERMANÊNCIA NO MUNICÍPIO DE UBERABA | OLliveira.V | 2018 | Not conducted in a Primary Health Care setting |
| 196 | VALIDAÇÃO DO WORLD HEALTH ORGANIZATION DISABILITY ASSESSMENT SCHEDULE (WHODAS 2.0) PARA PESSOAS COM OBESIDADE MÓRBIDA | Borges.K | 2017 | Not conducted in a Primary Health Care setting |
| 197 | ESTUDO DO POTENCIAL BIOTECNOLÓGICO DA POLPA DE TUCUMÃ (Astrocaryum aculeatum) IN NATURA E DA CONSERVAÇÃO DAS SUAS PROPRIEDADES NUTRICIONAIS EM EMBALAGENS A VÁCUO | Azevedo.S | 2016 | Not conducted in a Primary Health Care setting |
| 198 | “Determinação eletroanalítica de Losartana® ao patrimônio genético e o sistema de patentes e Alisquieno em fluidos biológicos e em formulaçãoes comerciais | Junior.R | 2016 | Not conducted in a Primary Health Care setting |
| 199 | SUSTENTABILIDADE E USOS DE PLANTAS MEDICINAIS EM UMA VISÃO INTEGRADA À EDUCAÇÃO INDÍGENA E À EDUCAÇÃO ESCOLAR INDÍGENA PAITER | Oliveira.K | 2020 | Not conducted in a Primary Health Care setting |
| 200 | PLANTAS MEDICINAIS E TECNOLOGIAS SOCIAIS PARA O DESENVOLVIMENTO LOCAL NA AMAZÔNIA: A EXPERIÊNCIA ESTADO DO AMAPÁ. | Souza.A | 2016 | Not conducted in a Primary Health Care setting |
| 201 | Remédios e Temperos de Ilha de Guaratiba: Um Diálogo Entre a Etnobotânica e o Desenvolvimento Local, na Zona Oeste do Município do Rio de janeiro | Martins.A | 2023 | Not conducted in a Primary Health Care setting |
| 202 | GEOGRAFIA ECONÔMICA DO SUS BRASILEIRO: AVANÇOS, CRISES E O PAPEL DO ESTADO | HEIMANN.M | 2024 | Not conducted in a Primary Health Care setting |
| 203 | Uso do boldo do telhado verde do Colégio Estadual Erich Walter Heine: uma alternativa no ensino de química | Ribeiro.V | 2019 | Not conducted in a Primary Health Care setting |
| 204 | PROBLEMAS MORAIS E/OU ÉTICOS VIVENCIADOS POR MEMBROS DE COMITÊS DE ÉTICA EM PESQUISA | Barata.R | 2021 | Not conducted in a Primary Health Care setting |
| 205 | ANÁLISE DO IMPACTO DA SÍNDROME PÓS-COVID-19 SOBRE A FUNCIONALIDADE E QUALIDADE DE VIDA DE MULHERES NEGRAS COM DOENÇAS CRÔNICAS NÃO TRANSMISSÍVEIS EM SALVADOR (BA) | Costa.I | 2024 | Not conducted in a Primary Health Care setting |
| 206 | ACOMPANHAMENTO DOS CASOS NOVOS DE TUBERCULOSE: PERFIL CLÍNICO-EPIDEMIOLÓGICO E ORGANIZAÇÃO DO SERVIÇO DE SAÚDE | Sacramento.D | 2015 | No pharmacists involved |
| 207 | AVALIAÇÃO DAS COMPETÊNCIAS NO CONTEXTO DA ATENÇÃO INTEGRAL ÀS DOENÇAS PREVALENTES NA INFÂNCIA DOS RESPONSÁVEIS PELAS CRIANÇAS NO PROGRAMA CAMINHAR DO HOSPITAL UNIVERSITÁRIO BETTINA FERRO DE SOUZA. | Farias.T | 2008 | Not conducted in a Primary Health Care setting |
| 208 | Plantas Medicinais e Uso nas Práticas da Medicina Veterinária na Chapada do Araripe - Região Sul do Ceará | Cardorso.E | 2019 | Not conducted in a Primary Health Care setting |
| 209 | AVALIAÇÃO DO USO DE BENZODIAZEPÍNICOS EM UM MUNICÍPIO DE MÉDIO PORTE DO ESTADO DO PARANÁ | SCALERCIO.P | 2017 | Did not describe a clinical pharmacy service |
| 210 | SENSIBILIDADE MORAL DAS ENFERMEIRAS DOS SERVIÇOS DE ATENÇÃO ÀS URGÊNCIAS | FERRAZ.M | 2023 | Not conducted in a Primary Health Care setting |
| 211 | AVALIAÇÃO DO USO DE GUACO PELO SISTEMA ÚNICO DE SAÚDE NO ESTADO DO RIO GRANDE DO SUL | Santos.L | 2023 | Not conducted in a Primary Health Care setting |
| 212 | Análise do comportamento informacional dos discentes da graduação e da pós-graduação stricto sensu da UFTM sob as perspectivas de acesso e uso do Portal de Periódicos da CAPES | Leal.E | 2018 | Not conducted in a Primary Health Care setting |
| 213 | ESTRATÉGIA SAÚDE DA FAMÍLIA EM ÁREAS RURAIS RIBEIRINHAS AMAZÔNICAS: ESTUDO DE CASO SOBRE A ORGANIZAÇÃO DO TRABALHO EM UMA UNIDADE BÁSICA DE SAÚDE FLUVIAL DE MANAUS. | PUCCIARELLI.M | 2018 | Did not describe a clinical pharmacy service |
| 214 | EDUCAÇÃO EM SAÚDE PARA PACIENTES COM NEOPLASIA GASTROINTESTINAL EM QUIMIOTERAPIA ANTINEOPLÁSICA E A QUALIDADE DE VIDA, CAPACIDADE FUNCIONAL E FADIGA: UM ESTUDO QUASE EXPERIMENTAL | Mendes.L | 2020 | Not conducted in a Primary Health Care setting |
| 215 | ADAPTAÇÃO TRANSCULTURAL SEMÂNTICA E IDIOMÁTICA DE UM INSTRUMENTO DE ADESÃO MEDICAMENTOSA PARA POPULAÇÃO IDOSA | Silva.R | 2020 | No pharmacists involved |
| 216 | Percepção das barreiras e motivações para o tratamento adequado de pacientes com Diabetes Mellitus tipo 1 e tipo 2 assistidos ambulatorialmente na Universidade Federal do Triângulo Mineiro | Tomé.J | 2019 | Not conducted in a Primary Health Care setting |
| 217 | EFEITO DA QUIMIOTERAPIA NA CAPACIDADE FUNCIONAL DE MULHERES COM CÂNCER GINECOLÓGICO, CÂNCER DE MAMA E DOENÇA TROFOBLÁSTICA GESTACIONAL NO SERVIÇO DE ONCO-GINECOLOGIA DE UM HOSPITAL PÚBLICO | Elias.T | 2014 | Not conducted in a Primary Health Care setting |
| 218 | O USO DE PLANTAS MEDICINAIS: DE QUE FORMA A ESCOLA TRABALHA ESSE TEMA? | Ribeiro. I | 2018 | Not conducted in a Primary Health Care setting |
| 219 | Óleos Essenciais com Atividade Antimicrobiana a Fitopatógenos de Interesse em Passiflora edulis Sims | Figueira.A | 2021 | Not conducted in a Primary Health Care setting |
| 220 | SEGURANÇA NA PRESCRIÇÃO DE MEDICAMENTOS DE UM HOSPITAL DE ENSINO | Pires.A | 2016 | Not conducted in a Primary Health Care setting |
| 221 | COMPORTAMENTO REOLÓGICO, ESTABILIDADE FÍSICA, QUALIDADE MICROBIOLÓGICA E COMPOSTOS BIOATIVOS DO SUCO MISTO DE FRUTAS TROPICAIS HOMOGENEIZADO A ALTA PRESSÃO | Carvalho.R | 2022 | Not conducted in a Primary Health Care setting |
| 222 | Triagem de atividade antiviral in vitro (Chikungunya, Mayaro e Zika) de extratos etanólicos de espécies de uso medicinal das famílias Asteraceae e Rutaceae e do fungo Pycnoporus sanguineus | Souza.J | 2024 | Not conducted in a Primary Health Care setting |
| 223 | INFLUÊNCIA DA ADIÇÃO DE DEXTRINA DE TRIGO SOBRE CARACTERÍSTICAS REOLÓGICAS DA MASSA E QUALIDADE DE BISCOITOS | Mansur.C | 2011 | Not conducted in a Primary Health Care setting |
| 224 | ATIVIDADES ANTIBACTERIANA E ANTIBIOFILME DO EXTRATO BRUTO DE Datura suaveolens | Missel.M | 2019 | Not conducted in a Primary Health Care setting |
| 225 | Desenvolvimento de um método de atendimento fundamentado na identificação da não-adesão a farmacoterapia | Oliboni.L | 2017 | Not a primary study design |
| 226 | O MERCADO FARMACÊUTICO DE VAREJO NO DISTRITO FEDERAL DO BRASIL DE 2000 A 2012 | Filho.J | 2013 | No pharmacists involved |
| 227 | “Programa Farmácia Popular no Brasil: uma análise sobre sua relação com o Complexo Econômico-Industrial da Saúde e os programas estratégicos do Governo Federal” | Pereira.M | 2013 | No pharmacists involved |
| 228 | A parceria entre prefeitura de Juiz de Fora e Farmácia Universitária da UFJF: reflexos no ensino, pesquisa e extensão | Xavier.R | 2019 | No pharmacists involved |
| 229 | ANÁLISE DOS SERVIÇOS DE ASSISTÊNCIA FARMACÊUTICA NOS MUNICÍPIOS DA 27a REGIÃO DE SAÚDE DO RIO GRANDE DO SUL | Silveira.T | 2023 | Did not describe a clinical pharmacy service |
| 230 | ReflACTION framework: a proposed model for implementation of clinical pharmacy services | Rocha.K | 2024 | Not conducted in a Primary Health Care setting |
| 231 | Medicamentos isentos de prescrição (MIP): o farmacêutico pode prescrever, mas ele sabe o que são? | DE FARIA MOTA.K | 2019 | Did not describe a clinical pharmacy service |
| 232 | AVALIAÇÃO DA PERCEPÇÃO DO ESTUDANTE NO ALCANCE DOS OBJETIVOS DE APRENDIZAGEM E MÉTODOS DE ORIENTAÇÃO ACADÊMICA EM UM ESTÁGIO EM FARMÁCIA COMUNITÁRIA | Romani.L | 2020 | Not conducted in a Primary Health Care setting |
| 233 | INDICADORES DE ESTRUTURA E PROCESSO NA IMPLEMENTAÇÃO DE UM SERVIÇO DE REVISÃO DA FARMACOTERAPIA EM AMBULATÓRIO | Marques.T | 2015 | Not conducted in a Primary Health Care setting |
| 234 | COORDENAÇÃO DAS REDES DE ATENÇÃO À SAÚDE NO MUNICÍPIO DE SANTA ROSA, ESTADO DO RIO GRANDE DE SUL, BRASIL | Dadalt.A | 2023 | No pharmacists involved |
| 235 | Economic Evaluation of Pharmacist-led Medication Review in the Primary Health Care Centres in Chile | Canale.A | 2020 | Not a primary study design |
| 236 | Clinical impact of medication reviews with follow-up in cardiovascular older adult patients | Mardones.F | 2020 | Not a primary study design |
| 237 | Avaliação da capacidade de gestão da Assistência Farmacêutica na AtençãoPrimária à Saúde nos municípios brasileiros | Faraco. E | 2021 | Did not describe a clinical pharmacy service |
| 238 | SEGURANÇA DO PACIENTE NAS UNIDADES DE PRONTO ATENDIMENTO EM SALVADOR, BAHIA, BRASIL | Nogueira.G | 2022 | Not conducted in a Primary Health Care setting |
| 239 | Ansiolíticos e antidepressivos dispensados na Atenção Básica: análise de custos e interações medicamentosas | Aguiar.C | 2016 | No pharmacists involved |
| 240 | Avaliação do Programa de Controle da Tuberculose por Pacientes e Profissionais do Recife e Propostas de Melhoria por Especialistas: Desafios e Perspectivas | Bezerra.S | 2017 | Not conducted in a Primary Health Care setting |
| 241 | Pharmaceutical services in a Mexican pain relief and palliative care institute | GUTIÉRREZ.R | 2007 | Did not describe a clinical pharmacy service |
| 242 | Primary health care pharmacists and vision for community pharmacy and pharmacists in Chile | MARTÍNEZ-MARDONES.F | 2020 | No pharmacists involved |
| 243 | A proposal of a standard structure and approach for Pharmaceutical Care Case Reports | Martins. R | 2011 | Not a primary study design |
| 244 | Towards a transformation in the pharmacist care paradigm: A scoping study | López.J | 2017 | Not a primary study design |
| 245 | Creating a desired future for pharmacy education and professional practice in Argentina: Summary of a planning workshop | Olivera.M | 2018 | Not conducted in a Primary Health Care setting |
| 246 | Hospital Nuclear Pharmacy Survey: Preliminary Aspects In Brazil | Brasil.M |  | Not conducted in a Primary Health Care setting |
| 247 | Pharmacist-­‐administered immunizations in British Columbia An exploration of current practices and perspectives | Fletcher.A | 2014 | Not conducted in a Latin American country |
| 248 | Comprehensive Medication Management Services as a Solution to Medication Mismanagement: A European Perspective | Mucalo.I |  | Not conducted in a Latin American country |
| 249 | Quitting patterns and success rates of a tobacco cessation program led by New Mexico Pharmaceutical Care Foundation | Shen.X | 2012 | Not conducted in a Latin American country |
| 250 | Levantamento dos Cursos de Farmácia do Sul do Brasil que Oferecem Disciplina de Radiofármacos (ou Similar) | Roeckera.G | 2012 | Not conducted in a Primary Health Care setting |
| 251 | Improving Student Confidence in Skill Performance in a Pharmaceutical Care Lab Setting | Bond.R | 2012 | Not conducted in a Primary Health Care setting |
| 252 | Expanding the Horizon: Global Health Management for Pharmacy Students | Chang.A | 2017 | Not conducted in a Primary Health Care setting |
| 253 | Impact of pharmacist interventions on drug-related problems and laboratory markers in outpatients with human immunodeficiency virus infection | Rezende.C | 2014 | Not conducted in a Primary Health Care setting |
| 254 | ARIA digital anamorphosis: Digital transformation of health and care in airway diseases from research to practice | Bousquet.J | 2020 | Not conducted in a Primary Health Care setting |
| 255 | Evaluation of pharmaceutical assistance in public primary care in Brasília, Brazil Avaliação da assistência farmacêutica na atenção primária no Distrito Federal | Naves.J | 2005 | Did not describe a clinical pharmacy service |
| 256 | Community pharmacy–based H. pylori screening for patients with uninvestigated dyspepsia | Papastergiou.J | 2020 | Not conducted in a Primary Health Care setting |
| 257 | Compreendendo a provisão de um serviço clínico na saúde mental e o papel do farmacêutico: uma análise qualitativa | Fernandes.S | 2021 | Not conducted in a Primary Health Care setting |
| 258 | Caracterización de la prestación del servicio farmacéutico en el departamento de Sucre, Colombia | Borja.D | 2019 | Not conducted in a Primary Health Care setting |
| 259 | Políticas americanas de salud y de formación farmacéutica | Rodríguez.C | 2011 | Not conducted in a Primary Health Care setting |
| 260 | A Comparative Study of Pharmacy Practices between the United States and Ecuador | Yi.S | 2010 | Not conducted in a Primary Health Care setting |
| 261 | An overview of pharmacist roles in palliative care: a worldwide comparison | Krzyżaniak.N | 2007 | Not conducted in a Latin American country |
| 262 | Strategy to promote research and innovation in the pharmaceutical sector through interdisciplinary education | Rocha.M | 2018 | Not conducted in a Primary Health Care setting |
| 263 | Uso de medicamentos e a segurança do paciente na interface entre hospital, atenção básica e domicílio | Marques.L | 2013 | Not conducted in a Primary Health Care setting |
| 264 | Contribuciones a la implementación de un programa de atención farmacéutica para paciente ambulatorio en un hospital de tercer nivel de Bogotá D.C., II-2005 | Muños.I | 2006 | Not conducted in a Primary Health Care setting |
| 265 | An Exploratory Study of Pharmacists' Professional Learning | Wittsrtom.K | 2012 | Not conducted in a Primary Health Care setting |
| 266 | Next‐generation ARIA care pathways for rhinitis and asthma: a model for multimorbid chronic diseases | Bounquet.J | 2019 | Not conducted in a Primary Health Care setting |
| 267 | Assistência farmacêutica discursos e práticas na capital do Império do Brasil (1850-1880)* | Velloso.V | 2010 | Not conducted in a Primary Health Care setting |
| 268 | Avaliação da farmacoterapia no âmbito hospitalar veterinário como ferramenta de promoção na segurança do paciente | Martins.M |  | Not conducted in a Primary Health Care setting |
| 269 | Who cites non-English-language pharmaceutical articles? | Edouard.B | 2010 | Not conducted in a Primary Health Care setting |
| 270 | Training pharmacy workers in recognition, management, and prevention of STDs: district-randomized controlled trial | Garcia.P | 2003 | Did not describe a clinical pharmacy service |
| 271 | What are the differences among occupational groups related to their palliative care- specific educational needs and intensity of interprofessional collaboration in long-term care homes? | Kaasalainen.S | 2014 | Not conducted in a Primary Health Care setting |
| 272 | Hygiene and mental health: body care in the clinical intervention in a CAPSI | Jr, Valdinei Santos de Aguiar | 2017 | Not conducted in a Primary Health Care setting |
| 273 | Impacto de la intervención farmacéutica en la adherencia al tratamiento antirretroviral en pacientes de un hospital de Lima (Perú). | Tafur Valderrama, E.J. | 2012 | Not conducted in a Primary Health Care setting |
| 274 | Impact of a pharmaceutical care program on clinical evolution and antiretroviral treatment adherence: a 5-year study | Arroyo.M | 2013 | Not conducted in a Primary Health Care setting |
| 275 | Expanding access to NCD services via community retail pharmacies in LMICs: a systematic review of the literature | Gentilini.A | 2025 | Not conducted in a Primary Health Care setting |
| 276 | Elaboración de un procedimiento para la aplicación de Buenas Prácticas de Almacenamiento y Etiquetado seguro de medicamentos en la Farmacia Institucional del Hospital Vicente Corral Moscoso de la Ciudad de Cuenca – Ecuador. | Capa.J | 2022 | Not conducted in a Primary Health Care setting |
| 277 | Seguimiento farmacoterapéutico ambulatorio en pacientes hipertensos de un centro de salud del Perú. Estudio cuasi- experimental. | Pinillos-Pozo.V | 2022 | Not conducted in a Primary Health Care setting |
| 278 | Pharmaceutical Health Care Education by application of tutorial teaching [Educar en Atención Farmacéutica aplicando enseñanza tutorial] | Bertoldo, Pamela |  | Not conducted in a Primary Health Care setting |
| 279 | INCIDENCIA DE LAS DINAMICAS MIGRATORIAS VENEZOLANAS HACIA COLOMBIA EN EL SECTOR PETROLERO Y COMERCIAL FARMACEUTICO COLOMBIANO 2005-2011 | Ardila.L | 2012 | Not conducted in a Primary Health Care setting |
| 280 | Worldwide Organization of Neurocritical Care: Results from the PRINCE Study Part 1 | Suarez.J; Suarez, J.I | 2020 | Not conducted in a Primary Health Care setting |
| 281 | Automedicación en estudiantes de la Sede Neiva de la Universidad Cooperativa de Colombia | Ortiz.C | 2019 | Not conducted in a Primary Health Care setting |
| 282 | Perfil farmacoterapêutico em um Centro de Atenção Psicossocial Álcool e Drogas (CAPS- ad) do Nordeste brasileiro | Ferreira.A | 2019 | Not conducted in a Primary Health Care setting |
| 283 | Impact of discharge medication counseling in the cardiology unit of a tertiary hospital in Brazil: A randomized controlled trial | Bonetti.A | 2018 | Not conducted in a Primary Health Care setting |
| 284 | Evaluación de los Conocimientos, Actitudes y Prácticas de Farmacovigilancia a los Farmacéuticos de Hospitales Públicos y Privados Registrados en la Unidad Zonal de Farmacovigilancia, Coordinación Zonal 6 – Ecuador | Cruz.M | 2023 | Not conducted in a Primary Health Care setting |
| 285 | Effects of Phenytoin and Carbamazepine on Calcium Transport in Caco-2 Cells | Borstel.M | 2006 | Not conducted in a Primary Health Care setting |
| 286 | Development of a stepwise tool to aide primary health care professionals in the process of deprescribing in older persons | De la salas.R | 2020 | No pharmacists involved |
| 287 | Análise de risco do processo de administração de medicamentos por via intravenosa em pacientes de um Hospital Universitário de Goiás | Silva.A | 2008 | Not conducted in a Primary Health Care setting |
| 288 | Perfil da utilização de medicamentos não licenciados e sem indicação para crianças em UTI Neonatal de Hospital Universitário de Média Complexidade | Brassica.S | 2009 | Not conducted in a Primary Health Care setting |
| 289 | Diagnosis and treatment of presumed STIs at Mexican pharmacies: survey results from a random sample of Mexico City pharmacy attendants | A N Turner | 2003 | Did not describe a clinical pharmacy service |
| 290 | Judicial litigations and social welfare: access to medicines in two towns in the Santa Catarina State, Brazil | Leite.S | 2012 | Not conducted in a Primary Health Care setting |
| 291 | Are There Disparities in Health Information Access Among New Mexico Practitioners? Results of a Study | Bradley.P | 2014 | Not conducted in a Primary Health Care setting |
| 292 | Evaluación de la prescripción médica en un Instituto de Previsión Social (IPS) de la Ciudad de Cartagena, Colombia | León-Méndez.G | 2019 | Not conducted in a Primary Health Care setting |
| 293 | Seguimiento farmacoterapéutico durante la hospitalización a pacientes transplantados en la fundación clínica Valle del Lili (Cali – Colombia) | Salcedo.J | 2004 | Not conducted in a Primary Health Care setting |
| 294 | Conhecimento e atitudes de farmacêuticos comunitários na dispensação de medicamentos para gestantes | Baldo.J | 2006 | Did not describe a clinical pharmacy service |
| 295 | Implementation of an online emergency education programme at a public university in Mexico | González-Barranco.P | 2020 | Not conducted in a Primary Health Care setting |
| 296 | Cualificación y experiencia de los vendedores de droguería para la dispensación de amoxicilina en una comuna de Santiago de Cali, Colombia | Espinosa.J | 2017 | Not conducted in a Primary Health Care setting |
| 297 | Imcompatibilidade e interações medicamento enteral-alimento em pacientes do hospital Edgardo Rebagliati Martins | Rodrigués.P | 2004 | Not conducted in a Primary Health Care setting |
| 298 | Collaboration between private pharmacies and national tuberculosis programme: an intervention in Bolivia | Lambert.M; Lambert | 2005 | Not conducted in a Primary Health Care setting |
| 299 | Profile of drug utilization in the elderly living in Porto Alegre, Brazil | FAGGIANI.F | 2007 | Not conducted in a Primary Health Care setting |
| 300 | Del ficticio entusiasmo: el mercado de las drogas en el tránsito a la prohibición en Chile. 1920-1960 |  | 2009 | Not conducted in a Primary Health Care setting |
| 301 | The Role of Forecasting, Price Negotiation and Procurement Management in Determining Availability of Antiretroviral Medicines (ARVs) in Mexico | Adesina.A | 2010 | Not conducted in a Primary Health Care setting |
| 302 | Uso de omeprazol em hospital universitário de Porto Alegre-RS (Brasil) | Munari.L | 2004 | Not conducted in a Primary Health Care setting |
| 303 | Evaluation of Over-The-Counter Sales as a Syndromic Surveillance Method for Waterborne Disease in New Mexico | Bustumante.C | 2005 | Not conducted in a Primary Health Care setting |
| 304 | Prevalencia de las I.R.A y uso de antibioticos en pampatar | Evelino.R | 2000 | Not conducted in a Primary Health Care setting |
| 305 | ESTUDO DE UTILIZAÇÃO DE ANTI-ULCEROSOS NA POPULAÇÃO IDOSA DE PORTO ALEGRE, RS, BRASIL | Schroeter.G | 2008 | Not conducted in a Primary Health Care setting |
| 306 | MÉTODO DÁDER DE SEGUIMIENTO FARMACOTERAPÉUTICO Y FARMACOVIGILANCIA EN INSTITUCIONES DE SALUD DE COLOMBIA | AMARILES.P | 2005 | Not conducted in a Primary Health Care setting |
| 307 | Centros de Atenção Psicossocial da região Macrorregional Noroeste de Minas Gerais – descrição do perfil profissional de suas equipes e de suas práticas | Cunha.V | 2011 | Not conducted in a Primary Health Care setting |
| 308 | Produção científica brasileira na área de atenção farmacêutica entre 1990 e 2007 | Witzel.M | 2009 | Not conducted in a Primary Health Care setting |
| 309 | Avaliação econômica do impacto da atividade de Atenção Farmacêutica na assistência à saúde: aspectos metodológicos | Carvalho.F | 2007 | Not conducted in a Primary Health Care setting |
| 310 | O FARMACÊUTICO NO SERVIÇO PÚBLICO DE SAÚDE: A EXPERIÊNCIA DO MUNICÍPIO DE RIBEIRÃO PRETO - SP | Mestriner.D | 2003 | No pharmacists involved |
| 311 | Caracterização das atividades para melhoria da adesão à TARV em serviços de saúde do SUS no Estado de São Paulo | CARACIOLO.J | 2010 | Not conducted in a Primary Health Care setting |
| 312 | A relação do sistema jurídico e do sistema político na garantia do direito social à assistência farmacêutica: o caso do Estado de São Paulo | Marques.S | 2005 | Not conducted in a Primary Health Care setting |
| 313 | Intervenção farmacêutica na qualidade assistencial e nas reações adversas da amitriptilina prescrita para pacientes ambulatoriais do Sistema Único de Saúde de Ribeirão Preto (SP) | Sebastião,E | 2005 | Not conducted in a Primary Health Care setting |
| 314 | Benefício da preparação de nutrição parenteral em uma central de preparação de produtos estereis | Muñoz | 1999 | Not conducted in a Primary Health Care setting |
| 315 | O ri entação farm a c ê utica para DST: uma proposta de sistematização | Neves.J |  | Did not describe a clinical pharmacy service |
| 316 | Práticas de atendimento a DST nas farmácias do Distrito Federal, Brasil: um estudo de intervenção | Neves.J |  | No pharmacists involved |
| 317 | Conhecimentos, práticas e percepção de risco do uso de medicamentos no Distrito Federal | Almeida.MR | 2011 | Not conducted in a Primary Health Care setting |
| 318 | OS TRAÇOS IDENTITÁRIOS DE PROFISSIONAIS QUE ATUAM EM UM SERVIÇO SUBSTITUTIVO DE SAÚDE MENTAL DE BELO HORIZONTE1 | Brito.M | 2009 | Not conducted in a Primary Health Care setting |
| 319 | Consejería Farmaceutica como estrategia em la prevencion de enfermedades durante viajes escolares turisticos en el peru | Ulloa.M | 2006 | Not conducted in a Primary Health Care setting |
| 320 | Formulação de uma Política de Assistência Farmacêutica: a experiência de Goiás | Naves.J |  | Not conducted in a Primary Health Care setting |
| 321 | Análise dos indicadores de prescrição médica em uma unidade de saúde de Anápolis-GO | Borges.L |  | No pharmacists involved |
| 322 | Global restriction of the over-the-counter sale of antimicrobials does it make sense? | Carl Llor | 2024 | Not conducted in a Primary Health Care setting |
| 323 | Description of an elective course about mental illness and treatment in the movies | Cates.M | 2019 | Not conducted in a Primary Health Care setting |
| 324 | ANÁLISE DA ORGANIZAÇÃO DA ASSISTÊNCIA FARMACÊUTICA EM MUNICÍPIOS A LUZ DA POLÍTICA NACIONAL DE MEDICAMENTOS | Lacerda.R | 2013 | No pharmacists involved |
| 325 | Circulation of Quina and Colonial Medical Culture in the New Kingdom of Granada at the End of the Eighteenth Century | Oliveiros.M | 2017 | Not conducted in a Primary Health Care setting |
| 326 | Anti-influenza hyperimmune intravenous immunoglobulin for adults with influenza A or B infection (FLU-IVIG): a double-blind, randomised, placebo-controlled trial | Davey jr.R | 2019 | Not conducted in a Primary Health Care setting |
| 327 | Does Labor Market Concentration Decrease Wages? Evidence from a Retail Pharmacy Merger |  | 2022 | Not conducted in a Primary Health Care setting |
| 328 | Medicamentos de venta bajo receta médica retenida: caracterización y análisis desde la dispensación | Cancino.F | 2014 | Not conducted in a Primary Health Care setting |
| 329 | manejo del dolor dentario de los pobladores de la zona rural y urbana del distrito de otuzco | Acevedo.C | 2003 | Not conducted in a Primary Health Care setting |
| 330 | Adherence to Non-Selective Beta Blockers for Prevention of Variceal Bleeding in Cirrhotic Patients | Yan.An | 2021 | Not conducted in a Primary Health Care setting |
| 331 | Use of statins and the risk of developing pre-diabetes and diabetes: a longitudinal study in individuals aged 40 years or older | Curiaki.G | 2020 | Not conducted in a Primary Health Care setting |
| 332 | ASSISTÊNCIA FARMACÊUTICA A IDOSOS INSTITUCIONALIZADOS DO DISTRITO FEDERAL | Oliveira.M | 2008 | Not conducted in a Primary Health Care setting |
| 333 | Juan de Jesús Rodríguez Robayo y Juan R. Robayo |  |  | Not conducted in a Primary Health Care setting |
| 334 | MEJORANDO LOS SERVICIOS FARMACÉUTICOS HOSPITALARIOS: LA ESTRATEGIA PARA LA IMPLEMENTACIÓN DE LAS BUENAS PRÁCTICAS DE ELABORACIÓN POR PARTE DE LOS PROCESOS DEL SERVICIO FARMACÉUTICO DE LA FUNDACIÓN VALLE DEL LILI | PRETELT.J | 2011 | Not conducted in a Primary Health Care setting |
| 335 | Economic Evaluations of HPV Vaccination in Targeted Regions of Low- and Middle-Income Countries: A Systematic Review of Modelling Studies | Frianto.D | 2022 | Not conducted in a Primary Health Care setting |
| 336 | Empire's experts : the politics of knowledge in Spain's royal monopoly of quina (1751-1808) | Crawford.M | 2009 | Not conducted in a Primary Health Care setting |
| 337 | Impact of the Medicare Part D coverage gap on prescription drug utilization and medication adherence | Rupali.N | 2011 | Not conducted in a Latin American country |
| 338 | A ATENÇÃO FARMACÊUTICA NAS FARMÁCIAS COMUNITÁRIAS DO MUNICÍPIO DE CAMPINA GRANDE DO SUL | Bareta.G | 2003 | Did not describe a clinical pharmacy service |
| 339 | Estudo de utilização de medicamentos em idosos atendidos pelo Sistema Único de Saúde (SUS) | Baldoni. A | 2010 | No pharmacists involved |
| 340 | ASSISTÊNCIA FARMACÊUTICA EM INSTITUIÇÃO DE LONGA PERMANÊNCIA PARA IDOSOS | Gerlak .L | 2012 | Not conducted in a Primary Health Care setting |
| 341 | O COTIDIANO DAS FARMÁCIAS DE MANIPULAÇÃO | Miguel.M | 2020 | Not conducted in a Primary Health Care setting |
| 342 | Apostes del Dr.Angelo maldonado a la enseñanza quimico farmaceutica, industria farmaceutica y a la defensa de la profesion farmaceutica | Terán.L | 2002 | Not conducted in a Primary Health Care setting |
| 343 | IMPACTO DEL SEGUIMIENTO FARMACOTERAPÉUTICO EN LA CALIDAD DE VIDA RELACIONADA A LA SALUD DE PACIENTES CON HIPERTENSION ARTERIAL | Ayala.L | 2010 | Not conducted in a Primary Health Care setting |
| 344 | Consejería farmacéutica: estrategia para mejorar el nivel de conocimiento de la medicación en pacientes con Síndrome Coronario Agudo hospitalizados en el Servicio de Cardiología – H.N.E.R.M (mayo- octubre 2006) | Araoz.M | 2007 | Not conducted in a Primary Health Care setting |
| 345 | Detección de errores de medicación en la administración de medicamentos | Cortés.M | 2000 | Not conducted in a Primary Health Care setting |
| 346 | Adherencia a la terapia farmacológica y sus factores determinantes en pacientes con tuberculosis de un centro de salud de Santiago de Cali | Alviz.E | 2014 | Not conducted in a Primary Health Care setting |
| 347 | A PARTICIPAÇÃO POLÍTICA DOS ATORES COLETIVOS DO CAMPO POPULAR NO MOVIMENTO DE REFORMA NA SAÚDE NO RIO GRANDE DO SUL | David.C | 2014 | No pharmacists involved |
| 348 | Perfil de los farmacéuticos e indicadores de estructura y proceso en farmacias de Curitiba – Brasil | Correr.C | 2004 | Not conducted in a Primary Health Care setting |
| 349 | Sistema Brasileiro de Informação sobre Medicamentos – SISMED | Vidotti.C |  | Not conducted in a Primary Health Care setting |
| 350 | Disentangling residents' engagement with communities of clinical practice in the workplace | Olmos-Vega.F | 2019 | Not conducted in a Primary Health Care setting |
| 351 | Ciencia, docencia y patria: el legado integral del Dr. Maldonado en la farmacia peruana | Guillen.J | 2025 | Not conducted in a Primary Health Care setting |
| 352 | Implementación de las Buenas Prácticas de Almacenamiento en el almacén especializado de medicamentos del Hospital Belén de Trujillo, 2011 | Cortijo.G | 2012 | Not conducted in a Primary Health Care setting |
| 353 | Acontecendo o cuidado ‘do nós’ nos movimentos e ondulações DOS PROCESSOS INTERATIVOS NO AMBIENTE HOSPITALAR1 | Baggio.M | 2016 | Not conducted in a Primary Health Care setting |
| 354 | PREPARO E ADMINISTRAÇÃO DE MEDICAMENTOS: ANÁLISE DE QUESTIONAMENTOS E INFORMAÇÕES DA EQUIPE DE ENFERMAGEM | Silva.D | 2007 | Not conducted in a Primary Health Care setting |
| 355 | AUTONOMIA Y SUBORDINACION: LOS FARMACEUTICOS DIPLOMADOS Y LA CONSTITUCION DE UN CAMPO MEDICO EN BUENOS AIRES (1852-1880) | Leandro.R | 1998 | Not conducted in a Primary Health Care setting |
| 356 | O POTENCIAL DA EDUCAÇÃO EM SAÚDE NA ALTA HOSPITALAR PARA A AUTONOMIA EM CUIDADOS PALIATIVOS: UM OLHAR ENFERMEIRO | Marcelino.S |  | Not conducted in a Primary Health Care setting |
| 357 | Automedicação: uma abordagem qualitativa de suas motivações | Naves.J |  | No pharmacists involved |
| 358 | AS PRESCRIÇÕES MÉDICAS COMO CAUSADORAS DE RISCO PARA PROBLEMAS RELACIONADOS COM OS MEDICAMENTOS | Lyra.D | 2004 | Did not describe a clinical pharmacy service |
| 359 | Profile of drug interactions in hospitalized children | Martinbiancho.J | 2007 | Not conducted in a Primary Health Care setting |
| 360 | Comparison of monotherapy with angiotensin- converting enzyme inhibitors or angtiotensin receptor blockers in improving health outcomes among veteran patients with type 2 diabetes | Campbell.H | 2011 | Not conducted in a Primary Health Care setting |
| 361 | Búsqueda de un Nuevo Perfil para el Farmacéutico: la Readaptación de los Currículos en Cursos de Farmacia | Carpes.A | 2009 | Not conducted in a Primary Health Care setting |
| 362 | JUDICIALIZAÇÃO DA POLÍTICA DE ASSISTÊNCIA FARMACÊUTICA: DISCUSSÃO SOBRE AS CAUSAS DE PEDIR NO DISTRITO FEDERAL. | Honorato.S | 2014 | Not conducted in a Primary Health Care setting |
| 363 | Five-year review of an international clinical research-training program | Suemoto.C | 2015 | Not conducted in a Primary Health Care setting |
| 364 | EFECTO HIPOGLICEMIANTE DEL EXTRACTO ACUOSO DE LAS HOJAS DE Smallanthus sonchifolius (YACÓN) EN PACIENTES CON DIABETES MELLITUS TIPO | Gordillo | 2012 | Not conducted in a Primary Health Care setting |
| 365 | Entrevista nº954 | Eisser.M | 2000 | Not conducted in a Primary Health Care setting |
| 366 | A ATUAÇÃO DA ANVISA NA QUESTÃO DO ACESSO POPULACIONAL AOS MEDICAMENTOS | Silvia.M | 2016 | Not conducted in a Primary Health Care setting |
| 367 | Building a measurement framework of burden of treatment in complex patients with chronic conditions: a qualitative study | Eton.D | 2012 | Not conducted in a Primary Health Care setting |
| 368 | Farmacoeconomia: um instrumento para gestão em drogarias de bairro | Nacimento.J | 2014 | Not conducted in a Primary Health Care setting |
| 369 | Impact of Pharmaceutical Care interventions in the identifi cation and resolution of drug-related problems and on quality of life in a group of elderly outpatients in Ribeirão Preto (SP), Brazil | Lyra.D | 2007 | Not conducted in a Primary Health Care setting |
| 370 | Entrevista nº959 | Arredondo.C | 1973 | Not conducted in a Primary Health Care setting |
| 371 | Disenõ e implementación de una estrategia para el mejoramento del cumplimento farmacoterapeutico en el paciente hipertenso | Bernal.I | 2001 | Not conducted in a Primary Health Care setting |
| 372 | El vidrio en la Casa del Ap artad o, siglos XVI- XVIII | Rodríguez.J | 2010 | Not conducted in a Primary Health Care setting |
| 373 | MEDICAMENTOS DE RIESGO EN EL ÁMBITO OCUPACIONAL |  | 2022 | Not conducted in a Primary Health Care setting |
| 374 | Customer perceptions of perceived risk in generic drugs: the Spanish market | Suplet.M | 2009 | Not conducted in a Primary Health Care setting |
| 375 | LEGISLACIÓN SANITARIA Y BOTICAS NOVOHISPANAS | Rodríguez.M |  | Reason not specified in the screening sheet |
| 376 | E-Learning Practices on Healthcare Associated Infections (HAI): Report of Brazilian Experience | Medeiros.E | 2008 | Not conducted in a Primary Health Care setting |
| 377 | REZADEIRAS – UMA FÉ POPULAR | Pimentel.C | 2007 | Not conducted in a Primary Health Care setting |
| 378 | Reações adversas a amitriptilina relatadas por pacientes ambulatoriais | Sebastião.E | 2005 | Not conducted in a Primary Health Care setting |
| 379 | Biowaiver Monographs for Immediate Release Solid Oral Dosage Forms: Amitriptyline Hydrochloride | Manzo.R | 2006 | Not conducted in a Primary Health Care setting |
| 380 | ATENÇÃO DO PROFISSIONAL FARMACÊUTICO NA SAÚDE INDÍGENA: ORIENTAÇÃO E CUIDADO | da Silva, Silvana Souza and da Silva, Geovana Souza and de Souza Santos, Bruna Alves and da Silva Ramos, Karla Arianny and da Silva, Gessica Marques and Silva Freitas, Wagner and Serrão Pinto, Thiago | 2025 | Not a primary study design |
| 381 | ATENÇÃO FARMACÊUTICA AO IDOSO PORTADOR DE DIABETES TIPO 2: MANEJO DE POLIFARMÁCIA E RISCOS DE HIPOGLICEMIA. | da Silva, Adriana Cristina Formoso and Serrão Pinto, Thiago | 2025 | Not a primary study design |
| 382 | ATENÇÃO FARMACÊUTICA PARA PACIENTES EM TRATAMENTO DA OBESIDADE: O USO DE MEDICAMENTOS OFF-LABEL E DA SEMAGLUTIDA (OZEMPIC®) COMO TRATAMENTO SECUNDÁRIO. | da Silva Gomes, Mizael and Serrão Pinto, Thiago | 2025 | Not a primary study design |
| 383 | ATUAÇÃO DO FARMACÊUTICO NO ACOMPANHAMENTO E ADESÃO AO TRATAMENTO DA TUBERCULOSE PULMONAR EM MANAUS. | Araújo Oliveira, Lidiane and Tufic Garutti, Samantha dos Santos | 2025 | Not a primary study design |
| 384 | ATUAÇÃO DO FARMACÊUTICO NA DIABETES MELLITUS GESTACIONAL. | de Sousa Figueira, Nesilda and dos Santos, Viviane Marinho | 2025 | Not a primary study design |
| 385 | A ATUAÇÃO FARMACÊUTICA NA DISPENSAÇÃO E ORIENTAÇÃO DA TERAPIA INSULÍNICA NA ATENÇÃO BÁSICA. | da Silva Andrade, Theo and Serrão Pinto, Thiago | 2025 | Not a primary study design |
| 386 | ATUAÇÃO DO FARMACÊUTICO NA PREVENÇÃO DO CÂNCER DO COLO DO ÚTERO: ESTRATÉGIAS EM SAÚDE E ADESÃO AO RASTREAMENTO NA ATENÇÃO BÁSICA E UPAS. | de Souza, Jordana Duarte and dos Santos, Viviane Marinho | 2025 | Not a primary study design |
| 387 | A IMPORTÂNCIA DA INSERÇÃO DO FARMACÊUTICO CLÍNICO NA EQUIPE MULTIPROFISSIONAL DA ATENÇÃO PRIMÁRIA À SAÚDE. | Maria, Thayse and Mota Batista, Tatianne and Ferreira Rodrigues, Rawany Renhya and Lourenço Lima, Sebastião Matheus and Abrantes Justino, Kássia Shamytha and de Fátima Silva, Lucivânia and Nogueira da Silva, Rafael and Nunes dos Santos, Amanda Margarida | 2025 | Not a primary study design |
| 388 | Users' Perspectives on Primary Care and Public Health Services in the State of Rio de Janeiro, Brazil: A Cross-Sectional Study with Implications for Healthcare Quality Assessment. | Ferraz, Igor F. L. and Raimundo, Mariana C. and Barros, Natalia M. A. M. and Souza, Jhoyce S. and Lucio, Barbará M. V. and Tenreiro, Thiago P. and Reis, Edna A. and de Souza Serio dos Santos, Danielle Maria and Chaves, Luisa A. and Godman, Brian and Campbell, Stephen M. and Meyer, Johanna C. and Godói, Isabella Piassi D. | 2025 | Did not describe a clinical pharmacy service |
| 389 | Avaliação da capacidade de gestão da assistência farmacêutica na Atenção Primária à Saúde dos municípios brasileiros. | Faraco, Emília Baierle and Rover, Marina Raijche Mattozo and Leite, Silvana Nair | 2025 | Did not describe a clinical pharmacy service |
| 390 | Os trabalhadores farmacêuticos na Atenção Básica de Porto Alegre-RS e a pandemia de coronavírus. | Carneiro Ramos, Diego and Araujo dos Santos Júnior, Genival and Rocha Ayres, Lorena and Degli Esposti, Carolina Dutra | 2023 | Did not describe a clinical pharmacy service |
| 391 | Atenção Farmacêutica em Farmácias e Drogarias: existe um processo de mudança? | Santos Júnior, Genival Araujo dos and Ramos, Sheila Feitosa and Pereira, André Mascarenhas and Dosea, Aline Santana and Araújo, Elton Matos and Onozato, Thelma and Pimentel, Déborah Mônica Machado and Jr.Lyra, Divaldo Pereira de | 2009 | Did not describe a clinical pharmacy service |
| 392 | Adoption of drones in pharmaceutical retail: barriers and facilitators according to the perception of pharmacists. | Farina, Simone Sena and Romano-Lieber, Nicolina Silvana | 2023 | Did not describe a clinical pharmacy service |
| 393 | Perfil e atuação dos farmacêuticos na rede pública de saúde do Município do Jaboatão dos Guararapes-PE. | Midori Oishi Nemoto, Miriam Christi and Maiochi Luiz, Gabriel and Contreras Pinochet, Luis Hernan and Hikaru Oishi, Pedro | 2021 | Did not describe a clinical pharmacy service |
| 394 | Interface entre a saúde mental de crianças e adolescentes e a atuação clínica do farmacêutico: um estudo qualitativo. | Sá, Jarbas Damasceno and Cruz, Isabely Vera and Randau, Karina Perrelli | 2022 | Not conducted in a Primary Health Care setting |
| 395 | Relato de Experiência do Estágio em Práticas Farmacêuticas. | Takasugui Damasceno, Luani and Jamile Mendes, Samara and Melo Aguiar, Patricia | 2025 | Did not describe a clinical pharmacy service |
| 396 | Detección de Problemas Relacionados con Medicamentos en Pacientes Adultos a través de Seguimiento Farmacoterapéutico. | de Miranda Silva, Quézia and Duarte dos Santos, Maria Eduarda and Sampaio Oliveira, Pedro Alexandre | 2025 | Not conducted in a Primary Health Care setting |
| 397 | Nível de satisfação dos usuários e verificação do conhecimento dos farmacêuticos em farmácias públicas do Espírito Santo, Brasil. | Laiber Bonadiman, Raphael and Fiorese Santanna, Adriélly and Alexandre Brasil, Girlandia and de Lima, Ewelyne Miranda and Lenz, Dominik and Coutinho Endringer, Denise and Uggere Andrade, Tadeu | 2018 | Did not describe a clinical pharmacy service |
| 398 | Análise da Busca Ativa em Exames de Contato de Casos de Hanseníase no Município de Almenara-MG entre 2017-2021. | Sousa Lopes, Rayllane and de Souza Nascimento, Ednardo and Toledo Coelho, Viviane Amaral and Coelho, Thomaz and Giselly de Souza, Carla and Santos Bigatello, Creonice and Souto de Araújo, Luanna Botelho | 2023 | No pharmacists involved |
| 399 | Prison pharmaceutical care in the State of Pará, Brazil: determining factors for access to medicines and the right to health. | Alves Monteiro, Anna Paula and Soler, Orenzio and Diogo de Queiroz, Luana Melo | 2022 | Not conducted in a Primary Health Care setting |
| 400 | Relato de experiência em estágio supervisionado em Farmácia de Manipulação e Comunitária. | Ferreira Santos, Winnicius Pereira and Rodrigues Ferreira, Kairo Batista and Arruda Braz, Carolina | 2025 | No pharmacists involved |
| 401 | Development and validation of an instrument to measure the professional's knowledge of dispensing medication (CDM-51) in community pharmacies. | Freato Gonçalves, Ana Maria Rosa and Almeida Campos, Marília Silveira and Bernardes, Andrea and Gabriel, Carmem Silvia and Pereira, Leonardo Régis Leira | 2020 | Not conducted in a Primary Health Care setting |
| 402 | CUIDADO ÀS MULHERES QUE VIVENCIAM A AMAMENTAÇÃO E A TERAPIA MEDICAMENTOSA NO PERÍODO PUERPERAL. | Argolo Teixeira, Marizete and de Carvalho Almeida, Bruna Rivelli and Lopes Costa, Elayny and dos Anjos Matos, Robson | 2019 | No pharmacists involved |
| 403 | Cost-effectiveness analysis of pharmaceutical care for hypertensive patients from the perspective of the public health system in Brazil. | Cazarim, Maurílio de Souza and Pereira, Leonardo Régis Leira | 2018 | Not conducted in a Primary Health Care setting |
| 404 | Avaliação da prescrição e perfil de utilização de medicamentos antimicrobianos pela rede pública municipal de saúde de Encanto-RN. | Chaves Lopes, Francisco Clébison and de Sousa Oliveira, Fernando | 2021 | Not a primary study design |
| 405 | Indicadores do Serviço de Atenção Farmacêutica (SAF) da Universidade do Sul de Santa Catarina. | Alano, Graziela Modolon and Corrêa, Taís dos Santos and Galato, Dayani | 2012 | Not conducted in a Primary Health Care setting |
| 406 | Pharmaceutical care for patients with persistent asthma: assessment of treatment compliance and use of inhaled medications. | Santos, Daiane de Oliveira and Martins, Maria Cleusa and Cipriano, Sonia Lucena and Carvalho Pinto, Regina Maria and Cukier, Alberto and Stelmach, Rafael | 2010 | Not conducted in a Primary Health Care setting |
| 407 | A pilot integrated qualification program to improve medication use in a long-term care facility: a hybrid effectiveness-implementation study. | dos Santos Oliveira Lima, Felipe Augusto and Gonzaga do Nascimento, Mariana Martins and de Paula Rezende, Cristiane and Dias dos Santos, Marina and Raid Farnese, Luciana and Dornellas Faria, Samilla and de Assis Santos, Aline Silva and Gonçalves Ferreira, Sabrina and Oliveira Buzelin Doria, Gabriela and Ramalho-de-Oliveira, Djenane | 2022 | Not conducted in a Primary Health Care setting |
| 408 | ANÁLISE ESTRUTURAL DA ATENÇÃO PRÉ-NATAL E DO SERVIÇO DE FARMÁCIA EM UMA REGIÃO DO MUNICÍPIO DE FORTALEZA - CE. | Soares Gondim, Ana Paula and de Menezes Martins, Charles Héverton Ferreira and de Lima, Mary Braga and Pinto Ferreira, Bruno Sousa and de Lima, Suzy Cléa Braga | 2011 | Did not describe a clinical pharmacy service |
| 409 | Assistência farmacêutica no cuidado à saúde na Atenção Primária: tão perto, tão longe. | Amano Maximo, Samuel and Andreazza, Rosemarie and de Oliveira Cecilio, Luiz Carlos | 2020 | Not a primary study design |
| 410 | CONHECIMENTO DE FARMACÊUTICOS SOBRE ALEITAMENTO MATERNO: UM ESTUDO NAS FARMÁCIAS COMERCIAIS EM FORTALEZA-CE. | Mesquita Moreira da Silva, Luzia Izabel and Pontes Thé, Patrícia Maria and de Oliveira Medeiros, Joana and Maia Oliveira Giacomini, Sâmia Graciele and de Cássia Barros Rodrigues, Rita and Lopes Vieira Peixoto, Maria Marly | 2012 | Did not describe a clinical pharmacy service |
| 411 | Pharmaceutical care and complementary and alternative medicine in the SUS: Knowledge and acceptance on the part of the population from the city of São João da Mata, Minas Gerais state, Brazil. | Marques, Luciene Alves Moreira and Ribeiro do Vale, Flávia Vanessa Vieira and Santos Nogueira, Valéria Aparecida dos and Mialhe, Fábio Luiz and Silva, Lara Cristina | 2011 | Did not describe a clinical pharmacy service |
| 412 | Satisfação dos pacientes com doença de Chagas atendidos por um serviço de atenção farmacêutica no estado do Ceará, Brasil. | da Costa, Alanna Carla and da Silva Cândido, Darlan and de Barros Vasconcelos Fidalgo, Arduina Sofia Ortet and da Silva Filho, José Damião and Menezes Viana, Carlos Eduardo and Lima, Monise Anne and Andrade, Mônica Coelho and de Fátima Oliveira, Maria | 2018 | Did not describe a clinical pharmacy service |
| 413 | Servicio de revisión del uso de medicamentos en farmacias comunitarias: una puerta abierta a otros servicios farmacéuticos clínicos. | Lavrador, Marta and Cabral, Ana C. and Lima, Tácio M. and Vitória Figueiredo, Isabel and Margarida Castel-Branco, Maria | 2025 | Not conducted in a Latin American country |
| 414 | MUTIRÃO DE COMBATE A DENGUE DENTRO DE UM TERRITÓRIO NA ATENÇÃO PRIMÁRIA EM SAÚDE. | de Lima, Nícolas Milhome and Carneiro, Rômulo Oliveira do Vale and Rocha, Andressa Carvalho and Damasceno, Flávio Marques | 2024 | Did not describe a clinical pharmacy service |
| 415 | Guidance provided by pharmacists to customers regarding to destination of unused household medications: disposal of household medications. | Michelin, Aparecida de Fatima and Bonifácio, Neuza Alves and Nagata, Walter Bertequini and da Silva, Valéria Maria Savoya and Gobbo, Laura Emilia Michelin and Bresciani, Katia Denise Saraiva | 2023 | Did not describe a clinical pharmacy service |
| 416 | Magnitude of the White-Coat Effect in the Community Pharmacy Setting: The MEPAFAR Study. | Sabater-Hernández, Daniel and de la Sierra, Alejandro and Sánchez-Villegas, Pablo and Baena, María I. and Amariles, Pedro and Faus, María J. | 2011 | No pharmacists involved |
| 417 | Community Pharmacists Identification of Gaps in Care – Statin Utilization for Primary Prevention. | White, Raechel T. and Sankey, Kjersten and Anderson, Joe R. and Bos, Alexander J. and Aragon, Kelsea Gallegos and Martinez-Nava, Diana D. and Rubio, Annajita M. and Turner, Robyn M. and Ray, Gretchen M. | 2024 | Did not describe a clinical pharmacy service |
| 418 | Improving pharmaceutical practice in diabetes care using point-of-care glycated haemoglobin testing in the community pharmacy. | Santana, Caroline Rocha and de Oliveira, Márcio Galvão Guimarães and Camargo, Marianne Silveira and Moreira, Pablo Maciel Brasil and de Castro, Priscila Ribeiro and Aguiar, Erlan Canguçu and Mistro, Sóstenes | 2024 | Insufficient data for extraction |
| 419 | Facilitators and strategies to implement clinical pharmacy services in a metropolis in Northeast Brazil: a qualitative approach. | Ramos, Sheila Feitosa and Santos Júnior, Genival Araujo dos and Pereira, André Mascarenhas and Dosea, Aline Santana and Rocha, Kérilin Stancine Santos and Pimentel, Déborah Mônica Machado and Lyra-Jr, Divaldo Pereira de and Santos Júnior, Genival Araujo Dos and Pereira, André Mascarenhas and Rocha, Kérilin Stancine Santos and Pimentel, Déborah Mônica Machado | 2018 | Did not describe a clinical pharmacy service |
| 420 | Clinical Impact of a Comprehensive Medication Management Service in Primary Health Care. | Santos, Bruna Damázio and Nascimento, Mariana Martins Gonzaga do and de Oliveira, Grazielli Cristina Batista and Nascimento, Yone de Almeida and Mambrini, Juliana Vaz de Melo and Cid, Annaline Stiegert and Piovesan, Taíza Graziele Costa and Fernandes, Laura Barbosa and Martins, Ursula Carolina de Morais and Neves, Carina de Morais and da Silva, Danielle Fernandes and de Oliveira, Djenane Ramalho | 2021 | Insufficient data for extraction |
| 421 | Assessment of community pharmacists' counselling skills on headache management by using the simulated patient approach: a pilot study. | Santos, Ana P. and Mesquita, Alessandra R. and Oliveira, Karolline S. and LYRA Jr., Divaldo P. | 2013 | Did not describe a clinical pharmacy service |
| 422 | Quality of prescription writing in Brazilian primary health care | Batista | 2023 | No pharmacists involved |
| 423 | International Latin American Survey on Pediatric Intestinal Failure Team | Spolidoro | 2021 | Not conducted in a Primary Health Care setting |
| 424 | Impact of a Pharmacy Management Course for Pharmacists Working Within Brazil’s Public Health System | Manzini | 2021 | Did not describe a clinical pharmacy service |
| 425 | Pharmaceutical Care in Community Pharmacies: Practice and Research in Peru | Alvarez-Risco | 2007 | Not a primary study design |
| 426 | Polypharmacy in older patients at primary care units in Brazil | Gomes | 2019 | No pharmacists involved |
| 427 | Utilização de medicamentos antes e durante a gestação: prevalência e fatores associados | Costa | 2017 | No pharmacists involved |
| 428 | Patterns of antimicrobial use and antimicrobial resistance among healthy children in Bolivia | Bartoloni | 1998 | Not conducted in a Primary Health Care setting |
| 429 | Potentially inappropriate prescribing and associated factors in elderly patients at hospital discharge in Brazil: a cross-sectional study | Mori | 2017 | Not conducted in a Primary Health Care setting |
| 430 | Prescribing omissions among elderly Brazilian patients at their hospital admission and discharge: cross‐sectional study | Luz | 2018 | No pharmacists involved |
| 431 | Survey of calcium supplementation to prevent preeclampsia: the gap between evidence and practice in Brazil | Camargo | 2013 | No pharmacists involved |
| 432 | Effect of a Social Norm Email Feedback Program on the Unnecessary Prescription of Nimodipine in Ambulatory Care of Older Adults A Randomized Clinical Trial | Torrente | 2020 | Not conducted in a Primary Health Care setting |
| 433 | Bridging the mental health treatment gap: effects of a collaborative care intervention (matrix support) in the detection and treatment of mental disorders in a Brazilian city | Saraiva | 2020 | No pharmacists involved |
| 434 | Potential prescription patterns and errors in elderly adult patients attending public primary health care centers in Mexico City | Corona-Rojo | 2019 | No pharmacists involved |
| 435 | Prescrição de antidepressivos na atenção primária: um estudo descritivo acerca da confiança dos profissionais médicos | Moreno | 2024 | No pharmacists involved |
| 436 | PARAGUAYAN PHARMACIES AND THE SALE OF PSEUDO-ABORTIFACIENTS | Oddone | 1991 | Not conducted in a Primary Health Care setting |
| 437 | Buenas prácticas de dispensación de antibióticos en farmacias comunitarias de la provincia de Córdoba, Argentina | Armando | 2025 | Did not describe a clinical pharmacy service |
| 438 | Mobile phone text messaging improves antihypertensive drug adherence in the community | Varleta | 2017 | No pharmacists involved |
| 439 | Evaluation of negative outcomes associated with medication (NOM) by pharmacists at a home assistance programme in a Brazilian teaching hospital | Takahashi | 2011 | No pharmacists involved |
| 440 | Perspectives of healthcare professionals on training for quantitative G6PD testing during implementation of tafenoquine in Brazil (QualiTRuST Study) | Santos | 2024 | No pharmacists involved |
| 441 | Knowledge of prescribed drugs among primary care patients: findings from Prover Project | Passagli | 2021 | No pharmacists involved |
| 442 | Descrição do consumo de psicofármacos na atenção primária à saúde de Ribeirão Preto, São Paulo, Brasil | Oliveira | 2020 | No pharmacists involved |
| 443 | The effect of probiotics on functional constipation in adults | Miltemão; Mitelmão | 2020 | Not conducted in a Primary Health Care setting |
| 444 | Effectiveness of Kuska Tele-Wasi, a home blood-pressure telemonitoring system for hypertension control in Lima, Peru: a randomised controlled trial | Calderon-Anyosa | 2019 | No pharmacists involved |
| 445 | Potentially Inappropriate Medications in Elderly Ambulatory Patients: A Comparative Study between a Primary Health Care Center and a Community Pharmacy | Chiapella | 2018 | No pharmacists involved |
| 446 | An Educational Intervention to Improve Effectiveness in the Detection, Treatment and Control of patients with high cardiovascular risk in low-resource settings in Argentina: Rationale and Study Design of a cluster randomized controlled trial. | Gulayin | 2017 | No pharmacists involved |
| 447 | Healthcare seeking behavior in Individuals with Influenza-like Illness (ILI) during the influenza pandemic of 2009 compared to posterior years in Peru | Tapia | 2014 | Did not describe a clinical pharmacy service |
| 448 | Implementing the Cuban healthcare system in underserved areas to improve access to care: “flowers in the desert” | Rojas | 2023 | No pharmacists involved |
| 449 | Potential Drug-Drug Interactions in Prescriptions to Patients over 45 Years of Age in Primary Care, Southern Brazil | Teixeira; Texeira | 2012 | No pharmacists involved |
| 450 | FRECUENCIA, TIPOS, CAUSAS Y COSTOS RELACIONADOS CON LOS ERRORES DE MEDICACIÓN | Garcia | 2011 | No pharmacists involved |
| 451 | Farmacêutico na farmácia básica: garantia de qualidade na assistência farmacêutica? | Reis | 2020 | Did not describe a clinical pharmacy service |
| 452 | Compreendendo os significados das interações entre profissionais de saúde e idosos usuários de medicamentos | Júnior; Junior | 2008 | Did not describe a clinical pharmacy service |
| 453 | Preventing and managing falls: an international online survey | Herzog | 2022 | Not conducted in a Primary Health Care setting |
| 454 | FATORES RELACIONADOS À ADESÃO FARMACOTERAPÊUTICA DE PACIENTES HIPERTENSOS ACOLHIDOS NA ESTRATÉGIA DE SAÚDE DA FAMÍLIA | Ferreira | 2013 | No pharmacists involved |
| 455 | Pharmaceutical services in primary health care: dissatisfaction among users, managers and pharmacists | Cruz | 2022 | Did not describe a clinical pharmacy service |
| 456 | ACEITAÇÃO À TERAPIA FARMACOLÓGICA ENVOLVENDO O USO DEMEDICAMENTOS GENÉRICOS POR IDOSOS | Alves | 2025 | No pharmacists involved |
| 457 | Educação permanente para farmacêuticos preceptores que atuam na atenção primária no Sistema Único de Saúde: um estudo qualitativo | Bonomo | 2023 | No pharmacists involved |
| 458 | Compreensão de território nos serviços farmacêuticos da atenção básica à saúde: um estudo qualitativo | Loss | 2022 | Did not describe a clinical pharmacy service |
| 459 | Acolhimento na Estratégia Saúde da Família em uma cidade do norte de Minas Gerais: um estudo descritivo, 2019-2020 | Barbosa | 2022 | Did not describe a clinical pharmacy service |
| 460 | Percepção de farmacêuticos na implantação do Cuidado Farmacêutico na Atenção Básica | D'Andréa; D'Andreia | 2022 | Did not describe a clinical pharmacy service |
| 461 | Child health care in primary care in a border region | Picco | 2022 | No pharmacists involved |
| 462 | CAPACITAÇÃO DE PROFISSIONAIS NA ATENÇÃO PRIMÁRIA EM SAÚDE: UM CAMINHO PARA A PROMOÇÃO DA FITOTERAPIA | Zeni | 2021 | No pharmacists involved |
| 463 | Caracterização do acesso a medicamentos em um município de médio porte no leste do estado de Minas Gerais, Brasil | Gaede; Gaedie | 2020 | No pharmacists involved |
| 464 | DESEMPENHO DOS SERVIÇOS DA ATENÇÃO PRIMÁRIA À SAÚDE: SATISFAÇÃO DAS PESSOAS COM HIPERTENSÃO1 | Lucena | 2021 | No pharmacists involved |
| 465 | Assistência à saúde mental no Brasil: estudo multifacetado em quatro grandes cidades | Amaral | 2021 | Not conducted in a Primary Health Care setting |
| 466 | Conditions that interfere in the care of women in situation of conjugal violence | Carneiro | 2021 | Not conducted in a Primary Health Care setting |
| 467 | Health financing in Brazil and the goals of the 2030 Agenda: high risk of failure | Vieira | 2020 | Not conducted in a Primary Health Care setting |
| 468 | Protocol for hypertensive individuals assisted in Basic Health Care | DAntas; Dantas | 2019 | No pharmacists involved |
| 469 | Uso e abuso de benzodiazepínicos na atenção primária à saúde: práticas profissionais no Brasil e em Cuba | Fegadolli | 2019 | No pharmacists involved |
| 470 | MUSCLE ADVERSE EVENTS AMONG USERS OF SIMVASTATIN IN PRIMARY HEALTH CARE: COLLABORATIVE DRUG THERAPY MANAGEMENT | Lobrigatte | 2018 | Did not describe a clinical pharmacy service |
| 471 | PROMOÇÃO DE SAÚDE BUCAL NAS CONSULTAS DE CRESCIMENTO E DESENVOLVIMENTO NA ATENÇÃO PRIMÁRIA: UM RELATO DE COLABORAÇÃO INTERPROFISSIONAL | Araujo | 2018 | Did not describe a clinical pharmacy service |
| 472 | Users’ Perceptions of Access to and Quality of Public Health Services in Brazil: A Cross-Sectional Study in Metropolitan Rio de Janeiro, Including Pharmaceutical Services | Raimundo | 2025 | Did not describe a clinical pharmacy service |
| 473 | Pharmacological Management of Acute and Chronic Insomnia: A Cross-Sectional Study | Valladales-Restrepo; Valladales Restrepo | 2024 | No pharmacists involved |
| 474 | Resident pharmacist participation in shared medical appointments in palliative care in São Paulo, Brazil: experience and contributions | Silva; da Silva | 2024 | Not conducted in a Primary Health Care setting |
| 475 | Manifestações de violência obstétrica percebidas por gestantes na assistência ao pré-natal na Atenção Primária à Saúde | Ferreira | 2024 | No pharmacists involved |
| 476 | SEGURANÇA DO PACIENTE NA ATENÇÃO PRIMÁRIA: PERCEPÇÃO DOS PROFISSIONAIS | Carvalho | 2024 | Did not describe a clinical pharmacy service |
| 477 | Professionals’ and Patients’ Perspectives on Criteria for Referring Hypertensive Patients to Comprehensive Medication Management Services in Public Primary Health Care | Sousa | 2023 | Did not describe a clinical pharmacy service |
| 478 | Exposure to intimate partner violence and lack of asthma control in adults: a cross-sectional study | Lima | 2023 | Not conducted in a Primary Health Care setting |
| 479 | MedMinas project: design and use of mixed methods in the evaluation of pharmaceutical services in primary health care in Minas Gerais, Brazil | Luz | 2022 | No pharmacists involved |
| 480 | Capacitação e intervenções de técnicos de farmáciana dispensação de medicamentos em Atenção Primária à Saúde | Melo | 2017 | No pharmacists involved |
| 481 | Therapeutic communication in the interaction between health workers and hypertensive patients in the family health strategy | Torres | 2017 | No pharmacists involved |
| 482 | NEEDS AND OPPORTUNITIES FOR OUTPATIENT PHARMACIES IN COLOMBIA: THE CASE OF CONTINUING EDUCATION PROGRAMS FOR PHARMACISTS | Ceballo | 2017 | Not conducted in a Primary Health Care setting |
| 483 | Comunicação terapêutica na interação profissional de saúde e hipertenso na estratégia saúde da família | Torres | 2017 | No pharmacists involved |
| 484 | Compreensão da prescrição de medicamentos em uma unidade básica de saúde: autorrelato do usuário e aferição pelo pesquisador | Coradis; Coradi | 2016 | No pharmacists involved |
| 485 | PREVALÊNCIA DE PRESCRIÇÃO DE MEDICAMENTOS POTENCIALMENTE INAPROPRIADOS PARA IDOSOS EM UMA UNIDADE DE SAÚDE DA FAMÍLIA DE PORTO ALEGRE/RS | Bueno | 2016 | No pharmacists involved |
| 486 | Effectiveness of pharmaceutical care for drug treatment adherence in patients with systemic lupus erythematosus in Rio de Janeiro, Brazil: study protocol for a randomized controlled trial | Oliveira-Santos | 2016 | Not conducted in a Primary Health Care setting |
| 487 | Consumption of medicinal plants by patients with heart diseases at a pharmacist-managed anticoagulation clinic in Brazil | Leite | 2016 | Not conducted in a Primary Health Care setting |
| 488 | DESFECHOS DOS ENCAMINHAMENTOS DOS USUÁRIOS DA ATENÇÃO PRIMÁRIA À SAÚDE À FARMÁCIA DE MEDICAMENTOS ESPECIAIS DO ESTADO RIO GRANDE DO SUL | Barros | 2016 | Not conducted in a Primary Health Care setting |
| 489 | Clinical pharmaceutical services of primary health care of the Federal District: A discussion based on the SWOT matrix | Barros | 2022 | Did not describe a clinical pharmacy service |
| 490 | User embracement in the Family Health Strategy in a city in the north of Minas Gerais, Brazil: a descriptive study, 2019-2020 | Barbosa | 2022 | No pharmacists involved |
| 491 | Uso de plantas medicinais por usuários do serviço público de saúde do município de Ijuí/RS | Colet | 2015 | No pharmacists involved |
| 492 | Elenco de medicamentos do Programa Farmácia Popular do Brasil e a Política de Nacional Assistência Farmacêutica | Miyuki-Yamauti; Yamauti | 2025 | No pharmacists involved |
| 493 | Access to continued-use medication among older adults, Brazil | Viana | 2015 | Not conducted in a Primary Health Care setting |
| 494 | Primary health care in CSP | Almeida | 2022 | No pharmacists involved |
| 495 | Access to medicines in the Brazilian Unified Health System’s primary health care: assessment of a public policy | Barbosa | 2021 | No pharmacists involved |
| 496 | Adaptación y validación de instrumentos para medir errores en las recetas médicas y en el proceso de preparación de medicamentos en farmacia de pacientes ambulatorios | Alvarado | 2014 | Not conducted in a Primary Health Care setting |
| 497 | Implementation of clinical pharmacy services using problematization with Maguerez Arc: A quasi-experimental before-after study | Júnior | 2021 | Not conducted in a Primary Health Care setting |
| 498 | Physician and patient-related factors associated with inappropriate prescribing to older patients within primary care: a cross-sectional study in Brazil | Amorim | 2021 | No pharmacists involved |
| 499 | Strategies to improve the availability of medicines in primary health care in Brazil: findings and implications | Barbosa | 2021 | No pharmacists involved |
| 500 | Influência dos gastos em saúde no empobrecimento de domicílios no Brasil | Boing | 2014 | Not conducted in a Primary Health Care setting |
| 501 | Understanding the provision of a clinical service in mental health and the role of the pharmacist: a qualitative analysis | Fernandes | 2021 | Not conducted in a Primary Health Care setting |
| 502 | Descarte de medicamentos: uma análise da prática no Programa Saúde da Família | Alencar | 2014 | No pharmacists involved |
| 503 | “Kit-covid” and the Popular Pharmacy Program in Brazil | Santos-Pinto | 2021 | No pharmacists involved |
| 504 | Development and Implementation of a Decision Support System to Improve Control of Hypertension and Diabetes in a Resource-Constrained Area in Brazil: Mixed Methods Study | Marcolino | 2021 | No pharmacists involved |
| 505 | Avaliação da farmacoterapia na doença renal crônica: validação do instrumento PAIR para uso no Brasil | Marquito | 2020 | Not conducted in a Primary Health Care setting |
| 506 | Os impactos da judicialização da saúde no município de São Paulo: gasto público e organização federativa1 | Wang | 2014 | Not conducted in a Primary Health Care setting |
| 507 | Avaliação da farmácia caseira no território de Unidades de Saúde de um Serviço de Atenção Primária | Costa | 2014 | No pharmacists involved |
| 508 | Monitoring of Delay to Pharmacy Refill in Assessing Adherence to Antiretroviral Therapy | Cruz | 2020 | No pharmacists involved |
| 509 | Papel dos medicamentos nas intoxicações causadas por agentes químicos em município da Bahia, no período de 2007 a 2010 | Teles | 2013 | No pharmacists involved |
| 510 | ANÁLISE DO ACESSO A MEDICAMENTOS EM UMA UNIDADE DO PROGRAMA DE SAÚDE DA FAMÍLIA EM PORTO ALEGRE | Bueno | 2013 | Did not describe a clinical pharmacy service |
| 511 | Development and validation of key performance indicators for medication management services provided for outpatients | Lima | 2019 | No pharmacists involved |
| 512 | Potentially inappropriate prescribing to older adults in ambulatory care: prevalence and associated patient conditions | Mino‐León | 2019 | No pharmacists involved |
| 513 | Assistência Farmacêutica na atenção primária à saúde de um Município Potiguar, Brasil | Bernadino | 2019 | No pharmacists involved |
| 514 | Revisiting Concepts, Attitudes and Expectations of Brazilian Pharmacists to the Practice of Pharmaceutical Care: A Qualitative Perspective | Fegadolli | 2018 | Did not describe a clinical pharmacy service |
| 515 | Assessment of oral anticoagulation control at two pharmacist-managed clinics in Brazil | Silva | 2017 | Not conducted in a Primary Health Care setting |
| 516 | Coste-efectividad del seguimiento farmacoterapéutico en pacientes con trastorno afectivo bipolar-I: ensayo clínico aleatorizado EMDADER-TAB | David | 2017 | Not conducted in a Primary Health Care setting |
| 517 | Use of medicinal plants by health professionals in Mexico | Alonso-Castro | 2017 | No pharmacists involved |
| 518 | Impact of a medication therapy management service on the clinical status of patients with chronic obstructive pulmonary disease | Detoni | 2017 | Not conducted in a Primary Health Care setting |
| 519 | Acesso a medicamentos: relações com a institucionalização da assistência farmacêutica | Barros | 2017 | Did not describe a clinical pharmacy service |
| 520 | Pesquisa Nacional sobre Acesso, Utilização e Promoção do Uso Racional de Medicamentos: métodos | Álvares; Alvares | 2017 | No pharmacists involved |
| 521 | Direito à saúde e judicialização de medicamentos: a experiência de Niterói | Barreira | 2012 | Not conducted in a Primary Health Care setting |
| 522 | Suicide Prevention Training for Professionals in the Public Health Network in a Large Brazilian City | Cais | 2011 | No pharmacists involved |
| 523 | Impacto da atenção farmacêutica no uso racional de antimicrobianos em uma unidade básica de saúde no interior do 'Estado de São Paulo | Neto; Obreli-Neto | 2011 | No pharmacists involved |
| 524 | AVALIAÇÃO DA ASSISTÊNCIA FARMACÊUTICA BÁSICA EM UM MUNICÍPIO DA BAHIA, BRASIL | Oliveira | 2010 | No pharmacists involved |
| 525 | Costo oportunidad de adolescentes en unidades de medicina familar | Ugalde | 2010 | No pharmacists involved |
| 526 | Pesquisa Nacional sobre Acesso, Utilização e Promoção do Uso Racional de Medicamentos (PNAUM): avaliação dos serviços de atenção farmacêutica primária | Akerman | 2017 | Did not describe a clinical pharmacy service |
| 527 | Evaluation of Prescriptions and Dispensing of Medicines in a Basic Health Unit In Belo Horizonte, Minas Gerais, Brazil | Coradi | 2017 | No pharmacists involved |
| 528 | Pharmaceutical Care in Hypertension Patients in a Peruvian Hospital | Alvarez-Risco | 2016 | Not conducted in a Primary Health Care setting |
| 529 | Frequency of prescriptions of off-label drugs and drugs not approved for pediatric use in primary health care in a southern municipality of Brazil | Gonçalves | 2016 | No pharmacists involved |
| 530 | Prevalence, symptom patterns and management of episodic diarrhoea in the community: a population-based survey in 11 countries | Hungin | 2016 | No pharmacists involved |
| 531 | Compreensão da prescrição de medicamentos em uma unidade básica de saúde: autorrelato do usuário e aferiçãopelo pesquisador | Coradi | 2016 | No pharmacists involved |
| 532 | Medication regimen complexity in adults and the elderly in a primary healthcare setting: determination of high and low complexities | Ferreira | 2015 | No pharmacists involved |
| 533 | Effectiveness of pharmaceutical care at discharge in the emergency department: study protocol of a randomized controlled trial | Huhmmer; Kuhmmer | 2015 | Not conducted in a Primary Health Care setting |
| 534 | Prescription errors as a barrier to pharmaceutical care in public health facilities: Experience Peru | Álvarez-Risco | 2015 | No pharmacists involved |
| 535 | Economic Evaluation of a Pharmaceutical Care Program for Elderly Diabetic and Hypertensive Patients in Primary Health Care: A 36-Month Randomized Controlled Clinical Trial | Obreli-Neto | 2015 | Did not describe a clinical pharmacy service |
| 536 | Individualized homeopathic treatment of dermatological complaints in a public outpatient clinic | Waisse-Priven | 2009 | Not conducted in a Primary Health Care setting |
| 537 | Gasto do Ministério da Saúde com medicamentos: tendência dos programas de 2002 a 2007 | Vieira | 2009 | Not conducted in a Primary Health Care setting |
| 538 | Lecciones aprendidas en la provisión de medicamentos para la atención primaria de la salud | Tobar | 2008 | Not conducted in a Primary Health Care setting |
| 539 | New drugs in Brazil: Do they meet Brazilian public health needs? | Vidotti | 2008 | Not conducted in a Primary Health Care setting |
| 540 | Avaliação da assistência farmacêutica à gestante na rede básica de saúde do Município de Praia Grande, São Paulo, Brasil | Vireira | 2008 | Did not describe a clinical pharmacy service |
| 541 | Consejos farmacéuticos a pacientes con trastornos de humor | Reis | 2013 | Not conducted in a Primary Health Care setting |
| 542 | Pharmaceutical assistance in local public health services in Santa Catarina (Brazil): Characteristics of its organization | Veber | 2011 | Not conducted in a Primary Health Care setting |
| 543 | Utilización de Medicamentos en un Programa de Atención Primaria de Salud para Poblaciones Rurales Marginales | Gualda | 2009 | Not a primary study design |
| 544 | Demanda del servicio de atención farmacéutica en una farmacia comunitaria privada | Pires | 2006 | No pharmacists involved |
| 545 | Barriers to pharmaceutical care in Argentina | Uema | 2008 | Not conducted in a Primary Health Care setting |
| 546 | A relação entre a indústria farmacêutica e a assistência farmacêutica no âmbito do Sistema Único de Saúde (SUS): a compra estadual e municipal de medicamentos no Rio de Janeiro | Zaire | 2008 | Not conducted in a Primary Health Care setting |
| 547 | ANÁLISE DE PRESCRIÇÕES MEDICAMENTOSAS DISPENSADAS NA FARMÁCIA DE UMA UNIDADE BÁSICA DE SAÚDE DE PORTO ALEGRE - RS | Guzatto | 2007 | No pharmacists involved |
| 548 | Utilização de fitoterápicos nas unidades básicas de atenção à saúde da família no município de Maracanaú (CE) | Silva | 2006 | No pharmacists involved |
| 549 | Frequent Nurse Visits Decrease White Coat Effect in Stage III Hypertension | Guerra-Riccio | 2004 | No pharmacists involved |
| 550 | Prescripción médica y adherencia al tratamiento en diarrea infecciosa aguda: impacto indirecto de una interveción educativa | Bronfman | 1991 | Not conducted in a Primary Health Care setting |
| 551 | Evaluation of the impact of a pharmaceutical care program in children wisth asthma | González-Martin; Gonzalez-Martin | 2003 | Not conducted in a Primary Health Care setting |
| 552 | The pharmacist in the decentralization of health-care system: Pharmaceutical legislation analysis and diagnosis in the Province of Cordoba, Argentina | Uema | 2000 | Did not describe a clinical pharmacy service |
| 553 | Community pharmacist interventions in ear health: a scoping review | Taylor | 2021 | Not a primary study design |
| 554 | Perceptions of a group of hospital pharmacists and other professionals of the implementation of clinical pharmacy at a high complexity public hospital in Brazil | Alcântara; Alcantara | 2018 | Not conducted in a Primary Health Care setting |
| 555 | Serviços Farmacêuticos na Atenção Primária em Saúde à luz do modelo ambiguidade-conflito | Caetano | 2020 | No pharmacists involved |
| 556 | Medication adherence in patients with juvenile idiopathic arthritis | Adriano | 2017 | Not conducted in a Primary Health Care setting |
| 557 | Necessidades de saúde e masculinidades: atenção primária no cuidado aos homens | Schraiber | 2010 | No pharmacists involved |
| 558 | Higher education and health care in Brazil | Alemeida-Filho; Almeida-Filho | 2011 | No pharmacists involved |
| 559 | Costo de oportunidad en hombres que acuden a las unidades de medicina familiar en la ciudad de Querétaro, México | Carranza | 2010 | No pharmacists involved |
| 560 | Use of medicines before and during pregnancy: prevalence and associated factors | Costa | 2017 | No pharmacists involved |
| 561 | Prescribing omissions among elderly Brazilian at their hospital admission and discharge: cross‐sectional study | Luz | 2018 | Not conducted in a Primary Health Care setting |
| 562 | Capacitação e intervenções de técnicos de farmácia na dispensação de medicamentos em Atenção Primária à Saúde | Melo | 2017 | No pharmacists involved |
| 563 | Physician and patient-related factors associated withinappropriate prescribing to older patients within primary care: a cross-sectional study in Brazil | Amorim | 2021 | No pharmacists involved |
| 564 | Errores de prescripción como barrera para la Atención Farmacéutica en establecimientos de salud públicos: Experiencia Perú | Alvarez-Risco | 2015 | No pharmacists involved |
| 565 | Avaliação da assistência farmacêutica à gestantena rede básica de saúde do Município de Praia Grande, São Paulo, Brasil | Vieira | 2008 | Did not describe a clinical pharmacy service |
| 566 | Patients with HIV Support Clinic-Based Contraceptive Care by Pharmacists. |  |  | Insufficient data for extraction |
