# Supplementary material for: Clinical Services Provided by Pharmacists in Primary Health Care in Latin America: A Scoping Review
Source: J Eval Clin Pract. 2026 Nov 4;32(8):e70628. doi: 10.1111/jep.70628 (PMC13734460; doi:10.1111/jep.70628)
Supplement: Supplementary file 4 [file JEP-32-0-s004.docx]

**Supplementary Material 3**

| **Study ID** | **Author year** | **Country** | **Practice setting** | **Other setting specification** | **Study design** | **Study period** | **Original service label as reported** | **Standardized service classification** | **Theoretical framework cited** | **Methods or tools used** | **Team integration model** | **Outcome domains** | **Sample size as reported** | **Study population** | **Age group** | **Age interval** | **PROGRESS reporting** | **Equity dimension reported** |
| --- | --- | --- | --- | --- | --- | --- | --- | --- | --- | --- | --- | --- | --- | --- | --- | --- | --- | --- |
| 6 | Ceballo,2021 | Colombia | Community pharmacy | Privada | Cross-sectional study | abril/2018 - junho/2018 | Dispensação de medicamentos | Clinically oriented dispensing | Não citado explicitamente | Simulação / Role-play | Isolated: independent practice. | Service/process or management indicators | 305 drogarias e farmácias / 20 pacientes simulados | General population | Adults | média 20 anos | Not reported | Not reported |
| 10 | Cerqueira‐Santos, 2023 | Brazil | Community pharmacy | Privada | Qualitative study | novembro/2019 - janeiro/2020 | Skills and practices of pharmacy staff for dispensing of drugs | Clinically oriented dispensing | Não citado explicitamente | Protocolo Próprio / Desenvolvido pelos Autores | Isolated: independent practice. | Service/process or management indicators | 18 farmácias / 18 farmacêuticos | General population | Adults | Não informado | Not reported | Not reported |
| 11 | Dosea,2017 | Brazil | Community pharmacy | Privada | Qualitative study | 2012 - 2014 | Establishment, Implementation, and Consolidation of Clinical Pharmacy Services in Community Pharmacies | Clinically oriented dispensing; Health education; Therapeutic drug monitoring; Medication review | Não citado explicitamente | Não descrito / Não aplicável. | Isolated: independent practice. | Service/process or management indicators | 3 farmácias / 11 farmacêuticos | General population | Not reported | Não informado | Not reported | Not reported |
| 16 | Traverso,2007 | Argentina | Community pharmacy | Não especificado | Qualitative study | abril/2002 - agosto 2004 | Questionnaire to assess patient satisfaction with pharmaceutical care in Spanish language | Health education; Medication review | Não citado explicitamente | Protocolo Próprio / Desenvolvido pelos Autores | Isolated: independent practice. | Humanistic outcomes | 41 farmácias / 274 questionários obtidos | General population | Adults | 21 anos ou mais | Eligibility criterion | Age; Place of residence |
| 20 | Obreli-Neto,2013 | Brazil | Community pharmacy | Privada | Cross-sectional study | junho/2012 - outubro/2012 | Contraceptive Dispensing Practices of Community Pharmacists | Clinically oriented dispensing | Não citado explicitamente | Simulação / Role-play | Isolated: independent practice. | Service/process or management indicators | 4 pacientes simulados / 185 farmácias | General population | Adults | 20 - 25 anos | Target population/context description; Eligibility criterion | Sex/Gender; Place of residence |
| 21 | Turner, 2003 | Mexico | Community pharmacy | Privada | Mixed-methods study | agosto 1999 | Diagnosis and treatment of presumed STIs at Mexican pharmacies | Health screening; Clinically oriented dispensing | Não citado explicitamente | Simulação / Role-play; Protocolo Próprio / Desenvolvido pelos Autores | Isolated: independent practice. | Clinical outcomes; Service/process or management indicators | Em torno de 57 farmácias | General population | Adults | Média 32 anos | Not reported | Not reported |
| 22 | Bonadiman,2018; Bonadiman,2019 | Brazil | Community pharmacy | Pública | Mixed-methods study | maio-2013 - agosto/2013; maio-2013 - agosto/2014 | Nível de satisfação dos usuários e verificação do conhecimento dos farmacêuticos | Clinically oriented dispensing | Assistência Farmacêutica (Política Brasileira) | Satisfação); Protocolo Próprio / Desenvolvido pelos Autores | Isolated: independent practice. | Humanistic outcomes; Service/process or management indicators | 11 farmacêuticos / 8 farmácias / 294 pacientes; 12 farmacêuticos / 8 farmácias / 294 pacientes | General population | Adults | > 18 anos | Eligibility criterion | Education |
| 24 | Armando,2005 | Argentina | Community pharmacy | Pública | Mixed-methods study | Não mencionado | Monitoramento farmacoterapêutico | Pharmacotherapeutic follow-up | Método Dáder / Seguimento (Escola de Granada) | Método Dáder / Seguimento de Granada | Collaborative: shared case discussion and decision-making. | Service/process or management indicators | 38 farmacêuticos / 108 pacientes | General population | Mixed | 10 - 89 anos | Not reported | Not reported |
| 30 | Bailey,1974 | Colombia | Community pharmacy | Privada | Randomized controlled trial | novembro/1972 | Distribuição de folhetos sobre contraceptivos | Health education | Não citado explicitamente | Protocolo Próprio / Desenvolvido pelos Autores | Isolated: independent practice. | Economic outcomes | 66 farmácias | General population | Not reported | Não informaod | Not reported | Not reported |
| 35 | Antunes,2021 | Brazil | Basic health unit | - | Qualitative study | 03/09/20219 - 22/10/2019 | Programa educativo para a promoção da saúde de pacientes | Health education | Assistência Farmacêutica (Política Brasileira) | Protocolo Próprio / Desenvolvido pelos Autores | Collaborative: shared case discussion and decision-making. | Humanistic outcomes | 12 pacientes / 10 profissionais da saúde | Patients with polypharmacy | Mixed | Ensino fundamental completo | Eligibility criterion | Education |
| 37 | Souza,2020; Souza,2021 | Brazil | Basic health unit | - | Non-randomized clinical trial / quasi-experimental study | 03/2017 - 12/2019; 03/2017 - 12/2020 | Atuação do farmacêutico no controle da hipertensão em hipertensos | Minor ailment management; Health education | Assistência Farmacêutica (Política Brasileira) | Não descrito / Não aplicável. | Integrated/transdisciplinary: joint care or real-time shared health record. | Clinical outcomes | 30 pacientes / 23 farmacêuticos; 31 pacientes / 23 farmacêuticos | Patients with hypertension | Older adults | >60 anos | Target population/context description | Age |
| 41 | Canguçu, 2019 | Brazil | Community pharmacy | Pública | Randomized controlled trial | 01/2021 - 04/2022 | adherence to medication treatment and the reduction of blood pressure in patients with hypertension. | Health education; Therapeutic drug monitoring | Não citado explicitamente | Protocolo Próprio / Desenvolvido pelos Autores; Escalas de Adesão (Morisky; Protocolo Clínico Institucional / Ministério da Saúde | Isolated: independent practice. | Clinical outcomes; Humanistic outcomes | 157 indivíduos | Patients with hypertension | Adults | 30 - 69 anos | Eligibility criterion | Socioeconomic status; Education; Disability |
| 44 | Sánchez-Guerra, 2018; Sánchez-Guerra, 2019; Sánchez-Guerra, 2020; Sánchez-Guerra, 2021 | Mexico | Basic health unit | - | Randomized controlled trial | 10/2015 - 03/2016 | programa piloto de acompanhamento farmacoterapêutico | Health education; Pharmacotherapeutic follow-up; Therapeutic drug monitoring; Medication reconciliation | Método Dáder / Seguimento (Escola de Granada) | Método Dáder / Seguimento de Granada; Escalas de Adesão (Morisky | Integrated/transdisciplinary: joint care or real-time shared health record. | Clinical outcomes; Humanistic outcomes; Service/process or management indicators | 31 pacientes | Patients with hypertension | Adults | > 18 anos | Not reported | Not reported |
| 45 | Parody,2018; Parody,2019; Parody,2020 | Colombia | Basic health unit | - | Cross-sectional study | junho - novembro/2015 | Intervenção farmacêutica em pacientes com artrit \| Intervenção farmacêutica em pacientes com artrite | Pharmacotherapeutic follow-up; Medication review; Health education | Método Dáder / Seguimento (Escola de Granada) | Método Dáder / Seguimento de Granada; Escalas de Adesão (Morisky | Integrated/transdisciplinary: joint care or real-time shared health record. | Service/process or management indicators; Humanistic outcomes | 40 pacientes | Patients with autoimmune diseases | Adults | maior ou igual a 18 anos | Eligibility criterion | Disability |
| 50A | Oliveira,2017 | Brazil | Basic health unit | - | Qualitative study | 06/2013 - 08/2015 | implementação de serviços de gestão integral da medicação. | Pharmacotherapeutic follow-up | CMM - Gestão Integral da Farmacoterapia | CMM / GTM | Collaborative: shared case discussion and decision-making. | Service/process or management indicators | 10 farmacêuticos | General population | Adults | Não mencionado | Not reported | Not reported |
| 56 | Mardones,2020; Mardones,2021 | Chile | Basic health unit | - | Randomized controlled trial | Em torno de 1 ano | linical impact of medication reviews with follow-up in cardiovascular older adult patients | Pharmacotherapeutic follow-up; Health education | Método Dáder / Seguimento (Escola de Granada) | Método Dáder / Seguimento de Granada | Collaborative: shared case discussion and decision-making. | Clinical outcomes; Humanistic outcomes | 69 estudos / 11.644 pacientes | Patients with cardiovascular disease | Older adults | Não mencionado | Target population/context description; Adjustment/stratification/subgroup analysis | Age; Disability |
| 60 | Neto,2012; Neto,2013 | Brazil | Basic health unit | - | Prospective cohort study | 01/11/2010 - 31/11/2011 | Reações adversas a medicamentos causadas por interações medicamentosas | Medication review; Therapeutic drug monitoring | Método Dáder / Seguimento (Escola de Granada) | Método Dáder / Seguimento de Granada | Integrated/transdisciplinary: joint care or real-time shared health record. | Service/process or management indicators; Clinical outcomes | 433 pacientes / 12 UBS | Patients with polypharmacy | Older adults | > 60 anos | Not reported | Not reported |
| 61A | Mino-León, 2012 | Mexico | Basic health unit | - | Non-randomized clinical trial / quasi-experimental study | Não mencionado | Médicos e farmacêuticos: colaboração para melhorar a qualidade das prescrições na atenção primária no México | Medication review | Método Dáder / Seguimento (Escola de Granada) | Método Dáder / Seguimento de Granada | Integrated/transdisciplinary: joint care or real-time shared health record. | Service/process or management indicators | 4 farmacêuticos / 109 pacientes; 4 farmacêuticos / 160 pacientes / 69 médios | Patients with diabetes; Patients with hypertension | Adults | 48 - 68 anos | Not reported | Not reported |
| 62 | Correr,2011 | Brazil | Community pharmacy | - | Non-randomized clinical trial / quasi-experimental study | julho/2004 - março/2006 | Effects of a pharmacotherapy follow-up in community pharmacies on type 2 diabetes patients in Brazil | Pharmacotherapeutic follow-up | Método Dáder / Seguimento (Escola de Granada) | Método Dáder / Seguimento de Granada | Integrated/transdisciplinary: joint care or real-time shared health record. | Clinical outcomes | 6 farmácias / 96 pacientes | Patients with diabetes | Adults | > 30 anos | Not reported | Not reported |
| 65 | Souza, 2009 | Brazil | Other primary care setting | Farmácia Escola | Non-randomized clinical trial / quasi-experimental study | 09/2007 - 05/2008 | Método Dáder de Seguimento Farmacoterapêutico | Pharmacotherapeutic follow-up | Método Dáder / Seguimento (Escola de Granada) | Método Dáder / Seguimento de Granada | Referral-based: written referral or occasional phone contact. | Clinical outcomes | 10 pacientes | Patients with hypertension | Older adults | > 60 anos | Not reported | Not reported |
| 68 | Silva, 2024 | Brazil | Basic health unit | — | Mixed-methods study | novembro/2021 - abril/2022 | PHARMACEUTICAL CARE FOR PATIENTS WITH DIABETES MELLITUS | Other | Não citado explicitamente | Protocolo Próprio / Desenvolvido pelos Autores | Isolated: independent practice. | Humanistic outcomes | 14 UBS | Patients with diabetes | Not reported | - | Not reported | Not reported |
| 72 | Emiliano, 2013 | Brazil | Basic health unit | - | Case report / experience report | março/2011 | ASSISTÊNCIA FARMACÊUTICA E ATENÇÃO FARMACÊUTICA: | Pharmacotherapeutic follow-up | Método Dáder / Seguimento (Escola de Granada) | Método Dáder / Seguimento de Granada | Referral-based: written referral or occasional phone contact. | Service/process or management indicators | Não mencionado | Patients with hypertension; Patients with diabetes | Not reported | Não informado | Not reported | Not reported |
| 73 | Mino-León, 2014 | Mexico | Basic health unit | - | Non-randomized clinical trial / quasi-experimental study | junho/2005 - dezembro/2005 | ambulatory health care to improve drug treatment adherence and disease control | Health education | Não citado explicitamente | Não descrito / Não aplicável. | Referral-based: written referral or occasional phone contact. | Clinical outcomes; Humanistic outcomes | 440 pacientes | Patients with diabetes; Patients with hypertension | Mixed | Não informado | Not reported | Not reported |
| 75 | Igual ao 75 | Brazil | Basic health unit | - | Qualitative study | Não informado | IMPLANTAÇÃO DA ATENÇÃO FARMACÊUTICA EM UMA UNIDADE DE ATENÇÃO PRIMÁRIA À SAÚDE | Medication review | Assistência Farmacêutica (Política Brasileira) | Não descrito / Não aplicável. | Referral-based: written referral or occasional phone contact. | Service/process or management indicators | 5 trabalhados da saúde | General population | Not reported | Não informado | Not reported | Not reported |
| 80 | Paulós,2005; Paulós,2007 | Chile | Community pharmacy | - | Randomized controlled trial | 16 semanas | Impact of a Pharmaceutical Care Program | Pharmacotherapeutic follow-up; Health education | CMM - Gestão Integral da Farmacoterapia | CMM / GTM; Questionários Humanísticos (SF-36 | Referral-based: written referral or occasional phone contact. | Clinical outcomes; Service/process or management indicators; Humanistic outcomes | 42 pacientes | Patients with polypharmacy | Older adults | >50 anos | Eligibility criterion | Education; Disability |
| 83 | Rodrigues,2023 | Brazil | Basic health unit | - | Case report / experience report | 2014 - 2017 | Prescribing omissions and inclusion of new medications in the pharmacotherapy | Medication review | Atenção Farmacêutica (Hepler & Strand) | STOPP/START | Referral-based: written referral or occasional phone contact. | Service/process or management indicators | 585 pacientes | General population | Older adults | > 60 anos | Not reported | Not reported |
| 84 | Santos,2023 | Brazil | Basic health unit | - | Non-randomized clinical trial / quasi-experimental study | setembro/2021 - março/2022 | CUIDADO FARMACÊUTICO EM DIABETES MELLITUS TIPO 2 | Pharmacotherapeutic follow-up | Não citado explicitamente | Não descrito / Não aplicável. | Referral-based: written referral or occasional phone contact. | Clinical outcomes | 17 pacientes | Patients with diabetes | Mixed | 40 - 85 anos | Eligibility criterion | Education |
| 94 | Destro,2021 | Brazil | Basic health unit | - | Case report / experience report | maio/2016 | Desafios para o cuidado farmacêutico na Atenção Primária à Saúde | Other | Não citado explicitamente | Não descrito / Não aplicável. | Isolated: independent practice. | Service/process or management indicators | 50 farmacêuticos | General population | Adults | >30 anos | Not reported | Not reported |
| 99 | Santos,2020 | Brazil | Family Health Strategy service | - | Mixed-methods study | Não informado | Cuidado farmacêutico domiciliar | Pharmacotherapeutic follow-up | Não citado explicitamente | Protocolo Próprio / Desenvolvido pelos Autores | Referral-based: written referral or occasional phone contact. | Service/process or management indicators | 32 farmacêuticos | General population | Not reported | Não informado | Not reported | Not reported |
| 100 | Mendes,2020 | Brazil | Basic health unit | - | Qualitative study | outubro/2017 - novembro/2018 | Serviços Farmacêuticos na Atenção Primária à Saúde | Other | Não citado explicitamente | Não descrito / Não aplicável. | Isolated: independent practice. | Service/process or management indicators | 140 farmacêuticos / 3 unidades básicas de saúde | General population | Not reported | Não informado | Not reported | Not reported |
| 108 | Neves,2024 | Brazil | Community pharmacy | pública | Mixed-methods study | abril - outubro/2019 | Drug dispensing in public community pharmacies | Clinically oriented dispensing | Não citado explicitamente | Não descrito / Não aplicável. | Isolated: independent practice. | Service/process or management indicators | 24 farmacêuticos | General population | Adults | >30 anos | — | — |
| 113 | Esandi,2024 | Argentina | Community pharmacy | privada | Mixed-methods study | outubro/2020 - março/2022 | Evaluating the implementation of a hypertension program based on mHealth and community pharmacies integration to primary care centers at a municipality level in Argentina during the COVID-19 pandemic | Health education | Não citado explicitamente | Protocolo Próprio / Desenvolvido pelos Autores | Referral-based: written referral or occasional phone contact. | Clinical outcomes; Humanistic outcomes | 440 participantes | Patients with hypertension | Adults | > 18 anos | Eligibility criterion | Education; Age |
| 114 | Lira-Meriguete,2023 | Brazil | Basic health unit | - | Prospective cohort study | junho/2016 - janeiro/2018 | Pharmaceutical care improves medication adherence and quality of life in type 2 diabetes mellitus | Pharmacotherapeutic follow-up | Não citado explicitamente | Protocolo Clínico Institucional / Ministério da Saúde | Referral-based: written referral or occasional phone contact. | Clinical outcomes; Humanistic outcomes | 75 pacientes | Patients with diabetes | Adults | 40 - 70 anos; 41 - 70 anos | Not reported | Not reported |
| 115 | Rezende, 2023 | Brazil | Basic health unit | - | Non-randomized clinical trial / quasi-experimental study | fevereiro/2015 - fevereiro/2017 | Clinical impact of comprehensive medication management services | Pharmacotherapeutic follow-up | CMM - Gestão Integral da Farmacoterapia; Método Dáder / Seguimento (Escola de Granada) | Método Dáder / Seguimento de Granada | Referral-based: written referral or occasional phone contact. | Service/process or management indicators; Clinical outcomes | 346 pacientes | Patients with hypertension; Patients with polypharmacy | Older adults | > 60 anos | Not reported | Not reported |
| 119 | Lara-Júnior, 2022 | Brazil | Basic health unit | - | Mixed-methods study | janeiro/2018 - fevereiro/2020 | Implementation and Effectiveness of a Pharmacotherapeutic Follow-Up Service | Pharmacotherapeutic follow-up | Assistência Farmacêutica (Política Brasileira) | Protocolo Clínico Institucional / Ministério da Saúde | Referral-based: written referral or occasional phone contact. | Service/process or management indicators | 721 pacientes / 148 UBS / 82 farmacêuticos | Patients with polypharmacy | Adults | > 40 anos | Not reported | Not reported |
| 120 | Melo, 2017 | Brazil | Basic health unit | - | Cross-sectional study | 2010 - 2011 | A contribuição do farmacêutico para a promoção do acesso e uso racional de medicamentos essenciais no SUS | Pharmacotherapeutic follow-up; Health education | Não citado explicitamente | Não descrito / Não aplicável. | Collaborative: shared case discussion and decision-making. | Service/process or management indicators | Não explicito | General population | Not reported | Não informado | Not reported | Not reported |
| 127 | Oliveira, 2016 | Brazil | Community pharmacy | comunitária | Cross-sectional study | janeiro/2012 - dezembro/2015 | The good pharmacy practice | Medication review | Não citado explicitamente | Não descrito / Não aplicável. | Referral-based: written referral or occasional phone contact. | Service/process or management indicators | Não explicito | General population | Not reported | Não informado | Not reported | Not reported |
| 129 | Nakamura,2016 | Brazil | Family Health Strategy service | - | Qualitative study | março/2012 - dezembro/2012 | A construção do processo de trabalho no Núcleo de Apoio à Saúde da Família: a experiência dos farmacêuticos | Other | Não citado explicitamente | Não descrito / Não aplicável. | Referral-based: written referral or occasional phone contact. | Service/process or management indicators | 7 farmacêuticos | General population | Adults | Não informado | Not reported | Not reported |
| 132 | Mauricio,2016 | Brazil | Basic health unit | - | Cross-sectional study | julho - outubro/2015 | Potentially inappropriate medication use among Brazilian elderly | Medication review | Farmácia Clínica (ACCP - Americano)o 1; Não citado explicitamente; Atenção Farmacêutica (Hepler & Strand) | Critérios de Rastreio (Beers; CMM / GTM; PWDT / Hepler & Strand (Pharmacotherapy Workup)Opção 2 | Referral-based: written referral or occasional phone contact. | Service/process or management indicators | 15 pacientes | General population | Older adults | > 60 anos | Not reported | Not reported |
| 133 | Laborne-e-Valle,2022 | Brazil | Basic health unit | - | Mixed-methods study | janeiro/2018 - dezembro/2019 | Assessment of Pharmaceutical Services for Smoking Cessation | Pharmacotherapeutic follow-up | Uso Racional de Medicamentos (OMS) | Protocolo Clínico Institucional / Ministério da Saúde | Integrated/transdisciplinary: joint care or real-time shared health record. | Service/process or management indicators | 170 abordagens individuas / 1591 abordagens em grupo | Smokers | Not reported | Não informado | Not reported | Not reported |
| 136 | Mendes,2022 | Brazil | Basic health unit | - | Mixed-methods study | novembro/2016 - junho/2017 | A broad view of pharmaceutical services in multidisciplinary teams | Other | Não citado explicitamente | Não descrito / Não aplicável. | Referral-based: written referral or occasional phone contact. | Service/process or management indicators | 200 farmacêuticos | General population | Adults | média 35 anos | Not reported | Not reported |
| 138 | Santos,2022 | Brazil | Basic health unit | - | Experience report | março - agosto/2022 | Intervenção farmacêutica para orientação de pacientes asmáticos | Health education | Não citado explicitamente | Não descrito / Não aplicável. | Referral-based: written referral or occasional phone contact. | Service/process or management indicators | Não explicíto | Patients with asthma | Mixed | Não informado | Not reported | Not reported |
| 139 | Silva, 2022 | Brazil | Community pharmacy | - | Cross-sectional study | julho/2016 - outubro/2018 | Profile of pharmaceutical interventions of a pharmacotherapeutic follow-up model | Pharmacotherapeutic follow-up | Método Dáder / Seguimento (Escola de Granada) | Método Dáder / Seguimento de Granada; SOAP / TOM | Referral-based: written referral or occasional phone contact. | Service/process or management indicators | 17 pessoas | Patients with diabetes | Mixed | > 18 anos | Eligibility criterion | Education |
| 140 | Barros,2022 | Brazil | Basic health unit | - | Cross-sectional study | dezembro/2016 - março/2017 | Clinical pharmaceutical services in primary health | Other | Não citado explicitamente | Protocolo Clínico Institucional / Ministério da Saúde | Referral-based: written referral or occasional phone contact. | Service/process or management indicators | 34 farmacêuticos | General population | Adults | média 39 anos | Not reported | Not reported |
| 143 | Coqueiros,2022 | Brazil | Basic health unit | - | Qualitative study | Não informado | Desafios e possibilidades para a inserção de farmacêuticos na Estratégia Saúde da Família | Other | Não citado explicitamente | Não descrito / Não aplicável. | Referral-based: written referral or occasional phone contact. | Service/process or management indicators | 15 participantes | General population | Not reported | Não informando | Not reported | Not reported |
| 150 | Gome,2022 | Brazil | Basic health unit | - | Cross-sectional study | 2015 - 2017 | Pharmaceutical Care in Primary Care | Pharmacotherapeutic follow-up | Método Dáder / Seguimento (Escola de Granada) | Escalas de Adesão (Morisky; Método Dáder / Seguimento de Granada | Referral-based: written referral or occasional phone contact. | Humanistic outcomes; Clinical outcomes | 163 pacientes | Patients with hypertension | Adults | > 18 anos | Target population/context description | Education; Socioeconomic status |
| 151 | Martins, 2022 | Brazil | Basic health unit | - | Mixed-methods study | abril/2015 - fevereiro/2016 | Potentially inappropriate medicaition use in a comprehensive therapy management service | Pharmacotherapeutic follow-up | CMM - Gestão Integral da Farmacoterapia; Atenção Farmacêutica (Hepler & Strand) | Critérios de Rastreio (Beers; PWDT / Hepler & Strand (Pharmacotherapy Workup)Opção 2 | Collaborative: shared case discussion and decision-making. | Clinical outcomes; Service/process or management indicators | 389 paciente | General population | Older adults | > 60 anos | Not reported | Not reported |
| 152 | Dias,2022 | Brazil | Basic health unit | - | Cross-sectional study | fevereiro/2015 - fevereiro/2017 | Factors associated with the identification of drug therapy problems | Pharmacotherapeutic follow-up | CMM - Gestão Integral da Farmacoterapia | PWDT / Hepler & Strand (Pharmacotherapy Workup)Opção 2 | Referral-based: written referral or occasional phone contact. | Service/process or management indicators | 758 pacientes | General population | Older adults | > 60 anos | Not reported | Not reported |
| 154 | Silva,2015 | Brazil | Basic health unit | - | Experience report | 2014-2015 | A IMPORTÂNCIA DA FARMÁCIA CLÍNICA NO ACOMPANHAMENTO DOS PACIENTES | Pharmacotherapeutic follow-up | Método Dáder / Seguimento (Escola de Granada) | Método Dáder / Seguimento de Granada | Referral-based: written referral or occasional phone contact. | Service/process or management indicators | 25 pacientes | Patients with polypharmacy | Adults | 40 - 59 anos | Not reported | Not reported |
| 155 | silva,2021 | Brazil | Basic health unit | - | Mixed-methods study | dezembro/2017 - junho/2018 | Profile of pharmaceutical care in primary health | Other | Não citado explicitamente | Não descrito / Não aplicável. | Collaborative: shared case discussion and decision-making. | Service/process or management indicators | 24 farmacêuticos | General population | Adults | 30 - 40 anos | Not reported | Not reported |
| 162 | Sartor, 2014 | Brazil | Basic health unit | - | Methodological study (instrument validation); Qualitative study | junho/2009 - março/2014 | Model for the evaluation of drug-dispensing services | Clinically oriented dispensing; Medication review | Não citado explicitamente | Protocolo Próprio / Desenvolvido pelos Autores | Referral-based: written referral or occasional phone contact. | Service/process or management indicators | 15 UBS | General population | Not reported | Não informado | Not reported | Not reported |
| 163 | Silva,2021 | Brazil | Basic health unit | - | Cross-sectional study | setembro/2018 - fevereiro/2019 | Cuidado Farmacêutico: Revisão da Farmacoterapia de Usuários | Medication review | Não citado explicitamente | Não descrito / Não aplicável. | Referral-based: written referral or occasional phone contact. | Service/process or management indicators | 91 pacientes | General population | Mixed | > 20 anos | Eligibility criterion | Education |
| 166 | Santos-Pinto, 2021 | Brazil | Basic health unit | - | Cross-sectional study; Qualitative study | novembro - dezembro/2017 | The role of primary care pharmacists in the response to the Zika epidemic | Other | — | Protocolo Próprio / Desenvolvido pelos Autores | Referral-based: written referral or occasional phone contact. | Service/process or management indicators | 42 farmacêuticos | General population | Not reported | Não informado | Not reported | Not reported |
| 173 | Packeiser,2020 | Brazil | Other primary care setting | Universidade Federal do Rio Grande do Sul (UFRGS) | Cross-sectional study | 2012 - 2015 | Evaluation of simulated drug dispensing and patient counseling in the course of pharmaceutical improvement: | Clinically oriented dispensing | Não citado explicitamente | Simulação / Role-play | Isolated: independent practice. | Service/process or management indicators | 459 estagiários | General population | Not reported | Não informado | Not reported | Not reported |
| 175 | Ceballos,2020; Ceballos,2021 | Colombia | Community pharmacy | privada | Prospective cohort study; Randomized controlled trial | dezembro/2018 - dezembro/2019 | Evaluation of the effects of a drug with fiscalized substance dispensation, health education, and pharmacovigilance continuing education program | Health education; Clinically oriented dispensing | Uso Racional de Medicamentos (OMS) | Protocolo Próprio / Desenvolvido pelos Autores; Simulação / Role-play | Isolated: independent practice. | Service/process or management indicators | 135 drogaria/farmácia / 3 pacientes/cuidadores de cada farmácia | General population | Adults | > 18 anos | Not reported | Not reported |
| 178 | Silva,2020 | Brazil | Ambulatory care | - | Case report / experience report | 30/08/2016 - junho/2017 | Implementation of pharmaceutical care | Pharmacotherapeutic follow-up | Método Dáder / Seguimento (Escola de Granada) | Método Dáder / Seguimento de Granada | Collaborative: shared case discussion and decision-making. | Service/process or management indicators | 11 profissionais da saúde / 3 usuários | General population | Older adults | > 60 anos | Not reported | Not reported |
| 179 | Reis, 2013 | Brazil | Ambulatory care | - | Non-randomized clinical trial / quasi-experimental study | março/2009 - janeiro/2011 | Consejos farmacéuticos a pacientes con trastornos de humor | Health education | Não citado explicitamente | Escalas de Adesão (Morisky | Isolated: independent practice. | Humanistic outcomes; Clinical outcomes | 19 pacientes | Patients with mental disorders | Mixed | 15 - 82 anos | Eligibility criterion | Disability |
| 187 | Santos,2018 | Brazil | Basic health unit | — | Retrospective cohort study | maio - novembro/2016 | Pharmaceutical clinical services in basic care | Pharmacotherapeutic follow-up | Assistência Farmacêutica (Política Brasileira) | Não descrito / Não aplicável. | Referral-based: written referral or occasional phone contact. | Service/process or management indicators | 1080 usuário / 12 UBS | General population | Mixed | não mencionado | Not reported | Not reported |
| 190 | Silva,2017 | Brazil | Other primary care setting | Universidade | Qualitative study | 2014 - 2016 | Autoethnography as an Instrument for Professional (Trans) Formation in Pharmaceutical Care Practice | Other | Assistência Farmacêutica (Política Brasileira) | Não descrito / Não aplicável. | Collaborative: shared case discussion and decision-making. | Service/process or management indicators | 5 farmacêuticos | General population | Not reported | não mencionado | Not reported | Not reported |
| 192 | Molino,2017 | Brazil | Ambulatory care | Hospital Universitário | Randomized controlled trial | janeiro/2009 - junho/2012 | HIV pharmaceutical care in primary healthcare: | Pharmacotherapeutic follow-up | Não citado explicitamente | Não descrito / Não aplicável. | Referral-based: written referral or occasional phone contact. | Service/process or management indicators; Clinical outcomes | 143 pacientes | Patients with polypharmacy | Mixed | 18 - 65 | Eligibility criterion | Socioeconomic status |
| 198 | Araujo,2017 | Brazil | Basic health unit | - | Cross-sectional study | não informado | Atividades farmacêuticas de natureza clínica | Pharmacotherapeutic follow-up | Assistência Farmacêutica (Política Brasileira) | Não descrito / Não aplicável. | Collaborative: shared case discussion and decision-making. | Service/process or management indicators | 285 farmacêuticos | General population | Adults | 30 - 59 anos | Not reported | Not reported |
| 201 | Lima,2017 | Brazil | Basic health unit | - | Cross-sectional study | julho/2014 - maio/2015 | Indicadores relacionados ao uso racional de medicamentos | Clinically oriented dispensing | Uso Racional de Medicamentos (OMS) | Não descrito / Não aplicável. | Referral-based: written referral or occasional phone contact. | Service/process or management indicators | 8803 usuários | General population | Not reported | Não informado | Not reported | Not reported |
| 203 | Plaster,2012 | Brazil | Basic health unit | - | Randomized controlled trial | outubro/2008 - abril/2009 | a pharmaceutical care program of pharmacotherapy follow-up | Pharmacotherapeutic follow-up | Método Dáder / Seguimento (Escola de Granada) | Escalas de Adesão (Morisky; Método Dáder / Seguimento de Granada | Integrated/transdisciplinary: joint care or real-time shared health record. | Humanistic outcomes; Clinical outcomes | 120 pacientes | Patients with diabetes | Mixed | > 30 anos | Not reported | Not reported |
| 209 | Provin,2010 | Brazil | Family Health Strategy service | — | Case report / experience report | 12 meses | Atenção Farmacêutica em Goiânia: | Pharmacotherapeutic follow-up | Não citado explicitamente | Protocolo Próprio / Desenvolvido pelos Autores | Referral-based: written referral or occasional phone contact. | Service/process or management indicators | 50 pacientes | General population | Adults | média 50 anos | Not reported | Not reported |
| 211 | Luz,2017 | Brazil | Basic health unit | - | Mixed-methods study | fevereiro - agosto/2014 | Pharmaceutical Services in Primary Health Care: are pharmacists and users on the same page? | Other | Assistência Farmacêutica (Política Brasileira) | Não descrito / Não aplicável. | Referral-based: written referral or occasional phone contact. | Service/process or management indicators | 69 usuários / 4 farmacêuticos | General population | Adults | > 18 anos | Not reported | Not reported |
| 214 | Mendonça,2016 | Brazil | Ambulatory care | - | Retrospective cohort study | outubro/2010 - fevereiro/2013 | Clinical outcomes of medication therapy management services in primary health care | Pharmacotherapeutic follow-up | Atenção Farmacêutica (Hepler & Strand) | CMM / GTM | Isolated: independent practice. | Clinical outcomes; Service/process or management indicators | 92 pacientes | Patients with polypharmacy | Older adults | > 60 anos | Not reported | Not reported |
| 216 | Cazarim,2016 | Brazil | Basic health unit | - | Non-randomized clinical trial / quasi-experimental study | 2006 - 2012; 2007 - 2012; 2008 - 2012 | Impact Assessment of Pharmaceutical Care in the Management of Hypertension and Coronary Risk Factors after Discharge | Pharmacotherapeutic follow-up; Health education | Não citado explicitamente | PWDT / Hepler & Strand (Pharmacotherapy Workup)Opção 2; Questionários Humanísticos (SF-36; Escalas de Adesão (Morisky | Referral-based: written referral or occasional phone contact. | Clinical outcomes; Humanistic outcomes | 104 pacientes | Patients with hypertension | Adults | > 20 anos | Not reported | Not reported |
| 224 | Firmino, 2015 | Brazil | Basic health unit | — | Randomized controlled trial | outubro/2011 - setembro/2012 | Cardiovascular risk rate in hypertensive patients attended in primary health care units: the influence of pharmaceutical care | Pharmacotherapeutic follow-up | Método Dáder / Seguimento (Escola de Granada) | Método Dáder / Seguimento de Granada | Referral-based: written referral or occasional phone contact. | Clinical outcomes; Service/process or management indicators | 68 pacientes | Patients with hypertension | Not reported | Não informado | Not reported | Not reported |
| 225 | Cani,2015 | Brazil | Other primary care setting | Hospital universitário | Randomized controlled trial | 6 meses | Improvement in medication adherence and selfmanagement of diabetes with a clinical pharmacy program: | Medication review; Health education | Não citado explicitamente | Escalas de Adesão (Morisky | Referral-based: written referral or occasional phone contact. | Clinical outcomes; Humanistic outcomes | 70 adultos | Patients with diabetes | Mixed | >45 anos | Not reported | Not reported |
| 228 | Nakamura,2015 | Brazil | Family Health Strategy service | - | Qualitative study | março-dezembro/2015 | Pharmaceutical Services in Family Health Support Team | Other | Não citado explicitamente | Não descrito / Não aplicável. | Referral-based: written referral or occasional phone contact. | Service/process or management indicators | Não mencionado | General population | Not reported | Não informado | Not reported | Not reported |
| 231 | Salazar-Ospina,2014 | Colombia | Ambulatory care | - | Randomized controlled trial | novembro/2011 - junho/2013 | Effectiveness of the Dader Method for pharmaceutical care in patients with bipolar I disorder | Pharmacotherapeutic follow-up | Método Dáder / Seguimento (Escola de Granada) | Escalas de Adesão (Morisky; Método Dáder / Seguimento de Granada; Questionários Humanísticos (SF-36 | Referral-based: written referral or occasional phone contact. | Humanistic outcomes; Service/process or management indicators; Clinical outcomes | 200 pacientes | Patients with mental disorders | Adults | 18 - 65 anos | Eligibility criterion | Education; Disability |
| 241 | Martins,2013 | Brazil | Basic health unit | - | Retrospective cohort study | setembro/2010 - fevereiro/2012 | Pharmaceutical Care for hypertensive patients | Pharmacotherapeutic follow-up | Não citado explicitamente | PWDT / Hepler & Strand (Pharmacotherapy Workup)Opção 2; Escalas de Adesão (Morisky | Referral-based: written referral or occasional phone contact. | Service/process or management indicators; Clinical outcomes | 14 pacientes | Patients with hypertension | Adults | 30 - 74 anos | Not reported | Not reported |
| 242 | Mourão,2013 | Brazil | Basic health unit | - | Randomized controlled trial | abril - setembro/2010 | Pharmaceutical care program | Pharmacotherapeutic follow-up | Não citado explicitamente | Não descrito / Não aplicável. | Referral-based: written referral or occasional phone contact. | Clinical outcomes | 100 pacientes | Patients with diabetes | Mixed | >18 anos | Not reported | Not reported |
| 243 | Badesso,2013 | Argentina | Community pharmacy | - | Randomized controlled trial | julho - dezembro/2011 | Effect of pharmacotherapy follow-up | Pharmacotherapeutic follow-up | Método Dáder / Seguimento (Escola de Granada) | Método Dáder / Seguimento de Granada | Referral-based: written referral or occasional phone contact. | Service/process or management indicators; Clinical outcomes | 101 pacientes | Patients with diabetes | Mixed | 18 - 75 anos | Not reported | Not reported |
| 245 | Obreli-Neto,2011 | Brazil | Basic health unit | - | Randomized controlled trial | outubro/2006 - outubro/2009 | Effect of a 36-month pharmaceutical care program on pharmacotherapy adherence | Pharmacotherapeutic follow-up | Atenção Farmacêutica (Hepler & Strand) | Escalas de Adesão (Morisky; PWDT / Hepler & Strand (Pharmacotherapy Workup)Opção 2 | Referral-based: written referral or occasional phone contact. | Humanistic outcomes; Clinical outcomes | 200 pacientes | Patients with hypertension; Patients with diabetes | Older adults | > 60 anos | Not reported | Not reported |
| 249 | Gualda,2009; Marcelo et al., 2009 | Argentina | Other primary care setting; Basic health unit | Trabalho de campo | Retrospective cohort study; Case series | março/2007 - dezembro/2008; março de 2007 dezembro de 2008 | Utilización de Medicamentos en un Programa de Atención Primaria de Salud para Poblaciones Rurales Marginales \| Educação sanitária \| Dispensação | Health education; Clinically oriented dispensing | Uso Racional de Medicamentos (OMS); Não citado explicitamente | Protocolo Clínico Institucional / Ministério da Saúde; Protocolo Próprio / Desenvolvido pelos Autores | Collaborative: shared case discussion and decision-making.; Integrated/transdisciplinary: joint care or real-time shared health record. | Service/process or management indicators; Humanistic outcomes; Not reported | 608 prescrições; 608 | General population | Not reported | Não informado | Not reported; Target population/context description | Not reported; Place of residence |
| 252 | Júnior,2008 | Brazil | Ambulatory care | - | Prospective cohort study | agosto/2003 - julho/2004 | Effect of pharmaceutical care intervention | Pharmacotherapeutic follow-up; Health education | Não citado explicitamente | Não descrito / Não aplicável. | Collaborative: shared case discussion and decision-making. | Service/process or management indicators; Humanistic outcomes; Clinical outcomes | 30 pacientes | Patients with hypertension | Older adults | >60 anos | Not reported | Not reported |
| 253 | Vieira,2008 | Brazil | Basic health unit; Family Health Strategy service | - | Mixed-methods study | março - maio/2006 | Avaliação da assistência farmacêutica | Clinically oriented dispensing; Medication reconciliation | Uso Racional de Medicamentos (OMS) | Protocolo Clínico Institucional / Ministério da Saúde | Isolated: independent practice. | Service/process or management indicators | Não informado | Pregnant women | Not reported | Não informado | Not reported | Not reported |
| 254 | Júnior,2007 | Brazil | Basic health unit | - | Prospective cohort study | agosto/2003 - julho/2004 | Impact of Pharmaceutical Care interventions in the identifi cation and resolution of drug-related problems | Health education; Pharmacotherapeutic follow-up | Não citado explicitamente | Questionários Humanísticos (SF-36; EuroQol | Referral-based: written referral or occasional phone contact. | Service/process or management indicators; Humanistic outcomes | 30 pacientes | Patients with hypertension | Older adults | 60 - 75 anos | Not reported | Not reported |
| 260 | Júnior,2007 | Brazil | Basic health unit | - | Prospective cohort study | julho/2003 - julho/2004 | Influence of Pharmaceutical Care intervention and communication skills on the improvement o pharmacotherapeutic outcomes | Pharmacotherapeutic follow-up; Health education | Não citado explicitamente | Não citado explicitamente | Referral-based: written referral or occasional phone contact. | Service/process or management indicators; Humanistic outcomes | 30 pacientes | Patients with hypertension | Older adults | média 68 anos | Not reported | Not reported |
| 263 | Hass,2006 | Brazil | Community pharmacy | - | Prospective cohort study | maio - julho/2003 | ACOMPANHAMENTO FARMACOTERAPÊUTICO DE CRIANÇAS SOB PRESCRIÇÃO DE ANTIMICROBIANOS. | Pharmacotherapeutic follow-up | Não citado explicitamente | Não citado explicitamente | Referral-based: written referral or occasional phone contact. | Service/process or management indicators; Humanistic outcomes | 85 crianças | Patients with polypharmacy | Children | 0 - 15 anos | Not reported | Not reported |
| 265 | Oliveira;2004 | Brazil | Community pharmacy | - | Qualitative study | Não informado | Atenção farmacêutica | Clinically oriented dispensing | Não citado explicitamente | Não descrito / Não aplicável. | Isolated: independent practice. | Service/process or management indicators | Não mencionado | Patients with diabetes | Not reported | Não informado | Not reported | Not reported |
| 267 | Ferraes,2002 | Brazil | Other primary care setting | - | Mixed-methods study | Não informado | POLÍTICA DE MEDICAMENTOS NA ATENÇÃO BÁSICA E A ASSISTÊNCIA FARMACÊUTICA | Other | Atenção Farmacêutica (Hepler & Strand); Assistência Farmacêutica (Política Brasileira) | Protocolo Próprio / Desenvolvido pelos Autores | Isolated: independent practice. | Economic outcomes; Service/process or management indicators | 108 gestores | General population | Not reported | Não informado | Not reported | Not reported |
| 271 | Agudelo, 2003 | Colombia | Basic health unit | - | Before-and-after study (without control group) | março/2000 - fevereiro/2001 | Impact of pharmacist intervention in the ambulatory care process | Pharmacotherapeutic follow-up | Método Dáder / Seguimento (Escola de Granada) | Método Dáder / Seguimento de Granada | Integrated/transdisciplinary: joint care or real-time shared health record. | Service/process or management indicators; Humanistic outcomes; Economic outcomes | 625 pacientes | General population | Not reported | Não informado | Not reported | Not reported |
| 278 | Cerqueira-Santos,2022 | Brazil | Community pharmacy | - | Cross-sectional study | maio - julho/2021 | Perception of community pharmacists about the work process of drug dispensing: | Clinically oriented dispensing | Não citado explicitamente | Protocolo Próprio / Desenvolvido pelos Autores | Isolated: independent practice. | Service/process or management indicators | 625 farmacêuticos | General population | Not reported | Não informado | Not reported | Not reported |
| 284 | Ahumada-Canale,2021; Ahumada-Canalea et al., 2021 | Chile | Basic health unit | - | Randomized controlled trial | janeiro/2018 - julho/2019; Janeiro de 2018 a julho de 2019 | Cost-utility analysis of medication review with follow-up for cardiovascular outcomes: \| Revisão farmacoterapeutica \| Acompanhamento farmacoterapeutico | Pharmacotherapeutic follow-up; Medication review | Não citado explicitamente | Simulação / Role-play; Protocolo Próprio / Desenvolvido pelos Autores | Referral-based: written referral or occasional phone contact.; Isolated: independent practice. | Economic outcomes | 283 pacientes; 283 | Patients with polypharmacy; Patients with cardiovascular disease | Older adults | >65 anos; > 65 anos | Not reported; Eligibility criterion | Not reported; Age |
| OATD44 | Saldivia, 2020 | Chile | Basic health unit | - | Qualitative study | abril - julho/2019 outubro/2019 - janeiro/2020 | EXPERIENCIA FARMACOTERAPÉUTICA COMO INSTRUMENTO DE DETECCIÓN DE FACTORES DE NO ADHERENCIA TERAPÉUTICA, | Health education | Não citado explicitamente | Protocolo Próprio / Desenvolvido pelos Autores | Isolated: independent practice. | Humanistic outcomes | 10 pacientes | Patients with diabetes | Adults | 47 - 69 anos | Equity discussion/social barriers | Education |
| OATD36 | Firmino, 2013 | Brazil | Basic health unit | - | Randomized controlled trial | outubro/2021 - setemrbo/2012 | A INFLUÊNCIA DA ATENÇÃO FARMACÊUTICA NA TAXA DE RISCO CARDIO- VASCULAR | Pharmacotherapeutic follow-up | Método Dáder / Seguimento (Escola de Granada) | Método Dáder / Seguimento de Granada | Collaborative: shared case discussion and decision-making. | Clinical outcomes; Service/process or management indicators | 68 pacientes | Patients with hypertension | Adults | >18 anos | Eligibility criterion | Disability |
| OATD21 | Santos, 2018 | Brazil | Basic health unit | - | Non-randomized clinical trial / quasi-experimental study | julho/2014 - novembro/2016 | IMPACTO CLÍNICO DE UM SERVIÇO DE GERENCIAMENTO DA TERAPIA MEDICAMENTOSA | Pharmacotherapeutic follow-up | Atenção Farmacêutica (Hepler & Strand) | PWDT / Hepler & Strand (Pharmacotherapy Workup)Opção 2 | Collaborative: shared case discussion and decision-making. | Service/process or management indicators; Clinical outcomes | 1057 pacientes | General population | Mixed | 18 - 98 anos | Not reported | Not reported |
| OATD01 | Oliveira, 2018 | Brazil | Mixed setting | - | Qualitative study | abril/2013 - agosto/2015 | construção de um serviço de gerenciamento de terapia medicamentosa | Medication review | Não citado explicitamente | Não descrito / Não aplicável. | Collaborative: shared case discussion and decision-making. | Service/process or management indicators | 10 farmacêuticos, 1 enfermeiro e médico | General population | Not reported | - | Not reported | Not reported |
| BDTD156 | Zarpelon, 2023 | Brazil | Other primary care setting | Farmácia Escola | Methodological study (instrument validation); Randomized controlled trial | Não informado | TELECONSULTA FARMACÊUTICA PARA PACIENTES ADULTOS COM ASMA: PROTOCOLO PARA ADAPTAÇÃO, ESTRUTURAÇÃO E IMPLANTAÇÃO EMFARMÁCIA ESCOLA | Therapeutic drug monitoring | Não citado explicitamente | Protocolo Próprio / Desenvolvido pelos Autores | Referral-based: written referral or occasional phone contact. | Service/process or management indicators | Não mencionado | General population | Not reported | - | Not reported | Not reported |
| CAPES08 | Majewski, 2019 | Brazil | Community pharmacy | - | Qualitative study | fevereiro - maio/2016 | Responsabilidade social do farmacêutico à luz das experiências de pacientes: | Pharmacotherapeutic follow-up | Não citado explicitamente | Protocolo Próprio / Desenvolvido pelos Autores | Referral-based: written referral or occasional phone contact. | Humanistic outcomes | 15 usuários | General population | Mixed | 25 - 64 anos | Not reported | Not reported |
| BDTD95 | Sampaio, 2024 | Brazil | Basic health unit | - | Cross-sectional study | agosto/2022 - julho/2023 | ACOMPANHAMENTO FARMACOTERAPÊUTICO DE GESTANTES EM TRATAMENTO PARA TOXOPLASMOSE: | Pharmacotherapeutic follow-up | Método Dáder / Seguimento (Escola de Granada) | Método Dáder / Seguimento de Granada | Referral-based: written referral or occasional phone contact. | Service/process or management indicators | 41 pacientes | Pregnant women | Adults | não informado | Not reported | Not reported |
| BDTD84 | Moreira,2023 | Brazil | Other primary care setting | Domiciliar | Randomized controlled trial | junho/2021 - agosto/2022 | USO DO MONITORAMENTO DOMICILIAR DA PRESSÃO ARTERIAL PARA OTIMIZAÇÃO DO TRATAMENTO DA HIPERTENSÃO | Medication review | Não citado explicitamente | Não descrito / Não aplicável. | Referral-based: written referral or occasional phone contact. | Service/process or management indicators; Clinical outcomes | 161 individuos | Patients with hypertension | Older adults | > 60 anos | Not reported | Not reported |
| BDTD47 | Neto,2011 | Brazil | Basic health unit | - | Prospective cohort study | Não mencionado | INTERVENÇÕES FARMACÊUTICAS PARA PROMOÇÃO DO USO RACIONAL DE MEDICAMENTOS EM DOENÇAS CRÔNICAS NÃO-TRANSMISSÍVEIS, | Pharmacotherapeutic follow-up | Método Dáder / Seguimento (Escola de Granada) | Critérios de Rastreio (Beers; Método Dáder / Seguimento de Granada | Referral-based: written referral or occasional phone contact. | Humanistic outcomes; Service/process or management indicators | 106 pacientes | Patients with hypertension; Patients with diabetes | Older adults | > 60 anos | Not reported | Not reported |
| BDTD38 | Pereira,2019 | Brazil | Basic health unit | - | Mixed-methods study; Case report / experience report | Não mencionado | Análise da Implementação dos Serviços Farmacêuticos | Other | Não citado explicitamente | Não descrito / Não aplicável. | Isolated: independent practice. | Service/process or management indicators | 456 municípios | General population | Not reported | Não informado | Not reported | Not reported |
| BDTD31 | Carvalho, 2022 | Brazil | Basic health unit | - | Cross-sectional study | 20/10/2021 - 13/11/20021 | CULTURA ORGANIZACIONAL PARA SEGURANÇA DO PACIENTE EM FARMÁCIAS | Other | Não citado explicitamente | Protocolo Próprio / Desenvolvido pelos Autores | Isolated: independent practice. | Service/process or management indicators | 94 profissionais de saúde 10 farmacêuticos | General population | Not reported | Não informado | Not reported | Not reported |
| BDTD25 | Caetano, 2019 | Brazil | Family Health Strategy service | - | Case report / experience report | Não mencionado | Convergências e dilemas dos Serviços Farmacêuticos | Other | Não citado explicitamente | Protocolo Próprio / Desenvolvido pelos Autores | Isolated: independent practice. | Service/process or management indicators | 14 entrevistados | General population | Adults | 30 - 49 anos | Not reported | Not reported |
| BDTD02 | Silveira,2023 | Brazil | Basic health unit | - | Cross-sectional study | junho - dezembro/2021 | ANÁLISE DOS SERVIÇOS DE ASSISTÊNCIA FARMACÊUTICA | Other | Não citado explicitamente | Protocolo Próprio / Desenvolvido pelos Autores | Isolated: independent practice. | Service/process or management indicators | 52 profissionais | General population | Not reported | Não informado | Not reported | Not reported |
| OATD03 | BARROS, 2018 | Brazil | Basic health unit | — | Mixed-methods study | de outubro de 2016 a março de 2017 | serviços farmacêuticos clínicos da atenção primária | Other | Não citado explicitamente | Não descrito / Não aplicável. | Collaborative: shared case discussion and decision-making. | Service/process or management indicators | 172 unidades de saúde | General population | Not reported | não informado | Not reported | Not reported |
| OATD22 | Silva, 2024; Silva, 2025; Silva, 2026 | Brazil | Community pharmacy | — | Randomized controlled trial | de junho de 2021 a agosto de 2022 | visa avaliar a desprescrição de medicamentos anti-hipertensivos, orientada por farmacêuticos | Pharmacotherapeutic follow-up; Therapeutic drug monitoring; Medication review; Clinically oriented dispensing | Não citado explicitamente | Não descrito / Não aplicável. | Collaborative: shared case discussion and decision-making. | Clinical outcomes; Humanistic outcomes; Service/process or management indicators | 72 pacientes | Patients with hypertension | Older adults | acima de 60 anos | Not reported | Not reported |
| OATD43 | Trindade, 2015; Trindade, 2016; Trindade, 2017 | Brazil | Family Health Strategy service | — | Prospective cohort study | de novembro de 2013 a junho de 2014 | ATENÇÃO FARMACÊUTICA | Health education; Therapeutic drug monitoring; Pharmacotherapeutic follow-up | Atenção Farmacêutica (Hepler & Strand) | PWDT / Hepler & Strand (Pharmacotherapy Workup)Opção 2; Escalas de Adesão (Morisky | Referral-based: written referral or occasional phone contact. | Clinical outcomes; Humanistic outcomes; Service/process or management indicators | 55 pacientes; 56 pacientes; 57 pacientes | Patients with diabetes; Patients with hypertension | Older adults | acima de 60 anos | Intervention/implementation adaptation; Equity discussion/social barriers | Education; Socioeconomic status |
| CAPES05 | Solbiati, 2024 | Brazil | Community pharmacy | — | Mixed-methods study | de novembro de 2022 a abril de 2023 | PAPEL DO FARMACÊUTICO COMO EDUCADOR EM SAÚDE | Health education | Não citado explicitamente | Não descrito / Não aplicável. | Isolated: independent practice. | Service/process or management indicators | 51 farmacêuticos hospitalares e 31 comunitários | General population | Not reported | não informado | Not reported | Not reported |
| CAPES18 | Bandeira, 2023; Bandeira, 2024 | Brazil | Community pharmacy | — | Cross-sectional study | Não informado | CARACTERÍSTICAS DO SERVIÇO FARMACÊUTICO | Minor ailment management | Não citado explicitamente | Não descrito / Não aplicável. | Referral-based: written referral or occasional phone contact. | Humanistic outcomes; Service/process or management indicators | 428 farmacêuticos | General population | Adults | média 35 anos | Not reported | Not reported |
| 8 | Martins et al., 2025 | Brazil | Basic health unit | — | Qualitative study | novembro de 2024 a janeiro de 2025 | visitas farmacêutica | Health education | Não citado explicitamente | Protocolo Próprio / Desenvolvido pelos Autores | Integrated/transdisciplinary: joint care or real-time shared health record. | Not reported | 10 | Patients with diabetes | Older adults | — | Not reported | Not reported |
| 12 | Souza et al., 2025 | Brazil | Basic health unit; Community pharmacy | — | Experience report | — | educação em saúde | Health education | Não citado explicitamente | Protocolo Próprio / Desenvolvido pelos Autores | Isolated: independent practice. | Humanistic outcomes | — | Patients with diabetes | Not reported | — | Intervention/implementation adaptation | Disability |
| 14 | Vinholes et al., 2009 | Brazil | Other primary care setting | grupos preeexistentes de pacientes com hipertensão e diabetes, grupos da terceira idade e grupos de mulheres, pessoas vinculadas à Secretaria de Assistência Social da prefeitura e a postos de saúde da rede pública. | Experience report | junho de 2006 a setembro de 2007 | palestras | Health education | Uso Racional de Medicamentos (OMS) | Protocolo Próprio / Desenvolvido pelos Autores | Integrated/transdisciplinary: joint care or real-time shared health record. | Humanistic outcomes | 565 | General population | Mixed | > 40 anos | Not reported | Not reported |
| 15 | Ramos et al., 2023 | Brazil | Community pharmacy | — | Qualitative study | Junho de 2022 | prescrição farmacêutica | Other | Não citado explicitamente | Protocolo Próprio / Desenvolvido pelos Autores | Isolated: independent practice. | Not reported | 30 | General population | Adults | 28 a 45 anos | Not reported | Not reported |
| 27 | Durigon et al., 2023 | Brazil | Mixed setting | — | Qualitative study | abril de 2022 a julho de 2022 | práticas educativas | Health education | Uso Racional de Medicamentos (OMS) | Protocolo Próprio / Desenvolvido pelos Autores | Integrated/transdisciplinary: joint care or real-time shared health record. | Humanistic outcomes | 67 | General population | Adults | 31 a 40 anos | Not reported | Not reported |
| 39 | Melo et al., 2016 | Brazil | Basic health unit | — | Cross-sectional study | janeiro de 2011 a julho de 2012 | intervenções educativas \| Conciliação medicamentosa | Health education; Medication reconciliation | Não citado explicitamente | Protocolo Próprio / Desenvolvido pelos Autores | Referral-based: written referral or occasional phone contact. | Humanistic outcomes; Not reported | 1.164 | General population | Not reported | — | Intervention/implementation adaptation; Not reported | Disability; Age; Not reported |
| 48 | Oenning et al., 2011 | Brazil | Basic health unit | — | Cross-sectional study | Fevereiro a maio de 2007 | Dispensação | Clinically oriented dispensing | Não citado explicitamente | Protocolo Próprio / Desenvolvido pelos Autores | Integrated/transdisciplinary: joint care or real-time shared health record. | Service/process or management indicators; Humanistic outcomes | 111 | General population | Mixed | 15 a 40 anos | Not reported | Not reported |
| 50B | Amando et al., 2025 | Argentina | Community pharmacy | — | Before-and-after study (without control group) | — | Intervenção farmacêutica | Clinically oriented dispensing | Uso Racional de Medicamentos (OMS) | Escalas de Adesão (Morisky, Batalla, Haynes) | Referral-based: written referral or occasional phone contact. | Humanistic outcomes | 1481 | General population | Not reported | — | Not reported | Not reported |
| 54 | Miranda et al., 2022 | Ecuador | Other primary care setting | Não especificado | Before-and-after study (without control group) | — | Revisão do tratamento \| Intervenção farmacêutica | Medication review; Health education | Método Dáder / Seguimento (Escola de Granada) | Método Dáder / Seguimento de Granada | Isolated: independent practice. | Humanistic outcomes | 422 | General population | Older adults | 60 a 70 anos | Not reported | Not reported |
| 61B | Schneidera et al., 2022 | Brazil | Community pharmacy | — | Cross-sectional study | novembro de 2018 | rastrear risco de DM2 | Health screening | Não citado explicitamente | Critérios de Rastreio (Beers, STOPP/START, MAI) ( | Isolated: independent practice. | Clinical outcomes | 11.750 | General population | Mixed | 20 a 79 anos | Not reported | Not reported |
| 235 | Brito et al., 2009 | Brazil | Basic health unit | — | Before-and-after study (without control group) | janeiro de 2007 a agosto de 2008. | manejo farmacoterapêutico \| Ações educativas | Pharmacotherapeutic follow-up; Health education | Não citado explicitamente | Protocolo Próprio / Desenvolvido pelos Autores | Isolated: independent practice. | Clinical outcomes; Humanistic outcomes | 30 | Patients with hypertension | Older adults | 60 e 75 anos | Eligibility criterion | Age |
| OIASTER59,60,61 | Martínez-Mardones F, 2023 | Chile | Basic health unit | — | Randomized controlled trial | abril de 2017 a agosto 2020 | A cluster-randomized controlled trial | Pharmacotherapeutic follow-up | Método Dáder / Seguimento (Escola de Granada) | Método Dáder / Seguimento de Granada | Integrated/transdisciplinary: joint care or real-time shared health record. | Clinical outcomes | 324 | Patients with diabetes | Older adults | mais velhos que 65 anos | Not reported | Not reported |
| OIASTER59,60,62 | Martínez-Mardones F, 2024 | Chile | Basic health unit | — | Randomized controlled trial | — | A cluster-randomized controlled trial | Medication review | — | — | — | Humanistic outcomes | — | — | — | — | — | — |
| OIASTER59,60,63 | Martínez-Mardones F, 2025 | Chile | Basic health unit | — | Randomized controlled trial | — | A cluster-randomized controlled trial | — | — | — | — | Service/process or management indicators | — | — | — | — | — | — |
| OIASTER80 | Divaldo P, 2007 | Brazil | Basic health unit | — | Before-and-after study (without control group) | julho de 2003 a julho de 2004 | A longitudinal, prospective study (semi-experimental) | Pharmacotherapeutic follow-up | Método Dáder / Seguimento (Escola de Granada) | Método Dáder / Seguimento de Granada | Collaborative: shared case discussion and decision-making. | Humanistic outcomes | 30 | Patients with hypertension | Older adults | NE | Intervention/implementation adaptation | Age |
| OIASTER81 | Divaldo P, 2008 | Brazil | Basic health unit | — | Before-and-after study (without control group) | — | — | — | — | Escalas de Adesão (Morisky, Batalla, Haynes) | — | Service/process or management indicators | — | — | — | — | — | — |
| OIASTER82 | Divaldo P, 2009 | Brazil | Basic health unit | — | Before-and-after study (without control group) | — | — | — | — | — | — | — | — | — | — | — | — | — |
| OIASTER108 | Divaldo P, 2005 | Brazil | Basic health unit | — | Before-and-after study (without control group) | agosto de 2003 a julho de 2004. | intervenções farmacêuticas | Pharmacotherapeutic follow-up | Método Dáder / Seguimento (Escola de Granada) | Método Dáder / Seguimento de Granada | Isolated: independent practice. | Humanistic outcomes | 24 | General population | Older adults | superior a 60 anos | Other | Not reported |
| OIASTER109 | Divaldo P, 2006 | Brazil | Basic health unit | — | Before-and-after study (without control group) | — | — | — | — | — | — | Clinical outcomes | — | — | — | — | — | — |
| OIASTER110 | Divaldo P, 2007 | Brazil | Basic health unit | — | Before-and-after study (without control group) | — | — | — | — | — | — | Service/process or management indicators | — | — | — | — | — | — |
| OIASTER133 | Couto.B, 2006 | Brazil | Other primary care setting | o estudo foi realizado com 27 idosos da comunidade de periferia de Fortaleza (Comunidade do Dendê), pertencentes ao grupo social 'Cabelos Brancos' | Mixed-methods study | agosto de 2003 a junho de 2004. | Estudo longitudinal, quantitativo com enfoque qualitativo | Health education | Método Dáder / Seguimento (Escola de Granada) | Método Dáder / Seguimento de Granada | Isolated: independent practice. | Humanistic outcomes | 27 | General population | Older adults | superior a 60 anos | Intervention/implementation adaptation | Not reported |
| OIASTER134 | Couto.B, 2007 | Brazil | Other primary care setting | — | Mixed-methods study | — | — | — | — | — | — | Service/process or management indicators | — | — | — | — | — | — |
| OIASTER135 | Couto.B, 2008 | Brazil | Other primary care setting | — | Mixed-methods study | — | — | — | — | — | — | — | — | — | — | — | — | — |
| OIASTER152 | Neto P, 2011 | Brazil | Basic health unit | — | Non-randomized clinical trial / quasi-experimental study | janeiro de 2007 a dezembro de 2008 | foi realizada uma análise comparativa do padrão de prescrição de antimicrobianos no período de um ano antes e após a implantação do programa. | Health education | Atenção Farmacêutica (Hepler & Strand) | PWDT / Hepler & Strand (Pharmacotherapy Workup)Opção 2 | Collaborative: shared case discussion and decision-making. | Service/process or management indicators | 35.887 | General population | Mixed | NE | Other | Not reported |
| OIASTER153 | Neto P, 2012 | Brazil | Basic health unit | — | Non-randomized clinical trial / quasi-experimental study | — | — | Medication review | Atenção Farmacêutica (Hepler & Strand) | PWDT / Hepler & Strand (Pharmacotherapy Workup)Opção 2 | Integrated/transdisciplinary: joint care or real-time shared health record. | Clinical outcomes | — | — | — | — | — | — |
| OIASTER154 | Neto P, 2013 | Brazil | Basic health unit | — | Non-randomized clinical trial / quasi-experimental study | — | — | Clinically oriented dispensing | Atenção Farmacêutica (Hepler & Strand) | PWDT / Hepler & Strand (Pharmacotherapy Workup)Opção 2 | — | Economic outcomes | — | — | — | — | — | — |
| OIASTER166 | Ahumada-Canalea.A, 2021 | Chile | Basic health unit | — | Randomized controlled trial | janeiro de 2018 a julho de 2019 | Medication Review with Follow-up | Pharmacotherapeutic follow-up | Método Dáder / Seguimento (Escola de Granada) | Método Dáder / Seguimento de Granada | Collaborative: shared case discussion and decision-making. | Economic outcomes | 283 | Patients with cardiovascular disease | Older adults | pelo menos 65 anos | Other | Not reported |
| OIASTER167 | Ahumada-Canalea.A, 2022 | Chile | Basic health unit | — | Randomized controlled trial | — | — | Medication review | Farmácia Clínica (ESCP - Europeu)Opção 2 | PWDT / Hepler & Strand (Pharmacotherapy Workup)Opção 2 | — | Clinical outcomes | — | — | — | — | — | — |
| OIASTER168 | Ahumada-Canalea.A, 2023 | Chile | Basic health unit | — | Randomized controlled trial | — | — | Health education | Uso Racional de Medicamentos (OMS) | Não descrito / Não aplicável. | — | — | — | — | — | — | — | — |
| OIASTER241 | Andrade. R, 2005 | Brazil | Basic health unit | — | Randomized controlled trial | 12 meses | seguimento farmacoterapêutico | Pharmacotherapeutic follow-up | Atenção Farmacêutica (Hepler & Strand) | PWDT / Hepler & Strand (Pharmacotherapy Workup)Opção 2 | Referral-based: written referral or occasional phone contact. | Clinical outcomes | 44 | Patients with diabetes | Adults | a partir de 40 anos | Intervention/implementation adaptation | Not reported |
