# Supplementary material for: Clinical Services Provided by Pharmacists in Primary Health Care in Latin America: A Scoping Review
Source: J Eval Clin Pract. 2026 Nov 4;32(8):e70628. doi: 10.1111/jep.70628 (PMC13734460; doi:10.1111/jep.70628)
Supplement: Supplementary file 5 [file JEP-32-0-s005.docx]

Supplementary File 1. Full electronic search strategies

| **Metric** | **n** |
| --- | --- |
| Bibliographic databases, initial search | 1486 |
| Bibliographic databases, update | 115 |
| Bibliographic databases, total | 1601 |
| Gray literature sources, total | 600 |
| Total records before deduplication | 2201 |

# Bibliographic databases

## 1. BVS / LILACS

Date searched: 10 September 2025; updated 7 April 2026
Results: 548 initial; 45 update; 593 total.

("Atenção Primária à Saúde" OR "Atendimento Básico" OR "Atendimento Primário" OR "Atendimento Primário de Saúde" OR "Atenção Básica" OR "Atenção Primária" OR "Cuidado Primário de Saúde" OR "Cuidado de Saúde Primário" OR "Cuidados Primários" OR "Primeiro Nível de Assistência" OR "Primeiro Nível de Atendimento" OR "Primeiro Nível de Atenção" OR "Primeiro Nível de Cuidado" OR "Primeiro Nível de Cuidados" OR "Serviços de Saúde Comunitária" OR "Assistência Comunitária" OR "Assistência Médica Comunitária" OR "Assistência à Comunidade" OR "Assistência à Saúde Comunitária" OR "Cuidados Médicos Comunitários" OR "Diagnóstico Comunitário" OR "Medicina de Família e Comunidade" OR "Clínica de Família" OR "Medicina Familiar" OR "Medicina Familiar e Comunitária" OR "Medicina de Família" OR "Prática da Medicina Comunitária" OR "Prática da Medicina Familiar" OR "Prática da Medicina de Família" OR "Prática de Família" OR "Prática em Medicina Familiar" OR "Prática em Medicina de Família" OR "Primary Health Care" OR "Primary Care" OR "Community Health Services" OR "Community Health Service" OR "Ambulatory Care" OR "Outpatient Care" OR "Outpatient Health Services" OR "Outpatient Services" OR "Outpatient Service" OR "Urgent Care" OR "urgente cares" OR "Family Practice" OR "family practices")
AND
("Assistência Farmacêutica" OR "Atenção Farmacêutica" OR "Cuidados Farmacêuticos" OR "Serviços de Assistência Farmacêutica" OR "Servicios Farmacéuticos" OR "atención farmacéutica" OR "Serviços Comunitários de Farmácia" OR "Servicios Comunitarios de Farmacia" OR farmaceuticos OR farmaceutico OR "Serviço de Farmácia Hospitalar" OR "Serviço de Farmácia Clínica" OR "Servicio de Farmacia en Hospital" OR "servicio clínico de farmacia" OR "servicio de farmacia clínica" OR "servicio de farmacia hospitalaria" OR "Pharmaceutical Services" OR "Pharmacy Services" OR "Community Pharmacy Services" OR pharmacists OR pharmacist OR "Pharmacy Service, Hospital" OR " Pharmaceutic Services" OR "pharmaceutical care" OR "pharmacist interventions")
AND
(brasil OR "america latina" OR "america do sul" OR "america central" OR uruguai OR paraguai OR equador OR brazil OR "Latin America" OR "South America" OR argentina OR chile OR colombia OR mexico OR peru OR uruguay OR paraguay OR bolivia OR ecuador OR venezuela OR "Central America")

## 2. SciELO

Date searched: 10 September 2025; updated 7 April 2026
Results: 105 initial; 15 update; 120 total.

("Atenção Primária à Saúde" OR "Atendimento Básico" OR "Atendimento Primário" OR "Atendimento Primário de Saúde" OR "Atenção Básica" OR "Atenção Primária" OR "Cuidado Primário de Saúde" OR "Cuidado de Saúde Primário" OR "Cuidados Primários" OR "Primeiro Nível de Assistência" OR "Primeiro Nível de Atendimento" OR "Primeiro Nível de Atenção" OR "Primeiro Nível de Cuidado" OR "Primeiro Nível de Cuidados" OR "Serviços de Saúde Comunitária" OR "Assistência Comunitária" OR "Assistência Médica Comunitária" OR "Assistência à Comunidade" OR "Assistência à Saúde Comunitária" OR "Cuidados Médicos Comunitários" OR "Diagnóstico Comunitário" OR "Medicina de Família e Comunidade" OR "Clínica de Família" OR "Medicina Familiar" OR "Medicina Familiar e Comunitária" OR "Medicina de Família" OR "Prática da Medicina Comunitária" OR "Prática da Medicina Familiar" OR "Prática da Medicina de Família" OR "Prática de Família" OR "Prática em Medicina Familiar" OR "Prática em Medicina de Família" OR "Primary Health Care" OR "Primary Care" OR "Community Health Services" OR "Community Health Service" OR "Ambulatory Care" OR "Outpatient Care" OR "Outpatient Health Services" OR "Outpatient Services" OR "Outpatient Service" OR "Urgent Care" OR "urgente cares" OR "Family Practice" OR "family practices")
AND
("Assistência Farmacêutica" OR "Atenção Farmacêutica" OR "Cuidados Farmacêuticos" OR "Serviços de Assistência Farmacêutica" OR "Servicios Farmacéuticos" OR "atención farmacéutica" OR "Serviços Comunitários de Farmácia" OR "Servicios Comunitarios de Farmacia" OR farmaceuticos OR farmaceutico OR "Serviço de Farmácia Hospitalar" OR "Serviço de Farmácia Clínica" OR "Servicio de Farmacia en Hospital" OR "servicio clínico de farmacia" OR "servicio de farmacia clínica" OR "servicio de farmacia hospitalaria" OR "Pharmaceutical Services" OR "Pharmacy Services" OR "Community Pharmacy Services" OR pharmacists OR pharmacist OR "Pharmacy Service, Hospital" OR " Pharmaceutic Services" OR "pharmaceutical care" OR "pharmacist interventions")
AND
(brasil OR "america latina" OR "america do sul" OR "america central" OR uruguai OR paraguai OR equador OR brazil OR "Latin America" OR "South America" OR argentina OR chile OR colombia OR mexico OR peru OR uruguay OR paraguay OR bolivia OR ecuador OR venezuela OR "Central America")

## 3. PubMed/MEDLINE

Date searched: 10 September 2025; updated 7 April 2026
Results: 254 initial; 14 update; 268 total.

(("Primary Health Care"[MeSH Terms] OR "Primary Health Care"[Title/Abstract] OR "Primary Care"[Title/Abstract] OR "Community Health Services"[MeSH Terms] OR "community health service*"[Title/Abstract] OR "Ambulatory Care"[MeSH Terms] OR "Ambulatory Care"[Title/Abstract] OR "Outpatient Care"[Title/Abstract] OR "Outpatient Health Services"[Title/Abstract] OR "Outpatient Services"[Title/Abstract] OR "Outpatient Service"[Title/Abstract] OR "Urgent Care"[Title/Abstract] OR "Family Practice"[MeSH Terms] OR "family practice*"[Title/Abstract])
AND
("Pharmaceutical Services"[mh] OR "pharmaceutical service*"[tiab] OR "Pharmacy Service*"[tiab] OR "Community Pharmacy Services"[mh] OR Pharmacists[mh] OR pharmacist*[tiab] OR "Pharmacy Service, Hospital"[mh] OR " Pharmaceutic Service*"[tiab] OR "pharmaceutical care"[tiab]))
AND
(Brazil[tiab] OR "Latin America"[tiab] OR "South America"[tiab] OR Argentina[tiab] OR Chile[tiab] OR Colombia[tiab] OR Mexico[tiab] OR Peru[tiab] OR Uruguay[tiab] OR Paraguay[tiab] OR Bolivia[tiab] OR Ecuador[tiab] OR Venezuela[tiab] OR "Central America"[tiab])

## 4. Embase

Date searched: 10 September 2025; updated 7 April 2026
Results: 241 initial; 26 update; 267 total.

('primary health care'/exp OR 'primary health care':ti,ab,kw OR 'primary care':ti,ab,kw OR 'community care'/exp OR 'community health service*':ti,ab,kw OR 'ambulatory care'/exp OR 'ambulatory care':ti,ab,kw OR 'outpatient care':ti,ab,kw OR 'outpatient health services':ti,ab,kw OR 'outpatient services':ti,ab,kw OR 'outpatient service':ti,ab,kw OR 'urgent care':ti,ab,kw OR 'general practice'/exp OR 'family practice*':ti,ab,kw)
AND
('pharmaceutical service*':ti,ab,kw OR 'pharmacy service*':ti,ab,kw OR 'pharmacy (shop)'/exp OR 'pharmacist'/exp OR 'pharmacist*':ti,ab,kw OR 'hospital pharmacy'/exp OR 'pharmaceutic service*':ti,ab,kw OR 'pharmaceutical care':ti,ab,kw)
AND
('brazil':ti,ab,kw OR 'latin america':ti,ab,kw OR 'south america':ti,ab,kw OR 'argentina':ti,ab,kw OR 'chile':ti,ab,kw OR 'colombia':ti,ab,kw OR 'mexico':ti,ab,kw OR 'peru':ti,ab,kw OR 'uruguay':ti,ab,kw OR 'paraguay':ti,ab,kw OR 'bolivia':ti,ab,kw OR 'ecuador':ti,ab,kw OR 'venezuela':ti,ab,kw OR 'central america':ti,ab,kw)
AND
([embase]/lim OR [preprint]/lim OR 'clinical trial':dtype)

## 5. Scopus

Date searched: 10 September 2025; updated 7 April 2026
Results: 269 initial; 10 update; 279 total.

( TITLE-ABS-KEY ( "Primary Health Care" OR "Primary Care" OR "Community Health Services" OR "Community Health Service" OR "Ambulatory Care" OR "Outpatient Care" OR "Outpatient Health Services" OR "Outpatient Services" OR "Outpatient Service" OR "Urgent Care" OR "urgente cares" OR "Family Practice" OR "family practices" )
AND TITLE-ABS-KEY ( "Pharmaceutical Services" OR "Pharmacy Services" OR "Community Pharmacy Services" OR Pharmacists OR pharmacist OR "Hospital Pharmacy Service" OR " Pharmaceutic Services" OR "pharmaceutical care" OR "pharmacist interventions" )
AND TITLE-ABS-KEY ( Brazil OR "Latin America" OR "South America" OR Argentina OR Chile OR Colombia OR Mexico OR Peru OR Uruguay OR Paraguay OR Bolivia OR Ecuador OR Venezuela OR "Central America" ) )

## 6. CINAHL

Date searched: 10 September 2025; updated 7 April 2026
Results: 69 initial; 5 update; 74 total.

(TI ("Primary Health Care" OR "Primary Care" OR "Community Health Services" OR "Community Health Service" OR "Ambulatory Care" OR "Outpatient Care" OR "Outpatient Health Services" OR "Outpatient Services" OR "Outpatient Service" OR "Urgent Care" OR "urgente cares" OR "Family Practice" OR "family practices")
OR AB ("Primary Health Care" OR "Primary Care" OR "Community Health Services" OR "Community Health Service" OR "Ambulatory Care" OR "Outpatient Care" OR "Outpatient Health Services" OR "Outpatient Services" OR "Outpatient Service" OR "Urgent Care" OR "urgente cares" OR "Family Practice" OR "family practices")
OR SU ("Primary Health Care" OR "Primary Care" OR "Community Health Services" OR "Community Health Service" OR "Ambulatory Care" OR "Outpatient Care" OR "Outpatient Health Services" OR "Outpatient Services" OR "Outpatient Service" OR "Urgent Care" OR "urgente cares" OR "Family Practice" OR "family practices"))
AND
(TI ("Pharmaceutical Services" OR "Pharmacy Services" OR "Community Pharmacy Services" OR Pharmacists OR pharmacist OR "Pharmacy Service, Hospital" OR " Pharmaceutic Services" OR "pharmaceutical care" OR "pharmacist interventions")
OR AB ("Pharmaceutical Services" OR "Pharmacy Services" OR "Community Pharmacy Services" OR Pharmacists OR pharmacist OR "Pharmacy Service, Hospital" OR " Pharmaceutic Services" OR "pharmaceutical care" OR "pharmacist interventions")
OR SU ("Pharmaceutical Services" OR "Pharmacy Services" OR "Community Pharmacy Services" OR Pharmacists OR pharmacist OR "Pharmacy Service, Hospital" OR " Pharmaceutic Services" OR "pharmaceutical care" OR "pharmacist interventions"))
AND
(TI (Brazil OR "Latin America" OR "South America" OR Argentina OR Chile OR Colombia OR Mexico OR Peru OR Uruguay OR Paraguay OR Bolivia OR Ecuador OR Venezuela OR "Central America")
OR AB (Brazil OR "Latin America" OR "South America" OR Argentina OR Chile OR Colombia OR Mexico OR Peru OR Uruguay OR Paraguay OR Bolivia OR Ecuador OR Venezuela OR "Central America")
OR SU (Brazil OR "Latin America" OR "South America" OR Argentina OR Chile OR Colombia OR Mexico OR Peru OR Uruguay OR Paraguay OR Bolivia OR Ecuador OR Venezuela OR "Central America"))

# Gray literature sources

## 7. ProQuest Dissertations & Theses Citation Index

Date searched: 10 September 2025; updated 7 April 2026
Results: 3.

TS=(("Primary Health Care" OR "Primary Care" OR "Community Health Services" OR "Community Health Service" OR "Ambulatory Care" OR "Outpatient Care" OR "Outpatient Health Services" OR "Outpatient Services" OR "Outpatient Service" OR "Urgent Care" OR "urgente cares" OR "Family Practice" OR "family practices")
AND
("Pharmaceutical Services" OR "Pharmacy Services" OR "Community Pharmacy Services" OR pharmacists OR pharmacist OR "Pharmacy Service, Hospital" OR " Pharmaceutic Services" OR "pharmaceutical care" OR "pharmacist interventions")
AND
(brazil OR "Latin America" OR "South America" OR argentina OR chile OR colombia OR mexico OR peru OR uruguay OR paraguay OR bolivia OR ecuador OR venezuela OR "Central America"))

## 8. CAPES Theses and Dissertations Catalog

Date searched: 10 September 2025; updated 7 April 2026
Results: 19.

"Serviços Comunitários de Farmácia"

## 9. Brazilian Digital Library of Theses and Dissertations (BDTD)

Date searched: 10 September 2025; updated 7 April 2026
Results: 254.

("Atenção Primária à Saúde" OR "Atendimento Básico" OR "Atendimento Primário" OR "Atendimento Primário de Saúde" OR "Atenção Básica" OR "Atenção Primária" OR "Cuidado Primário de Saúde" OR "Cuidado de Saúde Primário" OR "Cuidados Primários" OR "Primeiro Nível de Assistência" OR "Primeiro Nível de Atendimento" OR "Primeiro Nível de Atenção" OR "Primeiro Nível de Cuidado" OR "Primeiro Nível de Cuidados" OR "Serviços de Saúde Comunitária" OR "Assistência Comunitária" OR "Assistência Médica Comunitária" OR "Assistência à Comunidade" OR "Assistência à Saúde Comunitária" OR "Cuidados Médicos Comunitários" OR "Diagnóstico Comunitário" OR "Medicina de Família e Comunidade" OR "Clínica de Família" OR "Medicina Familiar" OR "Medicina Familiar e Comunitária" OR "Medicina de Família" OR "Prática da Medicina Comunitária" OR "Prática da Medicina Familiar" OR "Prática da Medicina de Família" OR "Prática de Família" OR "Prática em Medicina Familiar" OR "Prática em Medicina de Família")
AND
("Assistência Farmacêutica" OR "Atenção Farmacêutica" OR "Cuidados Farmacêuticos" OR "Serviços de Assistência Farmacêutica" OR "Servicios Farmacéuticos" OR "atención farmacéutica" OR "Serviços Comunitários de Farmácia" OR "Servicios Comunitarios de Farmacia" OR farmaceuticos OR farmaceutico OR "Serviço de Farmácia Hospitalar" OR "Serviço de Farmácia Clínica" OR "Servicio de Farmacia en Hospital" OR "servicio clínico de farmacia" OR "servicio de farmacia clínica" OR "servicio de farmacia hospitalaria")
AND
(brasil OR "america latina" OR "america do sul" OR "america central" OR Uruguai OR Paraguai OR Equador OR argentina OR chile OR colombia OR mexico OR peru OR bolivia OR venezuela)

## 10. Open Access Theses and Dissertations (OATD)

Date searched: 10 September 2025; updated 7 April 2026
Results: 46.

("Pharmaceutical Services" OR "Pharmacy Services" OR "Community Pharmacy Services" OR Pharmacists OR pharmacist OR "Hospital Pharmacy Services" OR " Pharmaceutic Services" OR "pharmaceutical care" OR "pharmacist interventions")
AND
(Brazil OR "Latin America" OR "South America" OR Argentina OR Chile OR Colombia OR Mexico OR Peru OR Uruguay OR Paraguay OR Bolivia OR Ecuador OR Venezuela OR "Central America")

## 11. EBSCO Open Dissertations

Date searched: 10 September 2025; updated 7 April 2026
Results: 16.

("Pharmaceutical Services" OR "Pharmacy Services" OR "Community Pharmacy Services" OR Pharmacists OR pharmacist OR "Hospital Pharmacy Services" OR " Pharmaceutic Services" OR "pharmaceutical care" OR "pharmacist interventions")
AND
(Brazil OR "Latin America" OR "South America" OR Argentina OR Chile OR Colombia OR Mexico OR Peru OR Uruguay OR Paraguay OR Bolivia OR Ecuador OR Venezuela OR "Central America")

## 12. OAISTER

Date searched: 10 September 2025; updated 7 April 2026
Results: 262.

("Pharmaceutical Services" OR "Pharmacy Services" OR "Community Pharmacy Services" OR Pharmacists OR pharmacist OR "Hospital Pharmacy Services" OR " Pharmaceutic Services" OR "pharmaceutical care" OR "pharmacist interventions")
AND
(Brazil OR "Latin America" OR "South America" OR Argentina OR Chile OR Colombia OR Mexico OR Peru OR Uruguay OR Paraguay OR Bolivia OR Ecuador OR Venezuela OR "Central America")
